# A Proteomic Signature for Human Papillomavirus–Associated Oropharyngeal Squamous Cell Carcinoma Predicts Patients at High Risk of Recurrence

**DOI:** 10.1158/2767-9764.CRC-23-0460

**Published:** 2025-04-09

**Authors:** Christopher C. Jackson, Jia (Jenny) Liu, Howard Y. Liu, Steven G. Williams, Asim Anees, Zainab Noor, Natasha Lucas, Dylan Xavier, Peter G. Hains, Daniel Bucio-Noble, Adel T. Aref, Sandro V. Porceddu, Rahul Ladwa, Joseph Whitfield, Roger R. Reddel, Qing Zhong, Benedict J. Panizza, Phillip J. Robinson

**Affiliations:** 1 Department of Otolaryngology, Head and Neck Surgery, Princess Alexandra Hospital, Brisbane, Australia.; 2 Queensland Head and Neck Cancer Centre, Princess Alexandra Hospital, Brisbane, Australia.; 3 Faculty of Medicine, University of Queensland, Brisbane, Australia.; 4 ProCan, Children’s Medical Research Institute, The University of Sydney, Sydney, Australia.; 5 The Kinghorn Cancer Centre, St. Vincent’s Hospital, Darlinghurst, Australia.; 6 School of Clinical Medicine, Faculty of Medicine & Health, University of New South Wales, Sydney, Australia.; 7 Department of Cancer Services, Princess Alexandra Hospital, Brisbane, Australia.; 8 Pathology Queensland, Princess Alexandra Hospital, Brisbane, Australia.

## Abstract

Although human papillomavirus (HPV)–positive oropharyngeal squamous cell carcinoma (OPSCC) is associated with better prognosis than HPV-negative disease, ∼30% of cases relapse despite curative-intent radiotherapy (±chemotherapy). We aimed to develop a proteomic signature associated with risk of recurrence within HPV+OPSCC. We analyzed tumor specimens from 124 patients with T1–4N0–3M0 HPV+OPSCC: 50 patients with residual or recurrent disease within 5 years of treatment and 74 age and performance status–matched patients with no recurrence. Proteomic analysis was performed on archival formalin-fixed, paraffin-embedded primary tumor core biopsy specimens and matched normal adjacent tissues using quantitative data-independent acquisition mass spectrometry. Recurrence-free survival (RFS), both locoregional and distant, was the primary endpoint. Univariate Cox regression analysis identified peptides associated with RFS, from which a risk score was established to generate a peptide-based signature. A total of 7,597 protein groups were identified across the cohort, 1,565 of which were differentially abundant between tumor and normal adjacent tissues, with 1,218 being significantly increased in tumors. Improved 5-year RFS (*q*-value < 0.5) was associated with 405 differentially abundant peptides (from 233 unique proteins) within the 124 tumors. Among them a 26-peptide signature encompassing 26 protein groups was associated with RFS and was able to stratify patients into low, intermediate, or high risk of recurrence (concordance index = 0.941, *P* < 0.0001). Data available via ProteomeXchange PXD036891. Overall, a 26-peptide signature can be used to risk-stratify HPV+OPSCC. Validation of this proteomic prognostic signature in an independent cohort is required to assess its potential use in future clinical trials to better tailor initial therapy.

**Significance:**

HPV+OPSCC incidence is increasing, with heterogeneous treatment outcomes despite favorable prognosis. Current de-escalation strategies show inferior results, highlighting the need for precise risk stratification. Using data-independent acquisition mass spectrometry proteomics, we identified a 26-peptide signature that stratifies patients into risk categories, potentially enabling personalized treatment decisions and optimal patient selection for de-escalation trials.

## Introduction

The incidence of human papillomavirus–associated oropharyngeal squamous cell carcinoma (HPV+OPSCC) has increased significantly in recent decades (1). This increase has been observed predominantly in younger men with no smoking history (2). Patients typically have locoregionally advanced disease at diagnosis and are treated with definitive radiation (±chemotherapy) or surgery with curative intent where indicated (3–5). High response rates to this standard of care treatment are associated with a favorable 5-year overall survival (OS) of approximately 85%, dependent on tumor stage (6, 7). Despite this 5-year OS, up to 30% of patients will experience disease recurrence. Although some of these can be salvaged, many will live past 5 years with incurable disease managed using systemic therapy.

Currently, all patients receive the same standardized treatment protocol, and consequently are exposed to the same treatment-related toxicities, regardless of their underlying risk of recurrence. Potential treatment-associated toxicities include mucositis, xerostomia, dysphagia, gastrostomy tube dependence, and osteoradionecrosis (8). The addition of cisplatin-based chemotherapy is further associated with additive irreversible ototoxicity, neurotoxicity, and nephropathy (9). Younger age at diagnosis together with high and durable response rates means that any morbidity may be experienced for several decades (8). The importance of survivorship care has motivated consideration of treatment de-escalation protocols to de-intensify treatment without compromising therapeutic efficacy (10). Currently no phase III trial of radiation dose reduction has been published as initial phase II trials have failed to satisfy noninferiority to current treatment (11). De-escalation trials of systemic treatment have to date commonly resulted in a loss of clinical equipoise in favor of standard treatment (5, 7, 12). Identification of the low-risk patient subset suitable for treatment de-escalation remains relevant.

De-intensification trials have historically relied on clinicopathologic variables to target low-risk individuals at the recruitment stage (11, 13). Commonly used variables include disease extent at diagnosis [tumor–node–metastasis (TNM) staging system], smoking exposure (pack/years), or a combination of the two (i.e., Ang classification; ref. 14). Although these variables are established independent predictors of inferior recurrence-free survival (RFS) and OS, they are suboptimal predictors of treatment efficacy in the absence of biological tumor insight (14–17). Moreover, despite the favorable prognosis for many patients with HPV+OPSCC, outcomes are heterogeneous and prognostic biomarkers to identify patients with more aggressive biology in need of treatment escalation and closer surveillance are urgently required (13, 18).

Both gene expression and proteomic signatures have been explored as prognostic biomarkers of HPV+OPSCC in recent years. Previous gene expression profiling in HPV+OPSCC identified 60- (4) and 38-gene (19) risk signatures (which have no overlap). Although they are robust at discriminating between high- and low-risk patients with HPV+OPSCC, neither has been adopted into clinical practice because of limitations in the ability to perform timely, cost-effective analyses. It is expected that proteomic-derived biomarkers will better reflect the cancer phenotype because of posttranscriptional modifications (20). Recently, a proteomic analysis comparing recurrent HPV+OPSCC and matched normal formalin-fixed paraffin-embedded (FFPE) samples identified 77 differentially abundant proteins (DAProt) among 1,414 protein groups detected in a 40 patient cohort but did not seek for a molecular signature for patient stratification for RFS or OS (21). Thus, there remains an unmet need for robust prognostic biomarkers to classify patients with HPV+OPSCC according to risk of recurrence.

We aimed to develop a pretreatment, prognostic proteomic signature to identify patients with HPV+OPSCC at high risk of recurrence using a validated, high-throughput proteomic analysis pipeline. Quantitative data-independent acquisition mass spectrometry (DIA-MS) was performed on FFPE primary tumor core biopsy specimens. As the process of protein inference from primary peptide data results in information loss due to various assumptions made to produce imputed intensity values for protein-level information, we focused on a peptide-only signature approach because it uses only direct intensity values and thus more accurately represents the quantitative DIA-MS data (22–25).

## Materials and Methods

### Patients

This retrospective analysis included patients with HPV+OPSCC treated with curative-intent radiotherapy (RT), alone or in combination with chemotherapy between 2007 and 2019 at the Princess Alexandra Hospital (PAH). Clinicopathologic, treatment, and outcome data were retrieved from a prospectively collated database. This study was performed in accordance with the Declaration of Helsinki principles. Ethical approval for this study was obtained through the Metro South Human Research Ethics Committee at the PAH (HREC/14/QPAH/54), and written informed consent was obtained from the patients.

**Figure 1:**
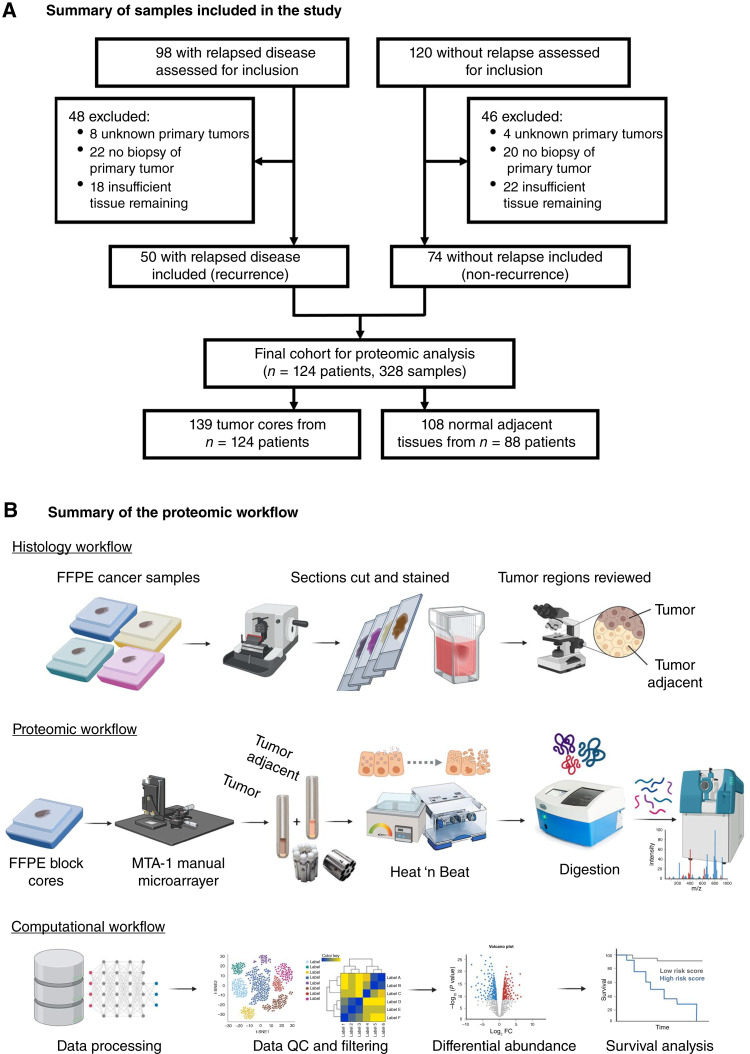
Study samples and workflow. **A,** Summary of samples included in the study. **B,** Summary of the proteomic workflow. In brief, tumor core and NAT samples underwent high pressure and temperature treatment for lysis and digestion of proteins to peptides by trypsin/LysC. Samples were analyzed in duplicate via DIA-MS in two separate instruments. Proteomic data was processed using DIA-NN software, and quantitative data on peptides was obtained. Protein abundance was inferred, and single-peptide proteins present in ≥20% of the samples were included in the downstream analyses.

A total of 98 patients with HPV+OPSCC with either evidence of posttreatment residual disease or recurrence within 5 years were identified. An additional 120 patients without evidence of relapse at 5-year follow-up were also selected. Eligible patients had HPV+OPSCC defined by positive p16 IHC; T1-4N0-3M0 stage per American Joint Committee on Cancer eighth edition; and the availability of sufficient archived FFPE primary tumor material (obtained from pretreatment diagnostic biopsies) for mass spectrometry (MS) analysis. A total of 124 patients met these inclusion criteria: 50 patients with recurrent and 74 with nonrecurrent disease (Fig. 1A). All patients underwent definitive intensity-modulated RT with the majority receiving 70 Gy delivered to gross disease over 7 weeks. Concurrent systemic chemotherapy was recommended for T3/T4 disease or bulky nodal disease (multiple nodes or single node ≥3 cm). This consisted of high dose cisplatin (100 mg/m^2^) delivered on weeks 1, 4, and 7 or weekly concurrent “low-dose” cisplatin (40 mg/m^2^). Patients with comorbidities contraindicating cisplatin administration received either no concurrent therapy or a cetuximab anti-EGFR mAb at a 400 mg/m^2^ loading dose and subsequent 250 mg/m^2^ weekly dose.

### Histology workflow

FFPE primary tumor samples were sectioned at 10 µm thickness and stained with hematoxylin and eosin to identify tumor and normal adjacent tissues (NAT; Fig. 1B). Tissue sampling for proteomics was performed with an MTA-1 manual microarrayer (Beecher Instruments), yielding 1 × 1 mm sample cores. Following histopathologist review, a total of 139 tumor cores were sampled from 124 patients. Eighty-eight patients had sufficient NATs from which 108 samples were taken. Fifteen tumor and 20 NAT biological replicates were included, and proteomic results were averaged for biological replicates. All samples were acquired in technical duplicate and were randomized according to three variables within experimental batches (*n* = 15 per batch). The variables were recurrence (R)/nonrecurrence (NR) status, TNM stage, and smoking category (never/former >12 months vs. current/former <12 months).

Tissue lysis and digestion was performed by an adaptation of our previous Accelerated Barocycler Lysis and Extraction (ABLE) method for fresh frozen samples (26), adapted to suit FFPE samples, and referred to as Heat ‘n Beat (Supplementary Methods; ref. 27). The digested peptide yield was normalized to a concentration of 0.5 µg/µL to give 2 µg MS load in 4 µL.

### Proteomic workflow

Proteomic data was acquired by DIA-MS, across six SCIEX TripleTOF 6600 mass spectrometers interfaced with Ekspert NanoLC 425 (Eksigent) high performance liquid chromatography systems operating in microflow mode (Fig. 1B; see Supplementary Data S1 for details on the MS run and data filtering settings). From the tumor and NAT samples, 643 DIA-MS runs passed quality control criteria, and 123 HEK293 cell line lysates were acquired as Heat ‘n Beat peptide preparation and MS instrument controls. Sample runs were used to generate a spectral reference library using DIA-NN software (28) and a UniProt FASTA database (UniProt Release 2021_03; 20,612 sequences) with retention time peptides. The spectral reference library contained 8,563 protein groups and 59,599 precursors. It was used to search a total of 766 DIA-MS files with DIA-NN, including HEK293 cell line controls.

### Computational workflow

The primary data collected by DIA-MS refers only to peptides and their relative abundances; however, a higher level of proteomic data involves grouping or “roll-up” to map single or multiple peptides to proteins, in which the peptide abundances are combined to infer the corresponding relative protein abundances. The following analysis will refer to the peptide-only approach, with the protein roll-up displayed in the Supplementary Data S1. A “Sequence Coverage Report” was prepared to show the distribution in the protein sequence for each peptide in the signature, the average peptide intensity detected by DIA-MS, and the percent of sample runs in which the peptide was not detected (Supplementary Word File - Sequence Coverage Report).

Differentially abundant peptide (DAPep) or DAProt analysis between tumor and NAT samples, and between samples from R and NR tumor groups, was performed on all identified peptides and proteins. Empirical Bayes moderated t-statistics [limma R package from Bioconductor (29)] were used to compute the statistical significance of relative protein expression between the two classes. Tumor-specific significantly expressed proteins were selected at Benjamini–Hochberg–adjusted *P* value < 0.01, with a log fold-change (FC; expressed as difference in the group means) cut-off of ±0.5 (FC >1.5 and <0.67), whereas recurrence-specific significant peptides and proteins were selected with a log FC (expressed as difference in the group means) cut-off of ±0.1. RFS was the primary endpoint of interest and was defined as time (in months) from treatment commencement to identification of either residual or recurrent disease within the 5-year follow-up period. All recurrence events (local, nodal, and metastatic) were combined for this analysis. When recurrence occurred at multiple sites, time to the first site of recurrence was used. The secondary endpoint was the OS, defined as duration from treatment commencement to death from any cause. Patients were censored at the date of last follow-up (>60 months after treatment) in the absence of an event occurring.

To control for the error rate among tests, FDR, as summarized in a q value, was set at 1%. FDR correction ensures that the error rate among the significant results remains controlled, minimizing the risk of false positives while maximizing the detection of true positives. Univariate Cox proportional hazard modeling (30) was performed followed by 100 runs of multivariate Cox regression (31, 32) with least absolute shrinkage and selection operator (Lasso) regularization using 20-fold cross-validation to generate a list of signature peptides. Using these peptides, a risk score was calculated. Details of survival analysis are reported elsewhere (33).

To assess performance of the signature, a concordance index (C-index) from the multivariate analysis was computed (34, 35). Time-to-event disease endpoint analysis is depicted as Kaplan–Meier (KM) curves. Performance of the proteomic signature was further assessed in comparison with important clinical covariates through a multivariate Cox model as well as by comparing AUCs of their time-dependent ROC curves at 5-year time point. Important clinical variables included age, gender, Eastern Cooperative Oncology Group performance status, T-stage, N-stage, and smoking category [as defined by Liu and colleagues (18)].

Downstream biological and cellular pathways were analyzed in the Reactome database (36) using Cytoscape (36) for the proteins of interest. Plots were based on the Reactome database and created from Reactome Pathway Analysis (ReactomePA, https://bioconductor.org/packages/release/bioc/html/ReactomePA.html) using the enrichPathway R package (https://github.com/YuLab-SMU/ReactomePA/blob/master/R/enrichPathway.R). Individual enrichment barplots were generated using R package enrichplot (https://github.com/YuLab-SMU/enrichplot). Data visualization and plotting were performed using ggplot2 R package (https://r-graph-gallery.com/ggplot2-package.html).

### Data availability

The MS proteomic data have been deposited to the ProteomeXchange Consortium via the PRIDE partner repository with the dataset identifier PXD036891.

## Results

### Patient characteristics

Clinicopathologic characteristics and outcome data of the 124 patients (Fig. 1A) are summarized in Table 1. Median time to recurrence was 1 year, and for the patients that did not have a recurrence, the median follow-up was 5.9 years. Five (10%) R and two NR tumors (2.7%) were treated with RT alone. The remaining patients received RT with concurrent cisplatin-based chemotherapy (or cetuximab). Significantly more R groups of patients were administered cetuximab (42% vs. 18.9%, *P* = 0.001) because of contraindications to cisplatin. In comparison with NR, the R group of patients were also slightly older (mean of 3 years) at diagnosis (*P* = 0.01), with a more advanced nodal stage (*P* = 0.05) and were treated with a lower RT dose (*P* = 0.01). Thirty-four (64%) deaths were recorded in the R cohort; 28 (56%) were due to OPSCC. Univariate Cox proportional hazard analysis confirmed that nodal status, RT dose, and the systemic therapy agent were significantly associated with RFS (Table 1).

**Table 1:** Summary of the clinicopathologic characteristics of the OPSCC cohort

Characteristic	Total	R (*n* = 50)	NR (*n* = 74)	*P* value
Median (range)				
Age at diagnosis, years, mean (range)	59.5 (40–89)	61 (45–89)	58 (40–78)	***P* = 0.01**
Number of patients, *n* (%)				
Gender				*P* = 1
Male	113 (91.1)	46 (92)	67 (90.5)	
Female	11 (8.9)	4 (8)	7 (9.5)	
ECOG status				*P* = 0.88
0	88 (71)	32 (64)	56 (75.7)	
1	26 (21)	12 (24)	14 (18.9)	
2	9 (7.3)	5 (10)	4 (5.4)	
3	1 (0.8)	1 (2)	—	
Smoking status				*P* = 0.83
Never	36 (29)	13 (26)	23 (31.1)	
Current	23 (18.5)	10 (20)	13 (17.6)	
Former	65 (52.4)	27 (54)	38 (51.4)	
Smoking (pack/years)				*P* = 0.36
≤10	58 (46.8)	20 (40)	38 (51.4)	
>10	66 (53.2)	30 (60)	36 (48.6)	
Primary site				*P* = 0.64
Base of the tongue	45 (36.3)	20 (40)	25 (33.8)	
Tonsil	49 (39.5)	20 (40)	29 (39.2)	
Other	30 (24.2)	10 (20)	20 (27.0)	
T stage (eighth edition TNM)				*P* = 0.06
T1	21 (16.9)	2 (4)	19 (25.7)	
T2	45 (36.3)	21 (42)	24 (32.4)	
T3	28 (22.6)	14 (28)	14 (18.9)	
T4	30 (24.2)	13 (26)	17 (23)	
N stage (eighth edition TNM)				***P* = 0.05**
N0	5 (4)	2 (4)	3 (4.1)	
N1	72 (58.1)	24 (48)	48 (64.9)	
N2	31 (25)	14 (28)	17 (23)	
N3	16 (12.9)	10 (20)	6 (8.1)	
AJCC stage				*P* = 0.21
I	51 (41.1)	16 (32)	35 (47.3)	
II	34 (27.4)	15 (30)	19 (25.7)	
III	39 (31.5)	19 (38)	20 (27)	
RT dose (Gy)				***P* = 0.01**
66	3 (2.4)	2 (4)	1 (1.4)	
68	12 (9.7)	9 (18)	3 (4.1)	
70	109 (87.9%)	39 (78%)	70 (94.6)	
Systemic therapy				***P* = 0.001**
None	7 (5.6)	5 (10)	2 (2.7)	
Cisplatin	82 (66.1)	24 (48)	58 (78.4)	
Cetuximab	35 (28.2)	21 (42)	14 (18.9)	
Failure site				
Local	9	9 (18)	—	
Nodal	22	22 (44)	—	
Metastatic	19	19 (38)	—	
Median time to failure (months)				
Local	—	18	—	
Nodal	—	13	—	
Metastatic	—	14	—	
Median follow-up (months)	—	—	70.8	
Disease status at last follow-up				
ANED	81 (65.3)	7 (14)	74 (100)	
AWD	9 (7.3)	9 (18)	—	
DOD	28 (22.6)	28 (56)	—	
DWD	6 (4.8)	6 (12)	—	

Data are expressed as *n* (%, of each group).

Abbreviations: AJCC, American Joint Committee on Cancer; ANED, alive no evidence of disease; AWD, alive with disease; DOD, died of disease; DWD, died with disease; ECOG, Eastern Cooperative Oncology Group; Gy, gray; NR, nonrecurrent; R, recurrence.

### Proteomic classification model development

**Figure 2:**
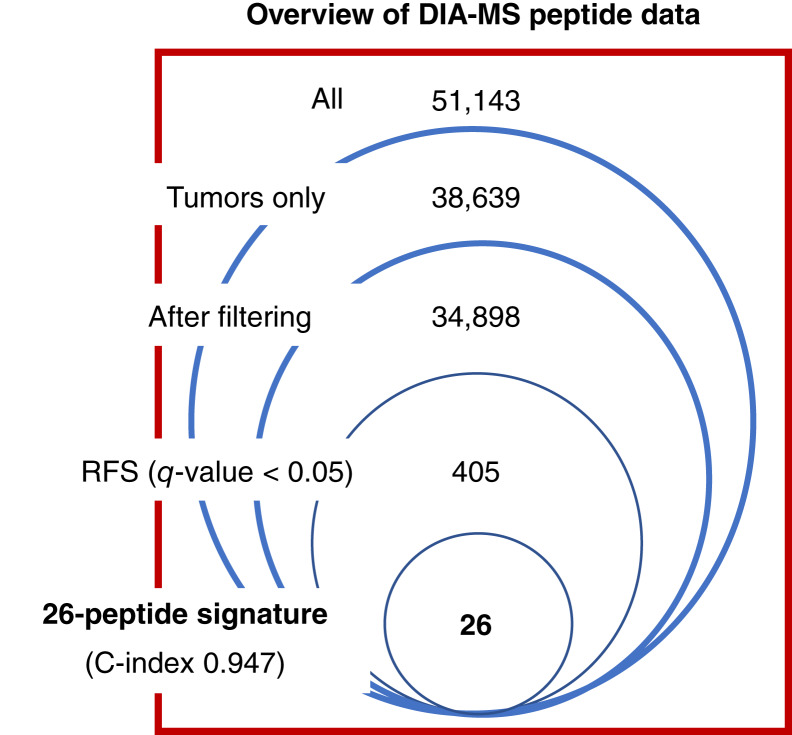
Overview of the DIA-MS dataset. Number of peptides and proteins identified using DIA-MS samples from all 124 patients (“All” includes both tumor and NATs). The number of peptides is shown in each filtering step with 405 being associated with RFS (*q* value < 0.05) and used to obtain the prognostic signature. In the peptide intensity filtering step, peptides with ≤15 raw intensity value were discarded. The C-index of the DAPep signature from multivariate Cox modeling was 0.947.

A summary of the proteomic workflow is shown in Fig. 1B. This study primarily focused on individual DAPeps, and they are referred to herein as the protein group in which each peptide is uniquely present. From 643 DIA-MS runs, 51,143 peptides from 7,597 protein groups were identified from both tumor and NAT samples (Fig. 2). Of these, 38,639 peptides from 5,903 protein groups were quantified only in tumor samples. Proteins quantified between technical replicates of each sample yielded a median Pearson correlation coefficient of 0.91. After filtering, 34,898 peptides corresponding to 5,199 protein groups were quantified.

#### Comparison between tumor and normal samples

Comparison of peptides between matched tumor and NAT samples revealed 4,834 DAPeps (from 1,565 protein groups) overall. Among them, 2,816 DAPeps (from 1,218 protein groups) were significantly upregulated, and 2,018 (from 396 proteins) were downregulated in the tumor samples at an adjusted *P* value of 0.01 (1% FDR; Supplementary Fig. S1A; volcano plot, Supplementary Table S1). A heatmap analysis of DAPeps in tumor and NAT samples shows peptides primarily clustered according to the tumor or NATs (Supplementary Fig. S1B). Cellular pathways involved in the top tumor-enriched peptides include mRNA metabolism and splicing, DNA synthesis, and cell cycle–associated pathways (Supplementary Fig. S2A). The pathways downregulated in the tumor samples include muscle function, extracellular matrix interaction, and pathways associated with platelets and clotting (Supplementary Fig. S2B). The results are consistent with the tissue origin of both cohorts and represent a clear discrimination of tumor and NATs.

#### Comparison between R and NR

**Figure 3:**
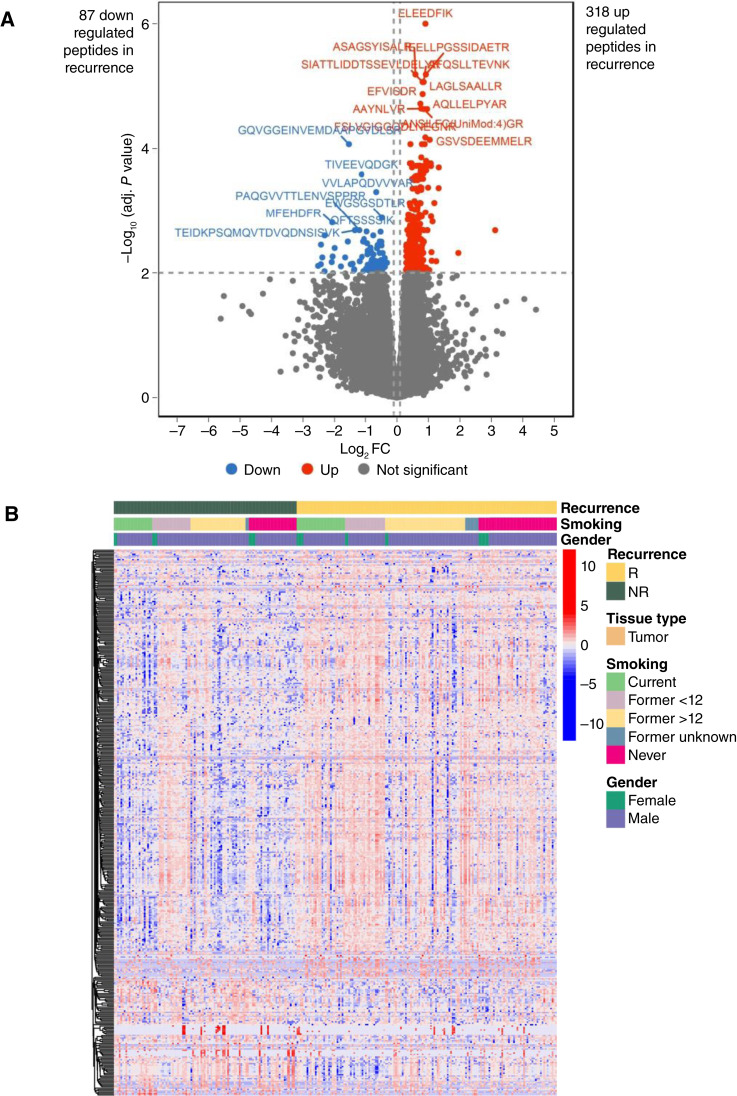
Differential abundance of 233 unique protein groups identified from 405 DAPeps discriminates R from NR. **A,** Volcano plot shows the distribution of 233 proteins significantly up- or downregulated in the R relative to NR in samples of patients with HPV+OPSCC. A total of 87 peptides (from 52 unique protein groups) were downregulated in R, and 318 peptides (from 181 unique protein groups) were upregulated in R at an adjusted *P* value of 0.01 (1% FDR). Axes show FC > 1.5 and adjusted *P* value (adj. P Val) < 0.01. Significantly DAPeps are indicated in red (increased in R) or blue color (decreased in R). Most peptides showed low FC after differential expression analysis (colored gray). **B,** Heatmap representation of the *z* scores obtained after unsupervised hierarchical clustering of the DAPeps from **A**. R and two NR tumors only. Expression data are converted to *z* scores. Samples are shown on the *x*-axis, whereas peptides are clustered on the *y*-axis against RFS or some clinicopathologic variables. Smoking status former <12 or >12 months.

We next focused only on the 124 tumor-rich core samples. About one third of the 34,898 filtered peptides were detected in >90% of all samples, with the vast majority being detected in >20% of all samples and MS runs (Supplementary Fig. S3). This indicates reproducible detection in 248 MS runs and shows their widespread distribution across the tumor cohort. To identify potential biomarkers that are prognostic for recurrence, differential abundance analysis was done between the R and NR groups. In the NR group, 87 peptides were downregulated and 318 peptides were upregulated at an adjusted *P* value of 0.01 (Fig. 3A, volcano plot). The peptides and their corresponding protein groups are listed in Supplementary Table S2, noting that some of the peptides derived from the same protein (leaving 52 unique downregulated proteins, 181 unique upregulated proteins and a total of 233 unique differentially regulated protein groups). A heat map of DAPeps shows two major clusters (Fig. 3B). A number of functional cellular pathways were associated with DAPeps in R and NR groups of patients (Supplementary Fig. S4). Pathways for upregulated DAPeps in R included T-cell receptor signaling, adaptive and innate immune systems, and JAK–STAT signaling after IL-12 stimulation (Supplementary Fig. S4A). The top 20 biological process gene ontology terms relate to immune responses (including Fc receptor signaling) or actin dynamics. This included six proteins from the Arp2/3 complex involved in actin nucleation (ARPC3, ARPC2, ARPC5L, ACTR2, ARPC4, and ARPC1B); 13 proteins from the RHO GTPases, WASP, WAVE, and gelsolin families (ARPC3, COTL1, ACTR3, ARPC2, WAS, ACTR2, TMSB4X, WIPF1, EVL, LCP1, ARPC4, ARPC1B, and WDR1); four proteins in MHC protein complex assembly (CD74, HLA-DQB1, TAPBP, and HLA-DPB1); and nine proteins from the proteasome complex (PSMC4, PSME2, KATNAL2, PSMB4, PSMB10, PSMB9, PSMB8, PSME1, and PSMD14). The pathways for the 87 downregulated DAPeps in NR (which are upregulated in R) featured roles in core extracellular matrix functions, integrin signaling, platelet degranulation, and cellular hemostasis (Supplementary Fig. S4B). The top five biological process Gene Ontology terms all relate to cell adhesion (20 proteins)/extracellular matrix organization (5 proteins) or tissue development (29 proteins)/tissue morphogenesis (11 proteins). The overall results clearly discriminate NR from R by elevation of immune responses and actin dynamics in NR, and suppression of the tumor microenvironment and extracellular matrix in R.

### A 26-peptide signature associated with RFS stratifies patients into three risk groups

**Figure 4:**
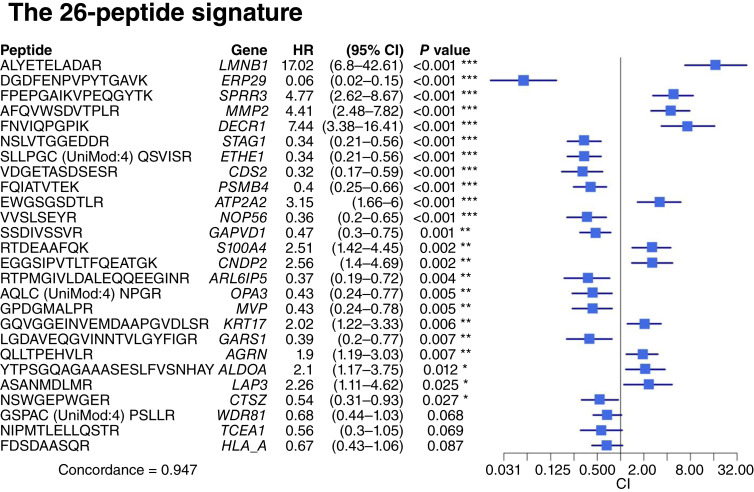
The 26-peptide signature. List of the 26-peptide signature (amino acid sequence and predicted gene name) with HRs, 95% CIs, and *P* values in the multivariate model, ranked according to their significance from top to bottom. The signature was identified by Lasso-regularized multivariate Cox proportional hazard model as input to a multivariate Cox model with a forward feature selection algorithm and has a C-index of 0.947. The squares and intervals in the plot represent the values of HRs and CIs, respectively. Each peptide is from a different protein group. UniMod:4 is an iodoacetamide derivative of cysteine.

To identify a peptide signature associated with RFS, multiple runs of Lasso-regularized multivariate Cox regression were performed on the 405 DAPeps from R versus NR (*q* value < 0.05). This revealed a list of 26 peptides with a C-index (34, 35) of 0.947, indicating robust prognostic power from these peptides (Fig. 4). The 26 peptides are from 26 different protein groups (Supplementary Table S3), all of which were identified by multiple additional peptides in MS analysis, and none were single-peptide proteins (shown in the Supplementary Word File - Sequence Coverage Report). This analysis provides a high level of confidence in the identification and quantitation of these peptides.

A patient’s risk score was calculated as the sum of the intensities of each of the 26 peptides, multiplied by the corresponding regression coefficient. The risk score’s range (minimum to maximum) was divided into three equal subranges. Patients were stratified into low, medium, or high risk according to these subranges. This two-step process gave rise to a 26-peptide signature.

**Figure 5:**
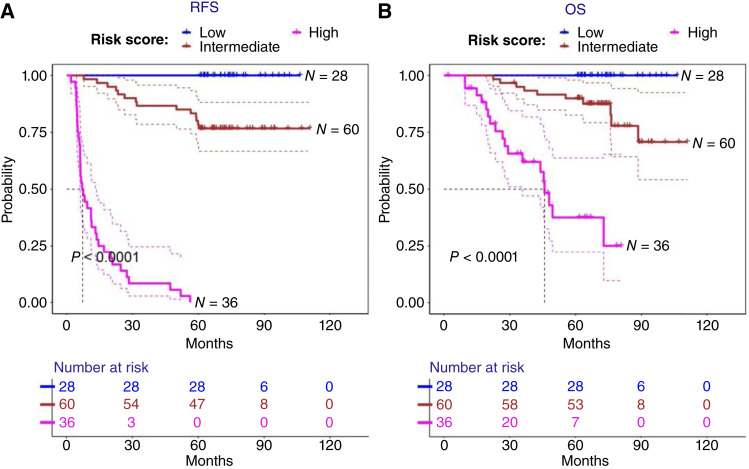
A 26-peptide signature stratifies patients with HPV+OPSCC into three risk groups for RFS or OS. KM curves with 95% CIs based on the 26-peptide signature risk stratification of patients with HPV+OPSCC are shown. The signatures were separated into low-, intermediate- and high-risk groups for (**A**) RFS or (**B**) OS based on the median cut-off, and the respective numbers of samples in each risk group are shown. The log rank test was used to assess the *P* value of the differences between the KM curves. The signatures were built using the coefficients from the training dataset. The shaded area around each curve represents 95% CI. The dotted lines mark median probabilities.

**Figure 6:**
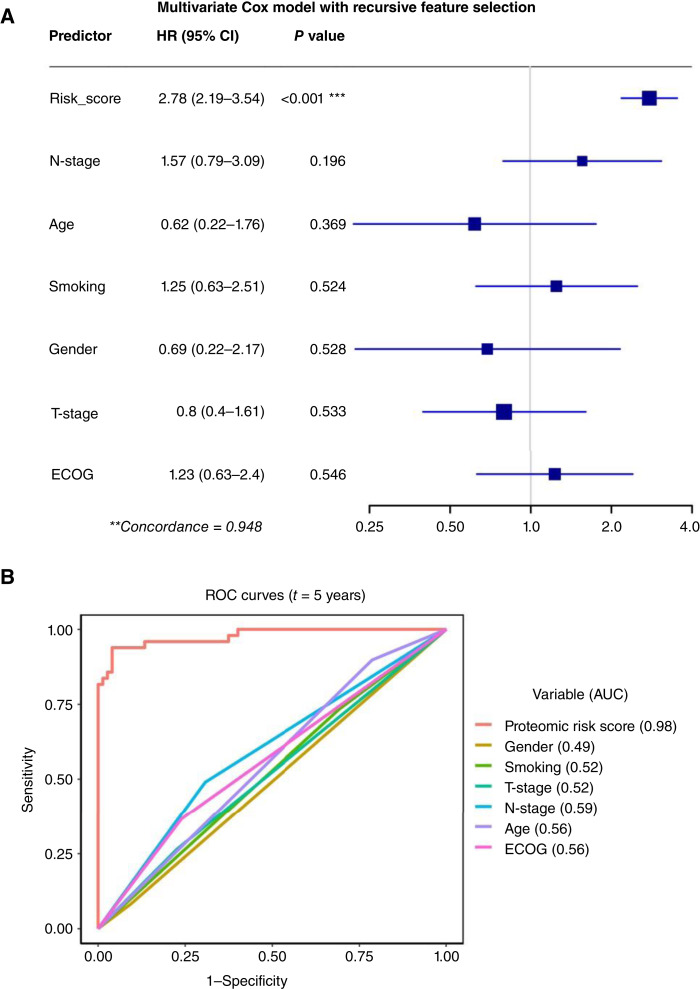
Multivariate Cox regression model and ROC curves incorporating the 26-peptide signature. **A,** Forest plot showing important clinicopathologic variables along with the risk signature and their association with disease-specific survival using a multivariate Cox regression model. Mean HRs are shown as squares, and whiskers represent 95% CI. **B,** ROC curve at 5 years after the date of treatment for the 26-peptide signature. The predicted AUC at 5 years is shown comparing the proteomic signature with six clinicopathologic variables.

The KM curves were plotted showing that the 26-peptide signature can stratify patients to low-, intermediate-, and high-risk groups for both RFS (Fig. 5A) and OS (Fig. 5B), containing 28 (23%), 60 (48%), and 36 (29%) patients, respectively. All patients assigned to the high-risk group experienced recurrence events. A multivariate Cox regression model incorporating the 26-peptide signature along with clinicopathologic variables proved to influence prognosis achieved an equivalent C-index (0.948) as the multivariate model using the 26-peptide signature alone. The HR and confidence interval (CI) comparison in the forest plot shows that the 26-peptide signature is the most significant predictor of RFS among all covariates (Fig. 6A). Comparing the AUCs of time-dependent ROC curves at 5 years (Fig. 6B) showed that the 26-peptide signature had a higher AUC (0.98) than the clinical covariates.

To assess the impact of unmatched clinical variables (T-stage, N-stage, RT dose, and systemic agent) on prognostic utility of the peptide signature, a further multivariate Cox regression analysis was performed (Supplementary Fig. S5A). Only the peptide risk score maintained a statistically significant association with RFS. An additional sensitivity analysis, in which seven patients treated with Cetuximab and <70 Gy RT were removed from the R cohort to improve intercohort balance for treatment variables, again demonstrated the peptide risk score as the only independent prognostic variable in the multivariate Cox regression model (Supplementary Fig. S5B). A subgroup analysis including only patients who received less than 70 Gy of RT (*n* = 15) showed that the risk score maintained its prognostic power (*P* < 0.002; Supplementary Fig. S6). Additionally, although the high-risk group identified by the peptide signature did have more patients treated with lower RT doses or no systemic therapy, there was no statistically significant difference between the high-risk and low-risk groups in terms of T-stage and N-stage (Table 2).

**Table 2:** Distribution of low- and high-risk peptide signature groups between categories of clinical variables

Variable	Total	Risk groups		*P* value
		Low	High	
T-stage				0.73
<4	93	42	51	
4	30	09	21	
N-stage				0.09
1	76	36	40	
>1	47	15	32	
ECOG				0.19
0	87	37	50	
>0	36	14	22	
RT dose				0.0019
70	108	48	60	
<7	15	03	12	
Chemotherapy				0.0004
Cisplatin.	81	39	42	
Others	42	12	30	
Total	123	51	72	

Abbreviation: ECOG, Eastern Cooperative Oncology Group.

## Discussion

To our knowledge, this study represents the most comprehensive proteomic analysis of HPV+OPSCC published to date. We focused on a clinically well-annotated 124-patient cohort with matched NATs and identified pathways associated with both malignancy and prognosis. In contrast to previous HPV+OPSCC proteomic studies, we adopted a peptide-only approach. Previous applications of proteomic methods to identify biomarkers for HPV+OPSCC have been limited. One study assessed DAProts between HPV-associated OPSCC, non-HPV–associated OPSCC, and normal oropharyngeal epithelium (37, 38). This furthered biological tumor insight but did not include a prognostic signature component. A DIA-MS analysis similar to that performed here was used to contrast disease-free HPV+OPSCC tumors with recurrent counterparts (38). From this, four proteins (HINT1, PFN1, RAD23B, and LDHB) were identified as potential prognostic markers (38). Feature selection in that study was based only on significantly downregulated proteins in recurrent tumors, but there was no comparison against clinicopathologic variables, there was heterogeneous treatment modality, and no signature was postulated. The four proteins were identified by analyzing 2,633 proteins from 53 tumor samples (38). In the current study which generated proteomic profile data from 124 patients and quantified 5,903 proteins, all four of these proteins were detected by DIA-MS, but none showed significant differential abundance.

Our data are consistent with and extend a recently published proteomic analysis of a similar cohort (21). Ho and colleagues compared 20 disease-free with 20 recurrent HPV+OPSCC samples using a similar DIA approach to that used here. They quantified 1,414 proteins, of which 77 were significantly DAProts, and of which two overlap with the study by Sepiashvili and colleagues (HINT1 and LDHB; ref. 38). Among the 77 DAProts, a different set of nine were identified in the current study as significant DAPeps (FABP5, PDCD4, ARPC4, WDR1, ANXA6, COTL1, RAC2, MYO1G, and EHD1), none of which were present in our signature.

The proteins in the 26-peptide signature (11 upregulated: LMNB1, DECR1, SPRR3, MMP2, ATP2A2, CNDP2, S100A4, LAP3, ALDOA, KRT17, and AGRN and 15 downregulated: ERP29, CDS2, ETHE1, STAG1, NOP56, ARL6IP5, GARS1, PSMB4, OPA3, MVP, GAPVD1, CTSZ, TCEA1, HLA-A, and WDR81) revealed no overlap with previously published HPV+OPSCC proteomic or genomic panels. Almost all proteins represented within the signature have been implicated in cancer progression. Many have been individually implicated in studies related to head and neck squamous cell carcinoma (HNSCC; particularly oral cavity subsites) but have not been reported in combination or as part of a unified prognostic signature.

For example, MMP2 and S100A4 were both upregulated in the prognostic peptide-only signature. MMP2 has long been known to be involved in tumor invasion and has been prognostically linked to many cancer types (39–41). Within oral cavity SCC (OCSCC) specifically, high MMP2 expression is recognized as an independent prognostic biomarker (42). It is expressed by fibroblasts within the microenvironment of perineural invasion, suggesting a potential role in the initiation of this unfavorable tumor event (43). S100A4 is an oncogene that increases cell motility and invasiveness by interacting with a nonmuscle myosin heavy chain (44). There is a correlation between overexpression of S100A4, lymph node involvement, distant metastases, and recurrence of disease in OCSCC (45). It already forms part of a prognostic model of six hypoxia-related genes in OCSCC (46). LMNB1 has been upregulated in several cancers including cervical, liver, prostate, and esophageal (47). Although not previously explored within HNSCC, LMNB1 is included within a panel of eight DNA damage repair genes associated with OS in bladder cancer (48).

KRT17 is involved in immune evasion and resistance and is part of a multiomic signature predicting survival in head and neck cancer (49–51). Elevated expression of KRT17 in OPSCC (HPV+/−) has been previously linked with decreased patient survival (52). Furthermore, its role in contributing to immune evasion has been highlighted in poorer response rates of HNSCC receiving immune checkpoint inhibitors (e.g., pembrolizumab; ref. 53). CNDP2 has not previously been reported within HPV+OPSCC but is highly expressed within ovarian cancer and indicates an overall poor prognosis (54). LAP3 (55), DECR1 (56), and SPRR3 (57) have all been linked to human esophageal SCC.

Among the downregulated proteins, ERP29 is an endoplasmic reticulum stress–inducible protein that may alter the prognosis of OPSCC through modulation by miR-4421 (58). *In vitro* studies using pharyngeal SCC cell lines demonstrated increased proliferation in cells silenced for ERP29 and exposed to cisplatin (59). This is supported by Carron and colleagues (58) who found shorter event-free survival in patients with OPSCC with reduced expression of ERP29. It is also proposed to be involved in radioresistance in nasopharyngeal carcinoma (60–62). CDS2, GAPVD1, and WDR81 have all previously been associated with poor prognosis in OCSCC when downregulated (63–65). All three proteins have also already been included within OCSCC gene prognostic signatures. In contrast, overexpression of MVP has been strongly related to poor disease-free survival in OCSCC (66). MVP has not previously explored within HPV+OPSCC and was downregulated within our 26-peptide risk signature.

KM curves of the categorized signature demonstrated favorable separation between different risk groups for both RFS and OS. Of note, and of particular significance when targeting suitable individuals for inclusion in future de-escalation trials, the entire high-risk group experienced a recurrence event, whereas none occurred in the low-risk group. Furthermore, multivariate analyses revealed that addition of clinicopathologic variables did not further improve prognostic power of the signature. Although patients with a previous smoking history (former >12 months ago) clustered more closely with the NR group than the R group, smoking history did not independently predict recurrence in our cohort.

In this study, we chose to perform a peptide-only analysis. The detected analytes in proteomics are peptides and not the proteins. Rolling up peptides to proteins (termed protein inference) produces a combined intensity value derived from multiple individual peptides, but there is information loss due to assumptions made to produce imputed intensity values for protein-level information (67, 68), and not all the peptides that map to a single protein demonstrate the same quantitative response (24, 68, 69). For example, intensity values for some individual peptides within an inferred protein are nonsignificantly different or even oppositely regulated. Such incoherent peptides can comprise from 1.6% to 11% of the peptides and distort computed intensity values (24, 69). Simple summarization of mean and median peptide intensity produces unreliable protein abundance estimates (70). In contrast to protein roll-up, consideration of peptide-only data more accurately represents the measured intensity quantitation and highlights the best performing peptides in terms of intensity and differential abundance. Additionally, statistical analysis is recognized as being more powerful at the peptide level (22–25).

Although the 26-peptide signature stratifies patient outcomes, it does not reflect all of the peptides that are most differentially abundant. Therefore, further mechanistic insights can be gleaned from the complete list of proteins. Moreover, potential drug targets could be identified. Among the 87 downregulated peptides (representing 52 unique proteins), five (PSB5, FINC, FIBB, FAS, and CO6A3) are targetable by FDA-approved drugs (71) and five (CAH9, AK1BA, MMP2, NAMPT, and TBB6) by investigational drugs (72), raising the possibility for further investigation of these proteins as potential drug targets to improve outcome for the R group of patients.

The functional role of proteins in the signatures was evaluated using pathway enrichment analysis. As expected, the 26-peptide signature in isolation showed no enrichment for proteins in a single pathway. Redundant interacting proteins in common pathways are expected to have similar predictive capacity and are filtered out at the machine learning stage. Instead, mechanistic insights were derived from the whole group of 405 DAPeps. Enrichment of the innate, adaptive, and IL-12 signaling pathways in the NR group was identified with this analysis. IL-12 is a well-established immunomodulatory cytokine that promotes host adaptive and innate immune activation against cancers (73). Although systemic IL-12 administration has resulted in unacceptable toxicity, intratumoral IL-12 immunotherapy has been studied in HPV+OPSCC in ongoing trials (74). The significance of the host immune response in tumor outcomes is well documented. Elevated CD8-positive T cells have a demonstrated survival benefit in HPV+OPSCC (75). Using tumor infiltrating lymphocyte as a surrogate marker, two studies have shown elevated tumor infiltrating lymphocytes can risk stratify HPV+OPSCC into subgroups for RFS and OS (76, 77).

There are inherent limitations to our study, the most salient being the lack of an independent validation cohort in which we can assess the predictive performance of this signature. Unfortunately, publicly accessible databases could not be utilized for this purpose because of insufficient numbers of HPV+OPSCC with matched outcome data. A further limitation is the more advanced nodal stage, lower RT dose, and higher rates of cetuximab received by the R group. Another limitation is a lack of balance that stems from the retrospective nature of the datasets. Additional sensitivity analysis does however support that the prognostic utility of the peptide signature is independent of these clinical variables. Reassuringly only the peptide signature was significantly associated with RFS in a multivariate Cox analysis, which included unmatched clinical variables. Additionally, it was highlighted as the only independent prognostic variable in a cetuximab/RT dose–adjusted subanalysis, supporting that it is independent of treatment differences. Experimentally, the decision to use p16 IHC as an indicator of HPV positivity is also a potential limitation that risks the inclusion of a potential 5% to 20% false positive rate within the study population (78). The analyses in the current study are based upon bulk tumor proteomics, and it is difficult to ascertain that the identified differences at the proteome level are based on tumor-related effects versus the host immune response.

In summary, we applied a DIA-MS approach to produce the most comprehensive proteomic analysis of HPV+OPSCC to date and develop the first prognostic protein signature for this cancer. The 26-peptide signature outperformed established clinicopathologic variables in subclassifying patients at risk of disease recurrence. Once validated, it is expected that clinical translation of the proposed model can provide guidance in individual treatment and also in recruitment of suitable low-risk patients for future de-escalation trials.

## Supplementary Material

Supplementary Data Methods used for (i) Tissue lysis and digestion (ii) Data-independent acquisition (iii) DIA-NN search parameters

Figure S1 Differential abundance 1,614 unique protein groups from 4,834 DAPeps discriminates tumor and NAT patient samples

Sequence Coverage Report Tables of all identified peptides for protein groups represented by the 26-peptide signature. The differentially abundant peptide (DAPep) that is part of the signature is highlighted in yellow.

Figure S2 Top 15 cellular functions and pathways for DAPeps in tumor vs to NAT samples

Figure S3 Distribution of the quantified peptides among the number of patient tumor samples.

Figure S4 Top 15 cellular functions and pathways for DAPep tumor samples from recurrence vs non-recurrence

Figure S5 Multivariate Cox regression models incorporating the 26-peptide signature

Figure S6 26-peptide signature stratifies HPV+ OPSCC patients into three risk groups for recurrence free survival (RFS)

Tables S1-S3 (Table S1) Differentially abundant peptides tumour vs NAT. (Table S2) Differentially abundant peptides recurrence vs non-recurrence. (Table S3) 26-peptide signature.
