# Supplementary material for: A Proteomic Signature for Human Papillomavirus–Associated Oropharyngeal Squamous Cell Carcinoma Predicts Patients at High Risk of Recurrence
Source: Cancer Res Commun. 2025 Apr 9;5(4):580–93. doi: 10.1158/2767-9764.CRC-23-0460 (PMC11979894; doi:10.1158/2767-9764.CRC-23-0460)
Supplement: Supplementary Data — Methods used for (i) Tissue lysis and digestion (ii) Data-independent acquisition (iii) DIA-NN search parameters [file crc-23-0460_supplementary_data_suppsd.docx]

# Supplementary Information

### Tissue lysis and digestion

Samples were processed in a one-pot approach in Barocycler MicroTubes (Pressure BioSciences, Inc., MD, USA). Samples were de-waxed using 120 µL heptane and 30 µL methanol and dried for 5 min in a GeneVac EZ-2 Plus Speed Vac Concentrator. 10.75 µL 5% sodium deoxycholate (SDC), 4.19 mM tris(2-carboxyethyl)phosphine (TCEP), 16.7 mM iodoacetamide (IOA) and 100 mM triethylammonium bicarbonate (TEAB) were added and tissues were heated to 90 °C for 7 min. Twelve x 1 mm Zirconium beads, 50 µL rapid digest buffer (Promega, WI, USA), 1 µL rapid trypsin/LysC (1 µg/µL) (Promega) and 1 µL of benzonase (1 µg/µL) (Sigma) were then added and the samples in MicroTubes were disrupted in a BeadBug beadbeater with shaking for 60 s at 3800 rpm to aid tissue lysis. After shaking, Pressure Cycling Technology (PCT) was used to augment the simultaneous in-tube lysis, reduction, alkylation and tryptic digestion of samples, with a Barocycler 2320EXT (Pressure BioSciences, Inc.) automated for 30 cycles of 50 s each at 45 kpsi, alternating 10 s ambient pressure (14.9 psi). A square pressure wave profile was selected, and external water bath applied for temperature control (56 °C). Sixteen samples were processed simultaneously in each Barocycler instrument, including a cell lysate of HEK293 cells used as a tryptic digestion control in each batch.

After digestion was completed, SDC was precipitated with 5 μL of 100% formic acid and samples centrifuged (18,000 xg, 15 min, 4°C), with the supernatant being diluted to 1 mL with 0.1% formic acid. Samples were desalted *via* Oasis Hydrophilic-Lipophilic Balance (HLB) reversed phase sorbent solid phase extraction (SPE) cartridges (1 cc, 30 mg, Waters, Rydalmere, NSW, Australia) and the eluents were dried and re-suspended in formic acid (0.1% v/v). The peptide yield was quantified by UV absorption at A280 nm in an Implen nanophotometer N60 (LabGear, Brisbane, QLD, Australia). Sample volume was normalized to a concentration of 0.5 µg/µL to give 2 µg MS load in 4 µL.

### Data-independent acquisition and proteomic data processing

Proteomic data was acquired by data-independent acquisition mass spectrometry (DIA-MS), across six SCIEX TripleTOF 6600 instruments interfaced with Ekspert NanoLC 425 (Eksigent) high performance liquid chromatography (HPLC) systems operating in microflow mode (**Figure 1b**). The peptide digests (2 µg) were spiked with retention time standards and injected onto a C18 trap column (SGE TRAPCOL C18 G203 300 µm x 100 mm) and desalted for 5 min at 10 µL/min with solvent A (0.1% [v/v] formic acid). The trap column was switched in-line with a reversed-phase capillary column (SGE C18 G203 250 mm × 300 µm ID 3 µm 200 Å), maintained at a temperature of 40 °C. The flow rate was 5 µL/min. The gradient started at 2% solvent B (99.9% [v/v] acetonitrile, 0.1% [v/v] formic acid) and increased to 10% over 5 min. This was followed by an increase of solvent B to 25% over 60 min, then a further increase to 40% for 5 min. The column was washed with a 4 min linear gradient to 95% solvent B held for 5 min, followed by a 9 min column equilibration step with 98% solvent A. The LC eluent was analysed using the Triple TOF 6600 system equipped with either a DuoSpray source or OptiFlow source and 50 µm internal diameter electrode and controlled by Analyst 1.8.1 software. The following parameters were used: 5500 V ion spray voltage; 25 nitrogen curtain gas; 100 °C TEM; 20 source gas 1; and 20 source gas 2. The 90 min run time consisted of a survey scan of 200 ms (TOF-MS) in the range 350–1250 m/z to collect the MS spectra. The top 40 precursor ions with charge states from +2 to +5 were selected for subsequent fragmentation, with an accumulation time of 50 ms per MS/MS experiment. The total cycle time was 2.3 s and MS/MS spectra were acquired in the range 100–2000 m/z.

For DIA acquisition SCIEX’s Variable Window Acquisition approach was used with 100 variable Q1 windows applied. Window width varied dependent on precursor analyte m/z density. For each window the ‘Q1 start m/z’, ‘Q1 stop m/z’ and collision energy spread (CES) was defined (Appendices 3.1). The 100 overlapping windows covered the precursor mass range 350 – 1250 m/z. 1.0 Da overlap was set between variable windows. Collision energy spread (CES) set-points were optimized for each window and ranged from 5 for smaller windows to 8 and then 10 with increasing window size. MS/MS spectra were collected from 100 – 2000 m/z. An accumulation time of 30 ms was used for each fragment ion scan in high resolution mode. A survey scan was performed at the start of the individual cycle giving an overall cycle time of 3.2 sec.

### DIA-NN search parameters and intensity quantification

Search parameters for DIA-NN 1.8 in the Linux environment were: Precursor ion generation using: deep learning-based spectra and RTs prediction, Protease Trypsin/P with 1 missed cleavage, N-terminus M excision and C carbamidomethylation and no variable mods; Peptide length range 7-30; Precursor m/z range 400-1250; Fragment ion m/z range 100-2000; 1% Precursor FDR with scan window, Mass and MS1 accuracy set at 0; Match-between-runs deactivated; neural networks, isotopologues, protein inference and interference removal were activated; and quantification using robust (high precision) LC. For filtering, only proteotypic peptides were retained at Global.Q.Value ≤ 0.01 and peptides with a raw intensity quantification of ≤15 were removed. Peptide data was log2 transformed, followed by median normalization using Normalyzer (79).

After roll-up following DIA-NN quantification protein quantitation was performed using MaxLFQ (label-free quantitation) which assumes a dominant population of proteins changes minimally between samples, estimates all the pair-wise peptide-protein comparisons and retains the absolute scale from the original summed-up peptide intensities. The default parameters were used and MaxLFQ was implemented using the DIANN R Package (<https://github.com/vdemichev/diann-rpackage>). Identified SiPPs were retained in the final data matrix when they were present in at least 20% of all tumor samples.
