# Supplementary material for: A Proteomic Signature for Human Papillomavirus–Associated Oropharyngeal Squamous Cell Carcinoma Predicts Patients at High Risk of Recurrence
Source: Cancer Res Commun. 2025 Apr 9;5(4):580–93. doi: 10.1158/2767-9764.CRC-23-0460 (PMC11979894; doi:10.1158/2767-9764.CRC-23-0460)
Supplement: Figure S1 — Differential abundance 1,614 unique protein groups from 4,834 DAPeps discriminates tumor and NAT patient samples [file crc-23-0460_figure_s1_suppsf1.pptx]

## Slide 1
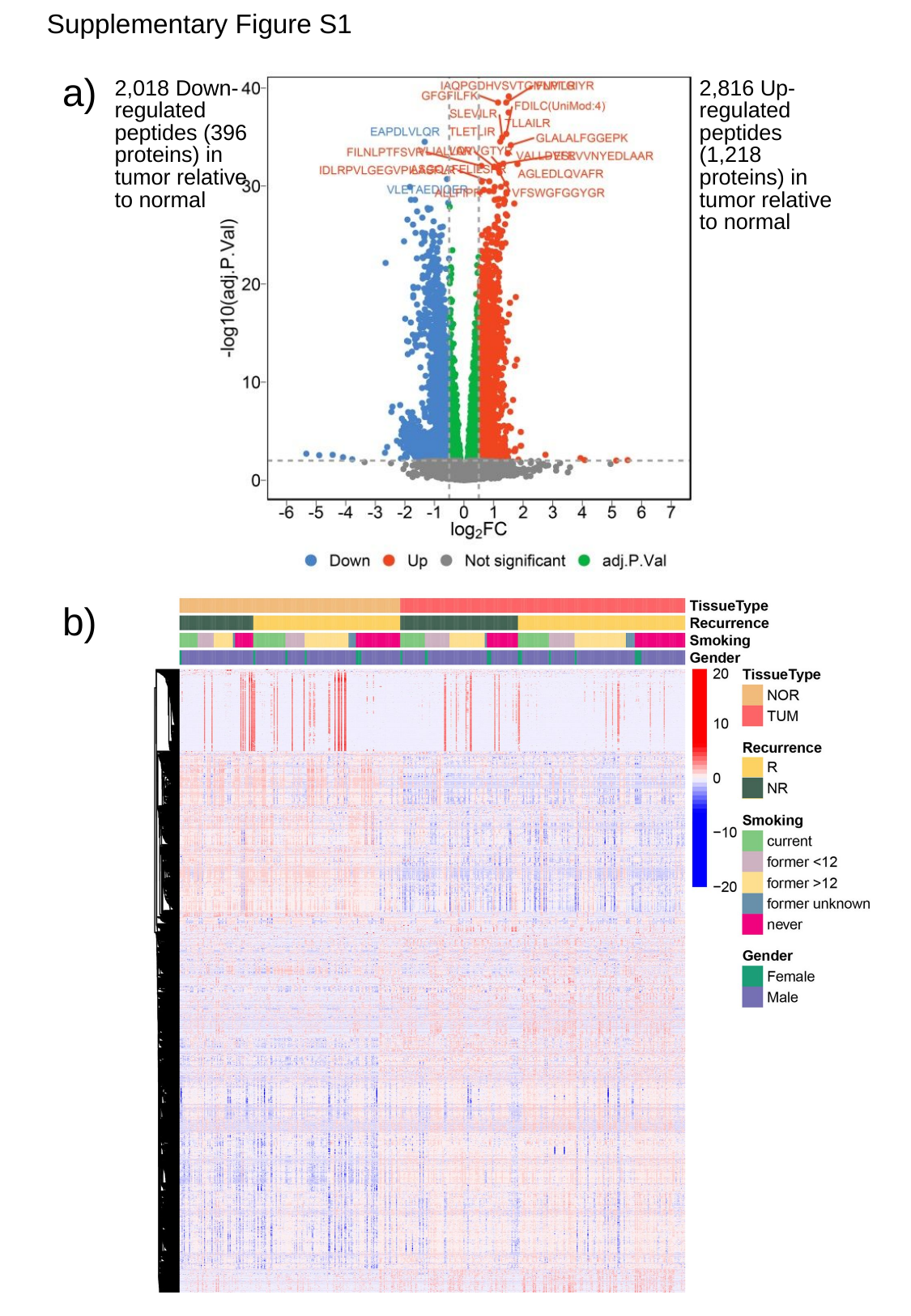

Supplementary Figure S1
a)
2,018 Down-regulated peptides (396 proteins) in tumor relative to normal
2,816 Up-regulated peptides (1,218 proteins) in tumor relative to normal
b)

## Slide 2
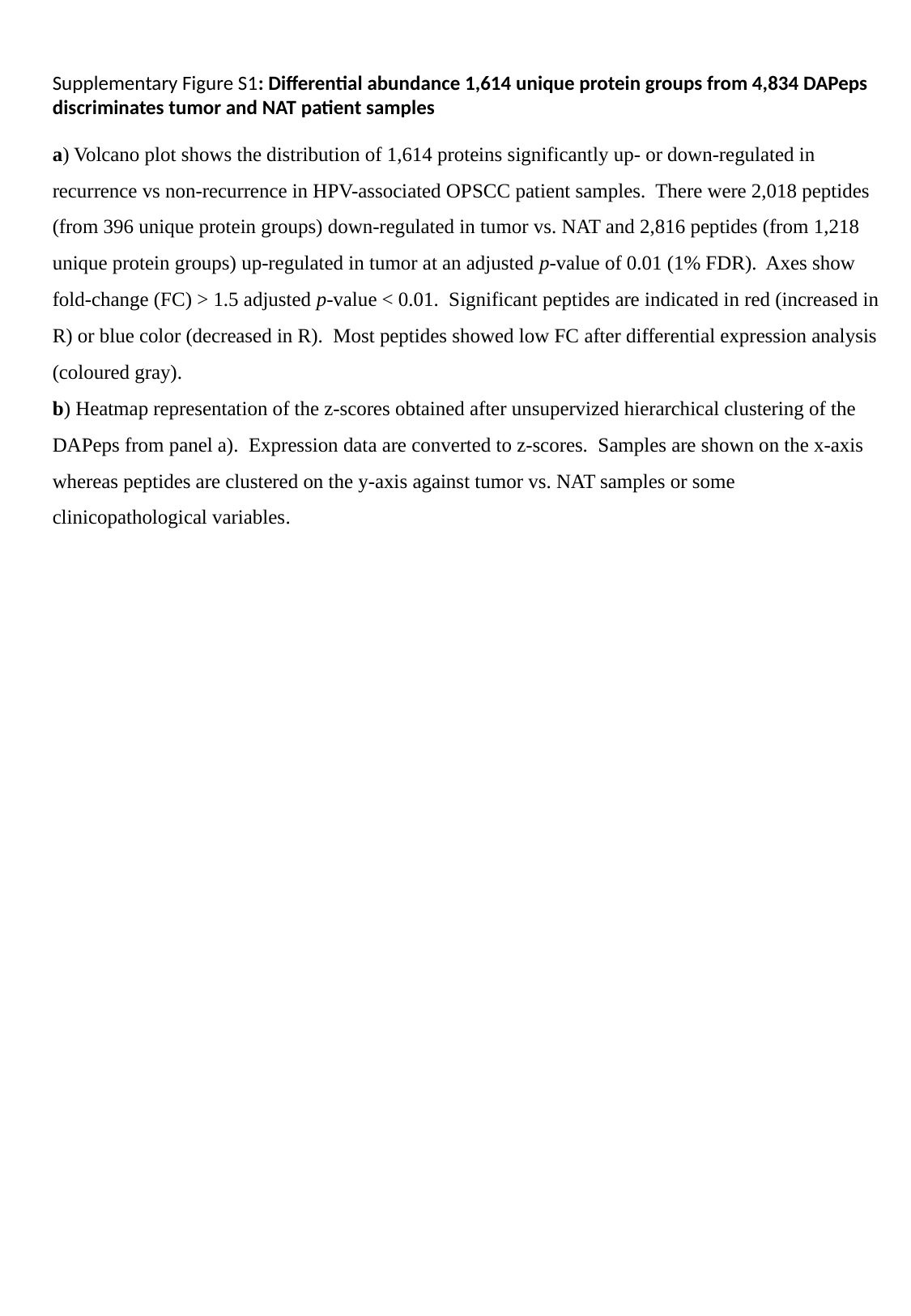

Supplementary Figure S1: Differential abundance 1,614 unique protein groups from 4,834 DAPeps discriminates tumor and NAT patient samples
a) Volcano plot shows the distribution of 1,614 proteins significantly up- or down-regulated in recurrence vs non-recurrence in HPV-associated OPSCC patient samples. There were 2,018 peptides (from 396 unique protein groups) down-regulated in tumor vs. NAT and 2,816 peptides (from 1,218 unique protein groups) up-regulated in tumor at an adjusted p-value of 0.01 (1% FDR). Axes show fold-change (FC) > 1.5 adjusted p-value < 0.01. Significant peptides are indicated in red (increased in R) or blue color (decreased in R). Most peptides showed low FC after differential expression analysis (coloured gray).
b) Heatmap representation of the z-scores obtained after unsupervized hierarchical clustering of the DAPeps from panel a). Expression data are converted to z-scores. Samples are shown on the x-axis whereas peptides are clustered on the y-axis against tumor vs. NAT samples or some clinicopathological variables.
