# Supplementary material for: A Proteomic Signature for Human Papillomavirus–Associated Oropharyngeal Squamous Cell Carcinoma Predicts Patients at High Risk of Recurrence
Source: Cancer Res Commun. 2025 Apr 9;5(4):580–93. doi: 10.1158/2767-9764.CRC-23-0460 (PMC11979894; doi:10.1158/2767-9764.CRC-23-0460)
Supplement: Sequence Coverage Report — Tables of all identified peptides for protein groups represented by the 26-peptide signature. The differentially abundant peptide (DAPep) that is part of the signature is highlighted in yellow. [file crc-23-0460_sequence_coverage_report_suppst.docx]

# 26-Peptide Signature - Protein sequence coverage report

Tables of all identified peptides for protein groups represented by the 26-peptide signature. The differentially abundant peptide (DAPep) that is part of the signature is highlighted in yellow.

The peptide matrix generated by DIA-NN software (Demichev *et al.*, 2020) was filtered using Python to contain only the proteins identified in the signatures. The survival signature matrix and the sequence database FASTA used to search the data were inputs for a Python script which generated a map of peptide sequence coverage of each protein. The parent protein sequence was obtained from the FASTA and the peptide coverage is shown by colouring the peptide within the protein sequence of the canonical sequence from the Uniprot database. This coverage represents peptides identified by DIA-MS for each protein across all samples in the cohort, i.e. whether a peptide was found in one or all samples. Alternating colours are used to differentiate sequential peptides in the same amino acid sequence to assist visual discrimination. The amino acid sequence coverage is reported as a percentage and number of amino acids. The output also lists the peptides with the peptide length, precursor mass and charge, the average peptide intensity detected by DIA-MS, the percent of samples runs where the peptide was not detected and whether the protein was up- or down-regulated. In the case of peptide-only signatures, the report also lists all other peptides from the same protein group that were identified in the DIA-MS data, but which were not necessarily significantly differentially abundant.

## ARL6IP5, PRA1 family protein 3 ([O75915, PRAF3_HUMAN](https://www.uniprot.org/uniprot/O75915))

Coverage: 20.2% (38/188)

| 1 MDVNIAPLRA WDDFFPGSDR FARPDFRDIS KWNNRVVSNL LYYQTNYLVV AAMMISIVGF LSPFNMILGG IVVVLVFTGF  81 VWAAHNKDVL RRMKKRYPTT FVMVVMLASY FLISMFGGVM VFVFGITFPL LLMFIHASLR LRNLKNKLEN KMEGIGLKRT  161 PMGIVLDALE QQEEGINRLT DYISKVKE |
| --- |

Peptides:

| No | Peptide | Peptide ID | Length | Mass | Charge | Intensity | Missing |
| --- | --- | --- | --- | --- | --- | --- | --- |
| 1 | AWDDFFPGSDR | ARL6IP5_10-20 | 11 | 1293.54 | 2 | 97.85 | 1.6% |
| 2 | RTPMGIVLDALEQQEEGINR | ARL6IP5_159-178 | 20 | 2250.14 | 3 | 18.81 | 23.4% |
| 3 | TPMGIVLDALEQQEEGINR | ARL6IP5_160-178 | 19 | 2094.04 | 2,3 | 25.70 | 0.8% |
| 4 | LTDYISK | ARL6IP5_179-185 | 7 | 820.43 | 2 | 70.32 | 0.3% |

## ATP2A2, Sarcoplasmic/endoplasmic reticulum calcium ATPase 2 ([P16615, AT2A2_HUMAN](https://www.uniprot.org/uniprot/P16615))

Coverage: 27.4% (286/1042)

| 1 MENAHTKTVE EVLGHFGVNE STGLSLEQVK KLKERWGSNE LPAEEGKTLL ELVIEQFEDL LVRILLLAAC ISFVLAWFEE  81 GEETITAFVE PFVILLILVA NAIVGVWQER NAENAIEALK EYEPEMGKVY RQDRKSVQRI KAKDIVPGDI VEIAVGDKVP  161 ADIRLTSIKS TTLRVDQSIL TGESVSVIKH TDPVPDPRAV NQDKKNMLFS GTNIAAGKAM GVVVATGVNT EIGKIRDEMV  241 ATEQERTPLQ QKLDEFGEQL SKVISLICIA VWIINIGHFN DPVHGGSWIR GAIYYFKIAV ALAVAAIPEG LPAVITTCLA  321 LGTRRMAKKN AIVRSLPSVE TLGCTSVICS DKTGTLTTNQ MSVCRMFILD RVEGDTCSLN EFTITGSTYA PIGEVHKDDK  401 PVNCHQYDGL VELATICALC NDSALDYNEA KGVYEKVGEA TETALTCLVE KMNVFDTELK GLSKIERANA CNSVIKQLMK  481 KEFTLEFSRD RKSMSVYCTP NKPSRTSMSK MFVKGAPEGV IDRCTHIRVG STKVPMTSGV KQKIMSVIRE WGSGSDTLRC  561 LALATHDNPL RREEMHLEDS ANFIKYETNL TFVGCVGMLD PPRIEVASSV KLCRQAGIRV IMITGDNKGT AVAICRRIGI  641 FGQDEDVTSK AFTGREFDEL NPSAQRDACL NARCFARVEP SHKSKIVEFL QSFDEITAMT GDGVNDAPAL KKAEIGIAMG  721 SGTAVAKTAS EMVLADDNFS TIVAAVEEGR AIYNNMKQFI RYLISSNVGE VVCIFLTAAL GFPEALIPVQ LLWVNLVTDG  801 LPATALGFNP PDLDIMNKPP RNPKEPLISG WLFFRYLAIG CYVGAATVGA AAWWFIAADG GPRVSFYQLS HFLQCKEDNP  881 DFEGVDCAIF ESPYPMTMAL SVLVTIEMCN ALNSLSENQS LLRMPPWENI WLVGSICLSM SLHFLILYVE PLPLIFQITP  961 LNVTQWLMVL KISLPVILMD ETLKFVARNY LEPGKECVQP ATKSCSFSAC TDGISWPFVL LIMPLVIWVY STDTNFSDMF  1041 WS |
| --- |

Peptides:

| No | Peptide | Peptide ID | Length | Mass | Charge | Intensity | Missing |
| --- | --- | --- | --- | --- | --- | --- | --- |
| 1 | TVEEVLGHFGVNESTGLSLEQVK | ATP2A2_8-30 | 23 | 2453.24 | 3 | 9.61 | 81.2% |
| 2 | DIVPGDIVEIAVGDK | ATP2A2_144-158 | 15 | 1520.81 | 2,3 | 9.19 | 28.7% |
| 3 | DIVPGDIVEIAVGDKVPADIR | ATP2A2_144-164 | 21 | 2172.18 | 2,3 | 15.85 | 36.7% |
| 4 | HTDPVPDPR | ATP2A2_190-198 | 9 | 1014.49 | 2 | 31.24 | 3.7% |
| 5 | AMGVVVATGVNTEIGK | ATP2A2_219-234 | 16 | 1526.81 | 2 | 6.15 | 50.3% |
| 6 | IRDEMVATEQER | ATP2A2_235-246 | 12 | 1457.69 | 2,3 | 18.29 | 14.8% |
| 7 | DEMVATEQER | ATP2A2_237-246 | 10 | 1188.51 | 2 | 5.37 | 86.7% |
| 8 | VEGDTCSLNEFTITGSTYAPIGEVHK | ATP2A2_372-397 | 26 | 2749.29 | 3 | 8.52 | 70.5% |
| 9 | MNVFDTELK | ATP2A2_452-460 | 9 | 1077.52 | 2 | 30.01 | 9.9% |
| 10 | ANACNSVIK | ATP2A2_468-476 | 9 | 900.45 | 2 | 18.73 | 31.5% |
| 11 | SMSVYCTPNKPSR | ATP2A2_493-505 | 13 | 1450.67 | 3 | 12.43 | 42.6% |
| 12 | VGSTKVPMTSGVK | ATP2A2_529-541 | 13 | 1271.69 | 3 | 16.26 | 88.6% |
| 13 | VPMTSGVK | ATP2A2_534-541 | 8 | 799.43 | 2 | 49.12 | 13.4% |
| 14 | EWGSGSDTLR | ATP2A2_550-559 | 10 | 1088.49 | 2 | 47.33 | 17% |
| 15 | CLALATHDNPLR | ATP2A2_560-571 | 12 | 1304.67 | 2,3 | 66.54 | 58% |
| 16 | CLALATHDNPLRR | ATP2A2_560-572 | 13 | 1460.77 | 3 | 16.57 | 17.1% |
| 17 | EEMHLEDSANFIK | ATP2A2_573-585 | 13 | 1543.70 | 2,3 | 7.97 | 38.8% |
| 18 | YETNLTFVGCVGMLDPPR | ATP2A2_586-603 | 18 | 1992.94 | 3 | 6.94 | 81.7% |
| 19 | IEVASSVK | ATP2A2_604-611 | 8 | 813.46 | 2 | 53.33 | 2.7% |
| 20 | RIGIFGQDEDVTSK | ATP2A2_637-650 | 14 | 1545.78 | 3 | 12.81 | 17.4% |
| 21 | IGIFGQDEDVTSK | ATP2A2_638-650 | 13 | 1389.68 | 2 | 40.00 | 0.4% |
| 22 | EFDELNPSAQR | ATP2A2_656-666 | 11 | 1286.59 | 2 | 79.16 | 0% |
| 23 | DACLNAR | ATP2A2_667-673 | 7 | 743.34 | 2 | 57.97 | 57.8% |
| 24 | IVEFLQSFDEITAMTGDGVNDAPALK | ATP2A2_686-711 | 26 | 2762.35 | 3 | 8.61 | 44% |
| 25 | NYLEPGKECVQPATK | ATP2A2_989-1003 | 15 | 1657.81 | 3 | 11.73 | 63.4% |

## HLA-A, HLA class I histocompatibility antigen, A alpha chain ([P04439, HLAA_HUMAN](https://www.uniprot.org/uniprot/P04439))

Coverage: 35.9% (131/365)

| 1 MAVMAPRTLL LLLSGALALT QTWAGSHSMR YFFTSVSRPG RGEPRFIAVG YVDDTQFVRF DSDAASQRME PRAPWIEQEG  81 PEYWDQETRN VKAQSQTDRV DLGTLRGYYN QSEAGSHTIQ IMYGCDVGSD GRFLRGYRQD AYDGKDYIAL NEDLRSWTAA  161 DMAAQITKRK WEAAHEAEQL RAYLDGTCVE WLRRYLENGK ETLQRTDPPK THMTHHPISD HEATLRCWAL GFYPAEITLT  241 WQRDGEDQTQ DTELVETRPA GDGTFQKWAA VVVPSGEEQR YTCHVQHEGL PKPLTLRWEL SSQPTIPIVG IIAGLVLLGA  321 VITGAVVAAV MWRRKSSDRK GGSYTQAASS DSAQGSDVSL TACKV |
| --- |

Peptides:

| No | Peptide | Peptide ID | Length | Mass | Charge | Intensity | Missing |
| --- | --- | --- | --- | --- | --- | --- | --- |
| 1 | YFFTSVSR | HLA-A_31-38 | 8 | 987.48 | 2 | 56.63 | 24.5% |
| 2 | YFFTSVSRPGR | HLA-A_31-41 | 11 | 1297.66 | 2,3 | 71.56 | 13.1% |
| 3 | FIAVGYVDDTQFVR | HLA-A_46-59 | 14 | 1610.81 | 2,3 | 373.78 | 0% |
| 4 | FDSDAASQR | HLA-A_60-68 | 9 | 977.42 | 2 | 236.48 | 3.4% |
| 5 | APWIEQEGPEYWDQETR | HLA-A_73-89 | 17 | 2114.93 | 2,3 | 34.14 | 44.4% |
| 6 | AQSQTDRVDLGTLR | HLA-A_93-106 | 14 | 1540.80 | 2,3 | 19.38 | 64.6% |
| 7 | QDAYDGKDYIALNEDLR | HLA-A_139-155 | 17 | 1979.92 | 3 | 12.39 | 69.6% |
| 8 | KWEAAHEAEQLR | HLA-A_170-181 | 12 | 1448.72 | 3 | 11.91 | 92% |
| 9 | WEAAHEAEQLR | HLA-A_171-181 | 11 | 1320.62 | 2,3 | 14.40 | 84.2% |
| 10 | AYLDGTCVEWLR | HLA-A_182-193 | 12 | 1406.67 | 2 | 10.48 | 86.8% |
| 11 | GGSYTQAASSDSAQGSDVSLTACKV | HLA-A_341-365 | 25 | 2371.06 | 3 | 1.87 | 98.3% |

## SPRR3, Small proline-rich protein 3 ([Q9UBC9, SPRR3_HUMAN](https://www.uniprot.org/uniprot/Q9UBC9))

Coverage: 80.5% (136/169)

| 1 MSSYQQKQTF TPPPQLQQQQ VKQPSQPPPQ EIFVPTTKEP CHSKVPQPGN TKIPEPGCTK VPEPGCTKVP EPGCTKVPEP  81 GCTKVPEPGC TKVPEPGCTK VPEPGYTKVP EPGSIKVPDQ GFIKFPEPGA IKVPEQGYTK VPVPGYTKLP EPCPSTVTPG  161 PAQQKTKQK |
| --- |

Peptides:

| No | Peptide | Peptide ID | Length | Mass | Charge | Intensity | Missing |
| --- | --- | --- | --- | --- | --- | --- | --- |
| 1 | QTFTPPPQLQQQQVK | SPRR3_8-22 | 15 | 1748.92 | 2,3 | 56.20 | 35.5% |
| 2 | QPSQPPPQEIFVPTTK | SPRR3_23-38 | 16 | 1774.93 | 2,3 | 50.11 | 36.9% |
| 3 | VPQPGNTK | SPRR3_45-52 | 8 | 821.44 | 2 | 232.18 | 54.6% |
| 4 | VPQPGNTKIPEPGCTK | SPRR3_45-60 | 16 | 1646.84 | 3 | 20.94 | 76.8% |
| 5 | IPEPGCTK | SPRR3_53-60 | 8 | 825.41 | 2 | 366.79 | 25.7% |
| 6 | IPEPGCTKVPEPGCTK | SPRR3_53-68 | 16 | 1636.80 | 3 | 16.07 | 85.1% |
| 7 | VPEPGCTK | SPRR3_61-68 | 8 | 811.39 | 2 | 1359.18 | 21.8% |
| 8 | VPEPGCTKVPEPGCTK | SPRR3_61-76 | 16 | 1622.78 | 3 | 28.11 | 61.9% |
| 9 | VPEPGCTKVPEPGYTK | SPRR3_93-108 | 16 | 1682.83 | 3 | 8.77 | 83.8% |
| 10 | VPEPGYTK | SPRR3_101-108 | 8 | 871.44 | 2 | 333.95 | 27.9% |
| 11 | VPEPGYTKVPEPGSIK | SPRR3_101-116 | 16 | 1678.89 | 3 | 10.75 | 76.5% |
| 12 | VPEPGSIK | SPRR3_109-116 | 8 | 807.45 | 2 | 507.75 | 24.4% |
| 13 | VPEPGSIKVPDQGFIK | SPRR3_109-124 | 16 | 1691.92 | 3 | 14.26 | 59.9% |
| 14 | VPDQGFIK | SPRR3_117-124 | 8 | 884.48 | 2 | 241.97 | 27.8% |
| 15 | VPDQGFIKFPEPGAIK | SPRR3_117-132 | 16 | 1723.93 | 3 | 20.10 | 54.3% |
| 16 | FPEPGAIK | SPRR3_125-132 | 8 | 839.45 | 2 | 214.20 | 27.5% |
| 17 | FPEPGAIKVPEQGYTK | SPRR3_125-140 | 16 | 1741.90 | 3 | 14.11 | 65.5% |
| 18 | VPEQGYTK | SPRR3_133-140 | 8 | 902.45 | 2 | 160.20 | 31.2% |
| 19 | VPVPGYTK | SPRR3_141-148 | 8 | 841.47 | 2 | 437.48 | 29.5% |
| 20 | VPVPGYTKLPEPCPSTVTPGPAQQK | SPRR3_141-165 | 25 | 2572.34 | 3,4 | 5.92 | 82.6% |
| 21 | LPEPCPSTVTPGPAQQK | SPRR3_149-165 | 17 | 1730.87 | 2,3 | 33.28 | 56.3% |

## PSMB4, Proteasome subunit beta type-4 ([P28070, PSB4_HUMAN](https://www.uniprot.org/uniprot/P28070))

Coverage: 50.4% (133/264)

| 1 MEAFLGSRSG LWAGGPAPGQ FYRIPSTPDS FMDPASALYR GPITRTQNPM VTGTSVLGVK FEGGVVIAAD MLGSYGSLAR  81 FRNISRIMRV NNSTMLGASG DYADFQYLKQ VLGQMVIDEE LLGDGHSYSP RAIHSWLTRA MYSRRSKMNP LWNTMVIGGY  161 ADGESFLGYV DMLGVAYEAP SLATGYGAYL AQPLLREVLE KQPVLSQTEA RDLVERCMRV LYYRDARSYN RFQIATVTEK  241 GVEIEGPLST ETNWDIAHMI SGFE |
| --- |

Peptides:

| No | Peptide | Peptide ID | Length | Mass | Charge | Intensity | Missing |
| --- | --- | --- | --- | --- | --- | --- | --- |
| 1 | TQNPMVTGTSVLGVK | PSMB4_46-60 | 15 | 1512.80 | 2 | 18.12 | 19.8% |
| 2 | FEGGVVIAADMLGSYGSLAR | PSMB4_61-80 | 20 | 1993.99 | 2,3 | 29.89 | 0.3% |
| 3 | VNNSTMLGASGDYADFQYLK | PSMB4_90-109 | 20 | 2174.99 | 2 | 10.67 | 51.7% |
| 4 | QVLGQMVIDEELLGDGHSYSPR | PSMB4_110-131 | 22 | 2424.17 | 3 | 25.64 | 38.3% |
| 5 | AIHSWLTR | PSMB4_132-139 | 8 | 964.52 | 2 | 51.56 | 28.1% |
| 6 | EVLEKQPVLSQTEAR | PSMB4_197-211 | 15 | 1707.92 | 3 | 8.55 | 62.3% |
| 7 | QPVLSQTEAR | PSMB4_202-211 | 10 | 1109.58 | 2 | 100.53 | 0% |
| 8 | FQIATVTEK | PSMB4_232-240 | 9 | 1017.55 | 2 | 140.83 | 5.7% |
| 9 | GVEIEGPLSTETNWDIAHMISGFE | PSMB4_241-264 | 24 | 2613.21 | 3 | 9.69 | 37.3% |

## CTSZ, Cathepsin Z ([Q9UBR2, CATZ_HUMAN](https://www.uniprot.org/uniprot/Q9UBR2))

Coverage: 15.8% (48/303)

| 1 MARRGPGWRP LLLLVLLAGA AQGGLYFRRG QTCYRPLRGD GLAPLGRSTY PRPHEYLSPA DLPKSWDWRN VDGVNYASIT  81 RNQHIPQYCG SCWAHASTSA MADRINIKRK GAWPSTLLSV QNVIDCGNAG SCEGGNDLSV WDYAHQHGIP DETCNNYQAK  161 DQECDKFNQC GTCNEFKECH AIRNYTLWRV GDYGSLSGRE KMMAEIYANG PISCGIMATE RLANYTGGIY AEYQDTTYIN  241 HVVSVAGWGI SDGTEYWIVR NSWGEPWGER GWLRIVTSTY KDGKGARYNL AIEEHCTFGD PIV |
| --- |

Peptides:

| No | Peptide | Peptide ID | Length | Mass | Charge | Intensity | Missing |
| --- | --- | --- | --- | --- | --- | --- | --- |
| 1 | NVDGVNYASITR | CTSZ_70-81 | 12 | 1289.64 | 2 | 125.57 | 6.1% |
| 2 | VGDYGSLSGR | CTSZ_190-199 | 10 | 991.47 | 2 | 125.53 | 9.9% |
| 3 | NSWGEPWGER | CTSZ_261-270 | 10 | 1198.52 | 2 | 83.54 | 16.7% |
| 4 | YNLAIEEHCTFGDPIV | CTSZ_288-303 | 16 | 1801.83 | 2 | 4.17 | 89.3% |

## LAP3, Cytosol aminopeptidase ([P28838, AMPL_HUMAN](https://www.uniprot.org/uniprot/P28838))

Coverage: 63.8% (331/519)

| 1 MFLLPLPAAG RVVVRRLAVR RFGSRSLSTA DMTKGLVLGI YSKEKEDDVP QFTSAGENFD KLLAGKLRET LNISGPPLKA  81 GKTRTFYGLH QDFPSVVLVG LGKKAAGIDE QENWHEGKEN IRAAVAAGCR QIQDLELSSV EVDPCGDAQA AAEGAVLGLY  161 EYDDLKQKKK MAVSAKLYGS GDQEAWQKGV LFASGQNLAR QLMETPANEM TPTRFAEIIE KNLKSASSKT EVHIRPKSWI  241 EEQAMGSFLS VAKGSDEPPV FLEIHYKGSP NANEPPLVFV GKGITFDSGG ISIKASANMD LMRADMGGAA TICSAIVSAA  321 KLNLPINIIG LAPLCENMPS GKANKPGDVV RAKNGKTIQV DNTDAEGRLI LADALCYAHT FNPKVILNAA TLTGAMDVAL  401 GSGATGVFTN SSWLWNKLFE ASIETGDRVW RMPLFEHYTR QVVDCQLADV NNIGKYRSAG ACTAAAFLKE FVTHPKWAHL  481 DIAGVMTNKD EVPYLRKGMT GRPTRTLIEF LLRFSQDNA |
| --- |

Peptides:

| No | Peptide | Peptide ID | Length | Mass | Charge | Intensity | Missing |
| --- | --- | --- | --- | --- | --- | --- | --- |
| 1 | GLVLGIYSK | LAP3_35-43 | 9 | 930.55 | 2 | 36.58 | 28.5% |
| 2 | EKEDDVPQFTSAGENFDK | LAP3_44-61 | 18 | 2036.90 | 3 | 8.78 | 48.6% |
| 3 | EDDVPQFTSAGENFDK | LAP3_46-61 | 16 | 1779.76 | 2 | 7.82 | 37.2% |
| 4 | LRETLNISGPPLK | LAP3_67-79 | 13 | 1418.82 | 3 | 22.73 | 16.4% |
| 5 | ETLNISGPPLK | LAP3_69-79 | 11 | 1149.64 | 2 | 47.96 | 0.3% |
| 6 | TFYGLHQDFPSVVLVGLGK | LAP3_85-103 | 19 | 2058.09 | 3 | 17.70 | 7.8% |
| 7 | AAGIDEQENWHEGK | LAP3_105-118 | 14 | 1564.69 | 3 | 3.63 | 69.6% |
| 8 | AAGIDEQENWHEGKENIR | LAP3_105-122 | 18 | 2076.96 | 4 | 4.45 | 75.5% |
| 9 | LYGSGDQEAWQK | LAP3_177-188 | 12 | 1362.62 | 2 | 12.84 | 42.6% |
| 10 | GVLFASGQNLAR | LAP3_189-200 | 12 | 1213.66 | 2 | 234.47 | 0% |
| 11 | QLMETPANEMTPTR | LAP3_201-214 | 14 | 1599.74 | 2 | 61.95 | 1% |
| 12 | FAEIIEK | LAP3_215-221 | 7 | 830.45 | 2 | 117.11 | 2.6% |
| 13 | TEVHIRPK | LAP3_230-237 | 8 | 960.55 | 2 | 1.90 | 85.2% |
| 14 | SWIEEQAMGSFLSVAK | LAP3_238-253 | 16 | 1763.86 | 2,3 | 10.86 | 21.5% |
| 15 | GSDEPPVFLEIHYK | LAP3_254-267 | 14 | 1611.79 | 2,3 | 23.84 | 2.3% |
| 16 | GSPNANEPPLVFVGK | LAP3_268-282 | 15 | 1506.78 | 2,3 | 30.71 | 2.6% |
| 17 | GITFDSGGISIK | LAP3_283-294 | 12 | 1175.62 | 2 | 79.25 | 0.4% |
| 18 | ASANMDLMR | LAP3_295-303 | 9 | 989.44 | 2 | 42.08 | 1.2% |
| 19 | ADMGGAATICSAIVSAAK | LAP3_304-321 | 18 | 1617.79 | 2,3 | 12.06 | 21.9% |
| 20 | LNLPINIIGLAPLCENMPSGK | LAP3_322-342 | 21 | 2188.17 | 2,3 | 11.86 | 26.1% |
| 21 | TIQVDNTDAEGR | LAP3_357-368 | 12 | 1299.61 | 2 | 35.82 | 0.7% |
| 22 | LILADALCYAHTFNPK | LAP3_369-384 | 16 | 1770.91 | 2,3 | 21.27 | 8.2% |
| 23 | LFEASIETGDR | LAP3_418-428 | 11 | 1218.59 | 2 | 38.36 | 3.5% |
| 24 | MPLFEHYTR | LAP3_432-440 | 9 | 1174.56 | 2 | 13.99 | 62.4% |
| 25 | QVVDCQLADVNNIGK | LAP3_441-455 | 15 | 1596.79 | 2 | 7.56 | 39.3% |
| 26 | SAGACTAAAFLK | LAP3_458-469 | 12 | 1091.54 | 2 | 29.27 | 8.1% |
| 27 | WAHLDIAGVMTNK | LAP3_477-489 | 13 | 1436.72 | 3 | 11.31 | 68% |
| 28 | TLIEFLLR | LAP3_506-513 | 8 | 985.60 | 2 | 362.76 | 0% |

## GARS1, Glycine--tRNA ligase ([P41250, GARS_HUMAN](https://www.uniprot.org/uniprot/P41250))

Coverage: 39.5% (292/739)

| 1 MPSPRPVLLR GARAALLLLL PPRLLARPSL LLRRSLSAAS CPPISLPAAA SRSSMDGAGA EEVLAPLRLA VRQQGDLVRK  81 LKEDKAPQVD VDKAVAELKA RKRVLEAKEL ALQPKDDIVD RAKMEDTLKR RFFYDQAFAI YGGVSGLYDF GPVGCALKNN  161 IIQTWRQHFI QEEQILEIDC TMLTPEPVLK TSGHVDKFAD FMVKDVKNGE CFRADHLLKA HLQKLMSDKK CSVEKKSEME  241 SVLAQLDNYG QQELADLFVN YNVKSPITGN DLSPPVSFNL MFKTFIGPGG NMPGYLRPET AQGIFLNFKR LLEFNQGKLP  321 FAAAQIGNSF RNEISPRSGL IRVREFTMAE IEHFVDPSEK DHPKFQNVAD LHLYLYSAKA QVSGQSARKM RLGDAVEQGV  401 INNTVLGYFI GRIYLYLTKV GISPDKLRFR QHMENEMAHY ACDCWDAESK TSYGWIEIVG CADRSCYDLS CHARATKVPL  481 VAEKPLKEPK TVNVVQFEPS KGAIGKAYKK DAKLVMEYLA ICDECYITEM EMLLNEKGEF TIETEGKTFQ LTKDMINVKR  561 FQKTLYVEEV VPNVIEPSFG LGRIMYTVFE HTFHVREGDE QRTFFSFPAV VAPFKCSVLP LSQNQEFMPF VKELSEALTR  641 HGVSHKVDDS SGSIGRRYAR TDEIGVAFGV TIDFDTVNKT PHTATLRDRD SMRQIRAEIS ELPSIVQDLA NGNITWADVE  721 ARYPLFEGQE TGKKETIEE |
| --- |

Peptides:

| No | Peptide | Peptide ID | Length | Mass | Charge | Intensity | Missing |
| --- | --- | --- | --- | --- | --- | --- | --- |
| 1 | SSMDGAGAEEVLAPLR | GARS1_53-68 | 16 | 1583.76 | 2 | 4.81 | 78.5% |
| 2 | NNIIQTWR | GARS1_159-166 | 8 | 1025.54 | 2 | 48.32 | 2% |
| 3 | QHFIQEEQILEIDCTMLTPEPVLK | GARS1_167-190 | 24 | 2835.42 | 3 | 9.38 | 47.9% |
| 4 | FADFMVK | GARS1_198-204 | 7 | 838.40 | 2 | 31.87 | 26% |
| 5 | LLEFNQGK | GARS1_311-318 | 8 | 929.50 | 2 | 35.01 | 11.7% |
| 6 | LPFAAAQIGNSFR | GARS1_319-331 | 13 | 1372.73 | 2,3 | 24.20 | 12.9% |
| 7 | EFTMAEIEHFVDPSEK | GARS1_345-360 | 16 | 1889.85 | 3 | 17.13 | 35.4% |
| 8 | LGDAVEQGVINNTVLGYFIGR | GARS1_392-412 | 21 | 2216.16 | 2,3 | 24.53 | 34.7% |
| 9 | IYLYLTK | GARS1_413-419 | 7 | 894.52 | 2 | 28.16 | 78.9% |
| 10 | TSYGWIEIVGCADR | GARS1_451-464 | 14 | 1550.72 | 2 | 14.41 | 39.4% |
| 11 | SCYDLSCHAR | GARS1_465-474 | 10 | 1135.45 | 2,3 | 12.48 | 15.1% |
| 12 | TVNVVQFEPSK | GARS1_491-501 | 11 | 1228.65 | 2 | 32.51 | 3.5% |
| 13 | GEFTIETEGK | GARS1_538-547 | 10 | 1091.51 | 2 | 24.19 | 6.5% |
| 14 | TLYVEEVVPNVIEPSFGLGR | GARS1_564-583 | 20 | 2199.16 | 2,3 | 35.18 | 0.5% |
| 15 | IMYTVFEHTFHVR | GARS1_584-596 | 13 | 1660.82 | 3,4 | 11.74 | 5.2% |
| 16 | TFFSFPAVVAPFK | GARS1_603-615 | 13 | 1438.76 | 2 | 52.20 | 0.5% |
| 17 | CSVLPLSQNQEFMPFVK | GARS1_616-632 | 17 | 1947.96 | 2 | 22.11 | 47.9% |
| 18 | ELSEALTR | GARS1_633-640 | 8 | 899.47 | 2 | 60.33 | 1.3% |
| 19 | VDDSSGSIGR | GARS1_647-656 | 10 | 973.45 | 2 | 49.67 | 0% |
| 20 | TPHTATLR | GARS1_680-687 | 8 | 877.48 | 2 | 9.35 | 54.4% |
| 21 | AEISELPSIVQDLANGNITWADVEAR | GARS1_697-722 | 26 | 2792.40 | 3,4 | 18.10 | 9.8% |
| 22 | YPLFEGQETGK | GARS1_723-733 | 11 | 1249.60 | 2 | 35.05 | 1.4% |
| 23 | YPLFEGQETGKK | GARS1_723-734 | 12 | 1377.69 | 3 | 5.54 | 64.6% |

## LMNB1, Lamin-B1 ([P20700, LMNB1_HUMAN](https://www.uniprot.org/uniprot/P20700))

Coverage: 61.8% (362/586)

| 1 MATATPVPPR MGSRAGGPTT PLSPTRLSRL QEKEELRELN DRLAVYIDKV RSLETENSAL QLQVTEREEV RGRELTGLKA  81 LYETELADAR RALDDTARER AKLQIELGKC KAEHDQLLLN YAKKESDLNG AQIKLREYEA ALNSKDAALA TALGDKKSLE  161 GDLEDLKDQI AQLEASLAAA KKQLADETLL KVDLENRCQS LTEDLEFRKS MYEEEINETR RKHETRLVEV DSGRQIEYEY  241 KLAQALHEMR EQHDAQVRLY KEELEQTYHA KLENARLSSE MNTSTVNSAR EELMESRMRI ESLSSQLSNL QKESRACLER  321 IQELEDLLAK EKDNSRRMLT DKEREMAEIR DQMQQQLNDY EQLLDVKLAL DMEISAYRKL LEGEEERLKL SPSPSSRVTV  401 SRASSSRSVR TTRGKRKRVD VEESEASSSV SISHSASATG NVCIEEIDVD GKFIRLKNTS EQDQPMGGWE MIRKIGDTSV  481 SYKYTSRYVL KAGQTVTIWA ANAGVTASPP TDLIWKNQNS WGTGEDVKVI LKNSQGEEVA QRSTVFKTTI PEEEEEEEEA  561 AGVVVEEELF HQQGTPRASN RSCAIM |
| --- |

Peptides:

| No | Peptide | Peptide ID | Length | Mass | Charge | Intensity | Missing |
| --- | --- | --- | --- | --- | --- | --- | --- |
| 1 | AGGPTTPLSPTR | LMNB1_15-26 | 12 | 1135.60 | 2 | 415.42 | 0% |
| 2 | LAVYIDK | LMNB1_43-49 | 7 | 802.46 | 2 | 195.81 | 0.1% |
| 3 | SLETENSALQLQVTER | LMNB1_52-67 | 16 | 1798.91 | 2,3 | 39.10 | 0.1% |
| 4 | ELTGLKALYETELADAR | LMNB1_74-90 | 17 | 1873.98 | 3 | 5.15 | 92.6% |
| 5 | ALYETELADAR | LMNB1_80-90 | 11 | 1232.60 | 2,3 | 179.15 | 0% |
| 6 | ALYETELADARR | LMNB1_80-91 | 12 | 1388.70 | 3 | 30.17 | 38.8% |
| 7 | RALDDTAR | LMNB1_91-98 | 8 | 898.46 | 2 | 15.60 | 56.3% |
| 8 | LQIELGK | LMNB1_103-109 | 7 | 781.47 | 2 | 78.94 | 15.7% |
| 9 | AEHDQLLLNYAK | LMNB1_112-123 | 12 | 1395.71 | 3 | 82.09 | 35.4% |
| 10 | ESDLNGAQIK | LMNB1_125-134 | 10 | 1055.52 | 2 | 10.26 | 77.3% |
| 11 | LREYEAALNSK | LMNB1_135-145 | 11 | 1274.66 | 2,3 | 24.55 | 21.5% |
| 12 | EYEAALNSK | LMNB1_137-145 | 9 | 1005.48 | 2 | 28.94 | 2.2% |
| 13 | DAALATALGDK | LMNB1_146-156 | 11 | 1026.53 | 2 | 53.92 | 12.8% |
| 14 | DAALATALGDKK | LMNB1_146-157 | 12 | 1154.63 | 2 | 34.52 | 16.3% |
| 15 | QLADETLLK | LMNB1_183-191 | 9 | 1011.56 | 2 | 31.82 | 14.1% |
| 16 | CQSLTEDLEFR | LMNB1_198-208 | 11 | 1321.60 | 2 | 98.39 | 0.9% |
| 17 | CQSLTEDLEFRK | LMNB1_198-209 | 12 | 1449.69 | 3 | 17.30 | 54.4% |
| 18 | KSMYEEEINETR | LMNB1_209-220 | 12 | 1509.68 | 2,3 | 6.88 | 71.3% |
| 19 | SMYEEEINETR | LMNB1_210-220 | 11 | 1381.58 | 2 | 52.98 | 2.1% |
| 20 | SMYEEEINETRR | LMNB1_210-221 | 12 | 1537.68 | 3 | 13.33 | 69.1% |
| 21 | LVEVDSGR | LMNB1_227-234 | 8 | 855.45 | 2 | 424.51 | 1.4% |
| 22 | QIEYEYK | LMNB1_235-241 | 7 | 953.45 | 2 | 31.18 | 12% |
| 23 | LAQALHEMR | LMNB1_242-250 | 9 | 1049.54 | 2 | 16.85 | 15% |
| 24 | LYKEELEQTYHAK | LMNB1_259-271 | 13 | 1632.81 | 3,4 | 5.06 | 90.7% |
| 25 | EELEQTYHAK | LMNB1_262-271 | 10 | 1228.57 | 2,3 | 14.28 | 36.9% |
| 26 | LSSEMNTSTVNSAR | LMNB1_277-290 | 14 | 1477.68 | 2 | 112.32 | 0% |
| 27 | EELMESR | LMNB1_291-297 | 7 | 874.39 | 2 | 262.32 | 0% |
| 28 | MRIESLSSQLSNLQK | LMNB1_298-312 | 15 | 1714.90 | 3 | 28.66 | 24.3% |
| 29 | IESLSSQLSNLQK | LMNB1_300-312 | 13 | 1427.76 | 2,3 | 45.44 | 0.7% |
| 30 | IQELEDLLAK | LMNB1_321-330 | 10 | 1152.64 | 2 | 166.97 | 0% |
| 31 | DQMQQQLNDYEQLLDVK | LMNB1_351-367 | 17 | 2088.98 | 3 | 6.87 | 68.5% |
| 32 | LALDMEISAYR | LMNB1_368-378 | 11 | 1262.63 | 2 | 57.37 | 0.3% |
| 33 | LALDMEISAYRK | LMNB1_368-379 | 12 | 1390.73 | 3 | 12.48 | 37.1% |
| 34 | LKNTSEQDQPMGGWEMIR | LMNB1_456-473 | 18 | 2100.97 | 3 | 10.78 | 86.3% |
| 35 | NTSEQDQPMGGWEMIR | LMNB1_458-473 | 16 | 1859.79 | 2,3 | 19.20 | 11.1% |
| 36 | KIGDTSVSYK | LMNB1_474-483 | 10 | 1078.57 | 2 | 10.53 | 55.7% |
| 37 | IGDTSVSYK | LMNB1_475-483 | 9 | 950.47 | 2 | 47.64 | 6.8% |
| 38 | AGQTVTIWAANAGVTASPPTDLIWK | LMNB1_492-516 | 25 | 2549.33 | 3 | 15.55 | 66.4% |
| 39 | NQNSWGTGEDVK | LMNB1_517-528 | 12 | 1315.58 | 2 | 16.98 | 27.5% |
| 40 | NSQGEEVAQR | LMNB1_533-542 | 10 | 1098.51 | 2 | 40.31 | 5.7% |
| 41 | TTIPEEEEEEEEAAGVVVEEELFHQQGTPR | LMNB1_548-577 | 30 | 3392.55 | 3,4 | 25.12 | 11.2% |

## CNDP2, Cytosolic non-specific dipeptidase ([Q96KP4, CNDP2_HUMAN](https://www.uniprot.org/uniprot/Q96KP4))

Coverage: 56.4% (268/475)

| 1 MAALTTLFKY IDENQDRYIK KLAKWVAIQS VSAWPEKRGE IRRMMEVAAA DVKQLGGSVE LVDIGKQKLP DGSEIPLPPI  81 LLGRLGSDPQ KKTVCIYGHL DVQPAALEDG WDSEPFTLVE RDGKLYGRGS TDDKGPVAGW INALEAYQKT GQEIPVNVRF  161 CLEGMEESGS EGLDELIFAR KDTFFKDVDY VCISDNYWLG KKKPCITYGL RGICYFFIEV ECSNKDLHSG VYGGSVHEAM  241 TDLILLMGSL VDKRGNILIP GINEAVAAVT EEEHKLYDDI DFDIEEFAKD VGAQILLHSH KKDILMHRWR YPSLSLHGIE  321 GAFSGSGAKT VIPRKVVGKF SIRLVPNMTP EVVGEQVTSY LTKKFAELRS PNEFKVYMGH GGKPWVSDFS HPHYLAGRRA  401 MKTVFGVEPD LTREGGSIPV TLTFQEATGK NVMLLPVGSA DDGAHSQNEK LNRYNYIEGT KMLAAYLYEV SQLKD |
| --- |

Peptides:

| No | Peptide | Peptide ID | Length | Mass | Charge | Intensity | Missing |
| --- | --- | --- | --- | --- | --- | --- | --- |
| 1 | YIDENQDR | CNDP2_10-17 | 8 | 1033.45 | 2 | 14.25 | 64.2% |
| 2 | MMEVAAADVK | CNDP2_44-53 | 10 | 1045.49 | 2 | 28.03 | 2.3% |
| 3 | QLGGSVELVDIGK | CNDP2_54-66 | 13 | 1295.71 | 2 | 19.74 | 9.9% |
| 4 | QKLPDGSEIPLPPILLGR | CNDP2_67-84 | 18 | 1924.11 | 2,3 | 24.37 | 0.5% |
| 5 | LPDGSEIPLPPILLGR | CNDP2_69-84 | 16 | 1667.96 | 2,3 | 75.21 | 0.5% |
| 6 | TVCIYGHLDVQPAALEDGWDSEPFTLVER | CNDP2_93-121 | 29 | 3241.54 | 3 | 21.29 | 87.9% |
| 7 | GSTDDKGPVAGWINALEAYQK | CNDP2_129-149 | 21 | 2201.08 | 3 | 14.42 | 18.4% |
| 8 | TGQEIPVNVR | CNDP2_150-159 | 10 | 1093.59 | 2 | 178.20 | 0% |
| 9 | FCLEGMEESGSEGLDELIFAR | CNDP2_160-180 | 21 | 2313.03 | 2,3 | 19.43 | 11.7% |
| 10 | RGNILIPGINEAVAAVTEEEHK | CNDP2_254-275 | 22 | 2341.24 | 4 | 4.95 | 77.3% |
| 11 | GNILIPGINEAVAAVTEEEHK | CNDP2_255-275 | 21 | 2185.14 | 2,3 | 37.08 | 0.5% |
| 12 | LYDDIDFDIEEFAK | CNDP2_276-289 | 14 | 1713.78 | 2 | 10.96 | 18.5% |
| 13 | DVGAQILLHSHK | CNDP2_290-301 | 12 | 1298.71 | 3 | 10.40 | 34.1% |
| 14 | LVPNMTPEVVGEQVTSYLTK | CNDP2_344-363 | 20 | 2186.13 | 2,3 | 14.18 | 16.7% |
| 15 | TVFGVEPDLTR | CNDP2_403-413 | 11 | 1214.63 | 2 | 84.71 | 0% |
| 16 | EGGSIPVTLTFQEATGK | CNDP2_414-430 | 17 | 1715.87 | 2,3 | 31.41 | 0.7% |
| 17 | NVMLLPVGSADDGAHSQNEK | CNDP2_431-450 | 20 | 2062.97 | 3 | 9.27 | 58.2% |
| 18 | YNYIEGTK | CNDP2_454-461 | 8 | 968.46 | 2 | 31.28 | 7.2% |
| 19 | MLAAYLYEVSQLKD | CNDP2_462-475 | 14 | 1624.82 | 2,3 | 6.07 | 38.8% |

## TCEA1, Transcription elongation factor A protein 1 ([P23193, TCEA1_HUMAN](https://www.uniprot.org/uniprot/P23193))

Coverage: 46.5% (140/301)

| 1 MEDEVVRFAK KMDKMVQKKN AAGALDLLKE LKNIPMTLEL LQSTRIGMSV NAIRKQSTDE EVTSLAKSLI KSWKKLLDGP  81 STEKDLDEKK KEPAITSQNS PEAREESTSS GNVSNRKDET NARDTYVSSF PRAPSTSDSV RLKCREMLAA ALRTGDDYIA  161 IGADEEELGS QIEEAIYQEI RNTDMKYKNR VRSRISNLKD AKNPNLRKNV LCGNIPPDLF ARMTAEEMAS DELKEMRKNL  241 TKEAIREHQM AKTGGTQTDL FTCGKCKKKN CTYTQVQTRS ADEPMTTFVV CNECGNRWKF C |
| --- |

Peptides:

| No | Peptide | Peptide ID | Length | Mass | Charge | Intensity | Missing |
| --- | --- | --- | --- | --- | --- | --- | --- |
| 1 | NAAGALDLLK | TCEA1_20-29 | 10 | 966.55 | 2 | 39.33 | 53.9% |
| 2 | NIPMTLELLQSTR | TCEA1_33-45 | 13 | 1496.80 | 2 | 19.41 | 35.2% |
| 3 | IGMSVNAIR | TCEA1_46-54 | 9 | 941.51 | 2 | 33.05 | 8.4% |
| 4 | QSTDEEVTSLAK | TCEA1_56-67 | 12 | 1288.61 | 2 | 7.51 | 68.4% |
| 5 | EESTSSGNVSNR | TCEA1_105-116 | 12 | 1247.54 | 2 | 4.19 | 56.5% |
| 6 | DTYVSSFPR | TCEA1_124-132 | 9 | 1052.49 | 2 | 55.88 | 0.3% |
| 7 | APSTSDSVR | TCEA1_133-141 | 9 | 900.43 | 2 | 36.67 | 19.6% |
| 8 | EMLAAALR | TCEA1_146-153 | 8 | 855.46 | 2 | 115.34 | 0.1% |
| 9 | TGDDYIAIGADEEELGSQIEEAIYQEIR | TCEA1_154-181 | 28 | 3108.44 | 3 | 8.26 | 85.8% |
| 10 | MTAEEMASDELK | TCEA1_223-234 | 12 | 1335.57 | 2 | 8.51 | 61% |
| 11 | SADEPMTTFVVCNECGNR | TCEA1_280-297 | 18 | 1953.80 | 3 | 6.20 | 77.4% |

## S100A4, Protein S100-A4 ([P26447, S10A4_HUMAN](https://www.uniprot.org/uniprot/P26447))

Coverage: 36.6% (37/101)

| 1 MACPLEKALD VMVSTFHKYS GKEGDKFKLN KSELKELLTR ELPSFLGKRT DEAAFQKLMS NLDSNRDNEV DFQEYCVFLS  81 CIAMMCNEFF EGFPDKQPRK K |
| --- |

Peptides:

| No | Peptide | Peptide ID | Length | Mass | Charge | Intensity | Missing |
| --- | --- | --- | --- | --- | --- | --- | --- |
| 1 | ALDVMVSTFHK | S100A4_8-18 | 11 | 1228.63 | 2,3 | 38.20 | 16.3% |
| 2 | ELPSFLGK | S100A4_41-48 | 8 | 871.48 | 2 | 282.09 | 16.1% |
| 3 | RTDEAAFQK | S100A4_49-57 | 9 | 1046.51 | 2 | 15.13 | 27.7% |
| 4 | TDEAAFQK | S100A4_50-57 | 8 | 890.41 | 2 | 41.83 | 17% |
| 5 | LMSNLDSNR | S100A4_58-66 | 9 | 1030.49 | 2 | 40.85 | 24.7% |

## ETHE1, Persulfide dioxygenase ETHE1, mitochondrial ([O95571, ETHE1_HUMAN](https://www.uniprot.org/uniprot/O95571))

Coverage: 69.3% (176/254)

| 1 MAEAVLRVAR RQLSQRGGSG APILLRQMFE PVSCTFTYLL GDRESREAVL IDPVLETAPR DAQLIKELGL RLLYAVNTHC  81 HADHITGSGL LRSLLPGCQS VISRLSGAQA DLHIEDGDSI RFGRFALETR ASPGHTPGCV TFVLNDHSMA FTGDALLIRG  161 CGRTDFQQGC AKTLYHSVHE KIFTLPGDCL IYPAHDYHGF TVSTVEEERT LNPRLTLSCE EFVKIMGNLN LPKPQQIDFA  241 VPANMRCGVQ TPTA |
| --- |

Peptides:

| No | Peptide | Peptide ID | Length | Mass | Charge | Intensity | Missing |
| --- | --- | --- | --- | --- | --- | --- | --- |
| 1 | GGSGAPILLR | ETHE1_17-26 | 10 | 921.54 | 2 | 66.77 | 4.6% |
| 2 | QMFEPVSCTFTYLLGDR | ETHE1_27-43 | 17 | 1987.92 | 2,3 | 9.93 | 29% |
| 3 | EAVLIDPVLETAPR | ETHE1_47-60 | 14 | 1503.83 | 2,3 | 66.24 | 1.6% |
| 4 | LLYAVNTHCHADHITGSGLLR | ETHE1_72-92 | 21 | 2272.15 | 4 | 6.33 | 59% |
| 5 | SLLPGCQSVISR | ETHE1_93-104 | 12 | 1240.66 | 2 | 68.70 | 2% |
| 6 | LSGAQADLHIEDGDSIR | ETHE1_105-121 | 17 | 1777.86 | 2,3 | 10.87 | 19.1% |
| 7 | FGRFALETR | ETHE1_122-130 | 9 | 1077.57 | 2 | 20.31 | 61.4% |
| 8 | ASPGHTPGCVTFVLNDHSMAFTGDALLIR | ETHE1_131-159 | 29 | 3008.46 | 4 | 7.93 | 59% |
| 9 | TDFQQGCAK | ETHE1_164-172 | 9 | 978.42 | 2 | 8.76 | 58.9% |
| 10 | IFTLPGDCLIYPAHDYHGFTVSTVEEER | ETHE1_182-209 | 28 | 3190.51 | 4 | 5.03 | 65% |
| 11 | LTLSCEEFVK | ETHE1_215-224 | 10 | 1149.57 | 2 | 23.21 | 8.4% |

## AGRN, Agrin ([O00468, AGRIN_HUMAN](https://www.uniprot.org/uniprot/O00468))

Coverage: 18.3% (379/2068)

| 1 MAGRSHPGPL RPLLPLLVVA ACVLPGAGGT CPERALERRE EEANVVLTGT VEEILNVDPV QHTYSCKVRV WRYLKGKDLV  81 ARESLLDGGN KVVISGFGDP LICDNQVSTG DTRIFFVNPA PPYLWPAHKN ELMLNSSLMR ITLRNLEEVE FCVEDKPGTH  161 FTPVPPTPPD ACRGMLCGFG AVCEPNAEGP GRASCVCKKS PCPSVVAPVC GSDASTYSNE CELQRAQCSQ QRRIRLLSRG  241 PCGSRDPCSN VTCSFGSTCA RSADGLTASC LCPATCRGAP EGTVCGSDGA DYPGECQLLR RACARQENVF KKFDGPCDPC  321 QGALPDPSRS CRVNPRTRRP EMLLRPESCP ARQAPVCGDD GVTYENDCVM GRSGAARGLL LQKVRSGQCQ GRDQCPEPCR  401 FNAVCLSRRG RPRCSCDRVT CDGAYRPVCA QDGRTYDSDC WRQQAECRQQ RAIPSKHQGP CDQAPSPCLG VQCAFGATCA  481 VKNGQAACEC LQACSSLYDP VCGSDGVTYG SACELEATAC TLGREIQVAR KGPCDRCGQC RFGALCEAET GRCVCPSECV  561 ALAQPVCGSD GHTYPSECML HVHACTHQIS LHVASAGPCE TCGDAVCAFG AVCSAGQCVC PRCEHPPPGP VCGSDGVTYG  641 SACELREAAC LQQTQIEEAR AGPCEQAECG SGGSGSGEDG DCEQELCRQR GGIWDEDSED GPCVCDFSCQ SVPGSPVCGS  721 DGVTYSTECE LKKARCESQR GLYVAAQGAC RGPTFAPLPP VAPLHCAQTP YGCCQDNITA ARGVGLAGCP SACQCNPHGS  801 YGGTCDPATG QCSCRPGVGG LRCDRCEPGF WNFRGIVTDG RSGCTPCSCD PQGAVRDDCE QMTGLCSCKP GVAGPKCGQC  881 PDGRALGPAG CEADASAPAT CAEMRCEFGA RCVEESGSAH CVCPMLTCPE ANATKVCGSD GVTYGNECQL KTIACRQGLQ  961 ISIQSLGPCQ EAVAPSTHPT SASVTVTTPG LLLSQALPAP PGALPLAPSS TAHSQTTPPP SSRPRTTASV PRTTVWPVLT  1041 VPPTAPSPAP SLVASAFGES GSTDGSSDEE LSGDQEASGG GSGGLEPLEG SSVATPGPPV ERASCYNSAL GCCSDGKTPS  1121 LDAEGSNCPA TKVFQGVLEL EGVEGQELFY TPEMADPKSE LFGETARSIE STLDDLFRNS DVKKDFRSVR LRDLGPGKSV  1201 RAIVDVHFDP TTAFRAPDVA RALLRQIQVS RRRSLGVRRP LQEHVRFMDF DWFPAFITGA TSGAIAAGAT ARATTASRLP  1281 SSAVTPRAPH PSHTSQPVAK TTAAPTTRRP PTTAPSRVPG RRPPAPQQPP KPCDSQPCFH GGTCQDWALG GGFTCSCPAG  1361 RGGAVCEKVL GAPVPAFEGR SFLAFPTLRA YHTLRLALEF RALEPQGLLL YNGNARGKDF LALALLDGRV QLRFDTGSGP  1441 AVLTSAVPVE PGQWHRLELS RHWRRGTLSV DGETPVLGES PSGTDGLNLD TDLFVGGVPE DQAAVALERT FVGAGLRGCI  1521 RLLDVNNQRL ELGIGPGAAT RGSGVGECGD HPCLPNPCHG GAPCQNLEAG RFHCQCPPGR VGPTCADEKS PCQPNPCHGA  1601 APCRVLPEGG AQCECPLGRE GTFCQTASGQ DGSGPFLADF NGFSHLELRG LHTFARDLGE KMALEVVFLA RGPSGLLLYN  1681 GQKTDGKGDF VSLALRDRRL EFRYDLGKGA AVIRSREPVT LGAWTRVSLE RNGRKGALRV GDGPRVLGES PKSRKVPHTV  1761 LNLKEPLYVG GAPDFSKLAR AAAVSSGFDG AIQLVSLGGR QLLTPEHVLR QVDVTSFAGH PCTRASGHPC LNGASCVPRE  1841 AAYVCLCPGG FSGPHCEKGL VEKSAGDVDT LAFDGRTFVE YLNAVTESEL ANEIPVPETL DSGALHSEKA LQSNHFELSL  1921 RTEATQGLVL WSGKATERAD YVALAIVDGH LQLSYNLGSQ PVVLRSTVPV NTNRWLRVVA HREQREGSLQ VGNEAPVTGS  2001 SPLGATQLDT DGALWLGGLP ELPVGPALPK AYGTGFVGCL RDVVVGRHPL HLLEDAVTKP ELRPCPTP |
| --- |

Peptides:

| No | Peptide | Peptide ID | Length | Mass | Charge | Intensity | Missing |
| --- | --- | --- | --- | --- | --- | --- | --- |
| 1 | ESLLDGGNK | AGRN_83-91 | 9 | 913.45 | 2 | 11.86 | 76.1% |
| 2 | VVISGFGDPLICDNQVSTGDTR | AGRN_92-113 | 22 | 2274.10 | 2,3 | 11.25 | 97.3% |
| 3 | IFFVNPAPPYLWPAHK | AGRN_114-129 | 16 | 1878.00 | 3,4 | 9.83 | 77.3% |
| 4 | FNAVCLSR | AGRN_401-408 | 8 | 890.44 | 2 | 12.70 | 97.7% |
| 5 | VTCDGAYRPVCAQDGR | AGRN_419-434 | 16 | 1691.75 | 3 | 6.72 | 99.5% |
| 6 | TYDSDCWR | AGRN_435-442 | 8 | 1026.39 | 2 | 15.64 | 99% |
| 7 | FGALCEAETGR | AGRN_542-552 | 11 | 1134.51 | 2 | 13.11 | 98.8% |
| 8 | EAACLQQTQIEEAR | AGRN_647-660 | 14 | 1570.74 | 2 | 12.00 | 97.5% |
| 9 | GLYVAAQGACR | AGRN_741-751 | 11 | 1089.54 | 2 | 16.83 | 95.3% |
| 10 | SELFGETAR | AGRN_1159-1167 | 9 | 990.48 | 2 | 17.02 | 35.5% |
| 11 | SIESTLDDLFR | AGRN_1168-1178 | 11 | 1276.63 | 2 | 20.05 | 17.4% |
| 12 | AIVDVHFDPTTAFR | AGRN_1202-1215 | 14 | 1569.79 | 2,3 | 15.41 | 19.6% |
| 13 | LPSSAVTPR | AGRN_1279-1287 | 9 | 908.51 | 2 | 7.56 | 96.2% |
| 14 | VLGAPVPAFEGR | AGRN_1369-1380 | 12 | 1193.66 | 2 | 15.76 | 27.5% |
| 15 | SFLAFPTLR | AGRN_1381-1389 | 9 | 1032.58 | 2 | 24.42 | 18.8% |
| 16 | ALEPQGLLLYNGNAR | AGRN_1402-1416 | 15 | 1609.86 | 2,3 | 4.28 | 69.1% |
| 17 | GKDFLALALLDGR | AGRN_1417-1429 | 13 | 1369.77 | 3 | 2.84 | 96.5% |
| 18 | DFLALALLDGR | AGRN_1419-1429 | 11 | 1184.66 | 2 | 7.44 | 44.9% |
| 19 | FDTGSGPAVLTSAVPVEPGQWHR | AGRN_1434-1456 | 23 | 2389.18 | 3 | 19.67 | 34.2% |
| 20 | TFVGAGLR | AGRN_1510-1517 | 8 | 801.45 | 2 | 25.90 | 33.7% |
| 21 | LLDVNNQR | AGRN_1522-1529 | 8 | 952.51 | 2 | 21.58 | 30% |
| 22 | LELGIGPGAATR | AGRN_1530-1541 | 12 | 1135.63 | 2 | 30.82 | 15.7% |
| 23 | MALEVVFLAR | AGRN_1662-1671 | 10 | 1129.63 | 2 | 4.95 | 79.5% |
| 24 | GPSGLLLYNGQK | AGRN_1672-1683 | 12 | 1227.66 | 2 | 6.19 | 92.8% |
| 25 | GDFVSLALR | AGRN_1688-1696 | 9 | 958.52 | 2 | 8.09 | 60.6% |
| 26 | SREPVTLGAWTR | AGRN_1715-1726 | 12 | 1353.72 | 3 | 17.51 | 53.4% |
| 27 | EPVTLGAWTR | AGRN_1717-1726 | 10 | 1110.58 | 2 | 10.64 | 63.8% |
| 28 | EPLYVGGAPDFSK | AGRN_1765-1777 | 13 | 1360.67 | 2 | 5.84 | 93.1% |
| 29 | AAAVSSGFDGAIQLVSLGGR | AGRN_1781-1800 | 20 | 1856.97 | 2,3 | 5.94 | 73.2% |
| 30 | QLLTPEHVLR | AGRN_1801-1810 | 10 | 1186.68 | 2,3 | 10.19 | 25.5% |
| 31 | QVDVTSFAGHPCTR | AGRN_1811-1824 | 14 | 1498.70 | 3 | 6.13 | 96.3% |
| 32 | ALQSNHFELSLR | AGRN_1910-1921 | 12 | 1395.73 | 3 | 1.92 | 97.7% |
| 33 | STVPVNTNR | AGRN_1966-1974 | 9 | 968.50 | 2 | 13.50 | 75.8% |

## STAG1, Cohesin subunit SA-1 ([Q8WVM7, STAG1_HUMAN](https://www.uniprot.org/uniprot/Q8WVM7))

Coverage: 6.6% (83/1258)

| 1 MITSELPVLQ DSTNETTAHS DAGSELEETE VKGKRKRGRP GRPPSTNKKP RKSPGEKSRI EAGIRGAGRG RANGHPQQNG  81 EGEPVTLFEV VKLGKSAMQS VVDDWIESYK QDRDIALLDL INFFIQCSGC RGTVRIEMFR NMQNAEIIRK MTEEFDEDSG  161 DYPLTMPGPQ WKKFRSNFCE FIGVLIRQCQ YSIIYDEYMM DTVISLLTGL SDSQVRAFRH TSTLAAMKLM TALVNVALNL  241 SIHQDNTQRQ YEAERNKMIG KRANERLELL LQKRKELQEN QDEIENMMNS IFKGIFVHRY RDAIAEIRAI CIEEIGVWMK  321 MYSDAFLNDS YLKYVGWTLH DRQGEVRLKC LKALQSLYTN RELFPKLELF TNRFKDRIVS MTLDKEYDVA VEAIRLVTLI  401 LHGSEEALSN EDCENVYHLV YSAHRPVAVA AGEFLHKKLF SRHDPQAEEA LAKRRGRNSP NGNLIRMLVL FFLESELHEH  481 AAYLVDSLWE SSQELLKDWE CMTELLLEEP VQGEEAMSDR QESALIELMV CTIRQAAEAH PPVGRGTGKR VLTAKERKTQ  561 IDDRNKLTEH FIITLPMLLS KYSADAEKVA NLLQIPQYFD LEIYSTGRME KHLDALLKQI KFVVEKHVES DVLEACSKTY  641 SILCSEEYTI QNRVDIARSQ LIDEFVDRFN HSVEDLLQEG EEADDDDIYN VLSTLKRLTS FHNAHDLTKW DLFGNCYRLL  721 KTGIEHGAMP EQIVVQALQC SHYSILWQLV KITDGSPSKE DLLVLRKTVK SFLAVCQQCL SNVNTPVKEQ AFMLLCDLLM  801 IFSHQLMTGG REGLQPLVFN PDTGLQSELL SFVMDHVFID QDEENQSMEG DEEDEANKIE ALHKRRNLLA AFSKLIIYDI  881 VDMHAAADIF KHYMKYYNDY GDIIKETLSK TRQIDKIQCA KTLILSLQQL FNELVQEQGP NLDRTSAHVS GIKELARRFA  961 LTFGLDQIKT REAVATLHKD GIEFAFKYQN QKGQEYPPPN LAFLEVLSEF SSKLLRQDKK TVHSYLEKFL TEQMMERRED  1041 VWLPLISYRN SLVTGGEDDR MSVNSGSSSS KTSSVRNKKG RPPLHKKRVE DESLDNTWLN RTDTMIQTPG PLPAPQLTST  1121 VLRENSRPMG DQIQEPESEH GSEPDFLHNP QMQISWLGQP KLEDLNRKDR TGMNYMKVRT GVRHAVRGLM EEDAEPIFED  1201 VMMSSRSQLE DMNEEFEDTM VIDLPPSRNR RERAELRPDF FDSAAIIEDD SGFGMPMF |
| --- |

Peptides:

| No | Peptide | Peptide ID | Length | Mass | Charge | Intensity | Missing |
| --- | --- | --- | --- | --- | --- | --- | --- |
| 1 | NMQNAEIIR | STAG1_141-149 | 9 | 1069.53 | 2 | 8.08 | 60.1% |
| 2 | SQLIDEFVDR | STAG1_659-668 | 10 | 1202.59 | 2 | 9.04 | 58% |
| 3 | REDVWLPLISYR | STAG1_1038-1049 | 12 | 1527.82 | 3 | 4.94 | 36.6% |
| 4 | EDVWLPLISYR | STAG1_1039-1049 | 11 | 1371.72 | 2 | 6.09 | 84.5% |
| 5 | NSLVTGGEDDR | STAG1_1050-1060 | 11 | 1143.52 | 2 | 6.77 | 30.8% |
| 6 | TDTMIQTPGPLPAPQLTSTVLR | STAG1_1102-1123 | 22 | 2318.23 | 3 | 3.27 | 91.5% |
| 7 | GLMEEDAEPIFEDVMMSSR | STAG1_1188-1206 | 19 | 2166.93 | 3 | 3.08 | 78.5% |

## OPA3, Optic atrophy 3 protein ([Q9H6K4, OPA3_HUMAN](https://www.uniprot.org/uniprot/Q9H6K4))

Coverage: 8.4% (15/179)

| 1 MVVGAFPMAK LLYLGIRQVS KPLANRIKEA ARRSEFFKTY ICLPPAQLYH WVEMRTKMRI MGFRGTVIKP LNEEAAAELG  81 AELLGEATIF IVGGGCLVLE YWRHQAQQRH KEEEQRAAWN ALRDEVGHLA LALEALQAQV QAAPPQGALE ELRTELQEVR  161 AQLCNPGRSA SHAVPASKK |
| --- |

Peptides:

| No | Peptide | Peptide ID | Length | Mass | Charge | Intensity | Missing |
| --- | --- | --- | --- | --- | --- | --- | --- |
| 1 | TELQEVR | OPA3_154-160 | 7 | 855.45 | 2 | 37.67 | 59.9% |
| 2 | AQLCNPGR | OPA3_161-168 | 8 | 839.41 | 2 | 19.24 | 63.8% |

## ERP29, Endoplasmic reticulum resident protein 29 ([P30040, ERP29_HUMAN](https://www.uniprot.org/uniprot/P30040))

Coverage: 46.4% (121/261)

| 1 MAAAVPRAAF LSPLLPLLLG FLLLSAPHGG SGLHTKGALP LDTVTFYKVI PKSKFVLVKF DTQYPYGEKQ DEFKRLAENS  81 ASSDDLLVAE VGISDYGDKL NMELSEKYKL DKESYPVFYL FRDGDFENPV PYTGAVKVGA IQRWLKGQGV YLGMPGCLPV  161 YDALAGEFIR ASGVEARQAL LKQGQDNLSS VKETQKKWAE QYLKIMGKIL DQGEDFPASE MTRIARLIEK NKMSDGKKEE  241 LQKSLNILTA FQKKGAEKEE L |
| --- |

Peptides:

| No | Peptide | Peptide ID | Length | Mass | Charge | Intensity | Missing |
| --- | --- | --- | --- | --- | --- | --- | --- |
| 1 | GALPLDTVTFYK | ERP29_37-48 | 12 | 1305.70 | 2 | 48.18 | 10.1% |
| 2 | FDTQYPYGEK | ERP29_60-69 | 10 | 1228.54 | 2 | 30.32 | 67.1% |
| 3 | LNMELSEK | ERP29_100-107 | 8 | 944.46 | 2 | 29.30 | 58.1% |
| 4 | ESYPVFYLFR | ERP29_113-122 | 10 | 1301.64 | 2 | 42.84 | 71.3% |
| 5 | DGDFENPVPYTGAVK | ERP29_123-137 | 15 | 1589.74 | 2 | 31.25 | 10.3% |
| 6 | GQGVYLGMPGCLPVYDALAGEFIR | ERP29_147-170 | 24 | 2507.23 | 3 | 21.35 | 51.2% |
| 7 | QGQDNLSSVK | ERP29_183-192 | 10 | 1056.52 | 2 | 13.87 | 49.2% |
| 8 | WAEQYLK | ERP29_198-204 | 7 | 918.46 | 2 | 47.95 | 77.2% |
| 9 | ILDQGEDFPASEMTR | ERP29_209-223 | 15 | 1689.77 | 2 | 27.18 | 6% |
| 10 | SLNILTAFQK | ERP29_244-253 | 10 | 1115.63 | 2 | 43.44 | 2.2% |

## GAPVD1, GTPase-activating protein and VPS9 domain-containing protein 1 ([Q14C86, GAPD1_HUMAN](https://www.uniprot.org/uniprot/Q14C86))

Coverage: 9.9% (146/1478)

| 1 MVKLDIHTLA HHLKQERLYV NSEKQLIQRL NADVLKTAEK LYRTAWIAKQ QRINLDRLII TSAEASPAEC CQHAKILEDT  81 QFVDGYKQLG FQETAYGEFL SRLRENPRLI ASSLVAGEKL NQENTQSVIY TVFTSLYGNC IMQEDESYLL QVLRYLIEFE  161 LKESDNPRRL LRRGTCAFSI LFKLFSEGLF SAKLFLTATL HEPIMQLLVE DEDHLETDPN KLIERFSPSQ QEKLFGEKGS  241 DRFRQKVQEM VESNEAKLVA LVNKFIGYLK QNTYCFPHSL RWIVSQMYKT LSCVDRLEVG EVRAMCTDLL LACFICPAVV  321 NPEQYGIISD APINEVARFN LMQVGRLLQQ LAMTGSEEGD PRTKSSLGKF DKSCVAAFLD VVIGGRAVET PPLSSVNLLE  401 GLSRTVVYIT YSQLITLVNF MKSVMSGDQL REDRMALDNL LANLPPAKPG KSSSLEMTPY NTPQLSPATT PANKKNRLPI  481 ATRSRSRTNM LMDLHMDHEG SSQETIQEVQ PEEVLVISLG TGPQLTPGMM SENEVLNMQL SDGGQGDVPV DENKLHGKPD  561 KTLRFSLCSD NLEGISEGPS NRSNSVSSLD LEGESVSELG AGPSGSNGVE ALQLLEHEQA TTQDNLDDKL RKFEIRDMMG  641 LTDDRDISET VSETWSTDVL GSDFDPNIDE DRLQEIAGAA AENMLGSLLC LPGSGSVLLD PCTGSTISET TSEAWSVEVL  721 PSDSEAPDLK QEERLQELES CSGLGSTSDD TDVREVSSRP STPGLSVVSG ISATSEDIPN KIEDLRSECS SDFGGKDSVT  801 SPDMDEITHG AHQLTSPPSQ SESLLAMFDP LSSHEGASAV VRPKVHYARP SHPPPDPPIL EGAVGGNEAR LPNFGSHVLT  881 PAEMEAFKQR HSYPERLVRS RSSDIVSSVR RPMSDPSWNR RPGNEERELP PAAAIGATSL VAAPHSSSSS PSKDSSRGET  961 EERKDSDDEK SDRNRPWWRK RFVSAMPKAP IPFRKKEKQE KDKDDLGPDR FSTLTDDPSP RLSAQAQVAE DILDKYRNAI  1041 KRTSPSDGAM ANYESTGDNH DRDLSSKLLY HSDKEVMGDG ESAHDSPRDE ALQNISADDL PDSASQAAHP QDSAFSYRDA  1121 KKKLRLALCS ADSVAFPVLT HSTRNGLPDH TDPEDNEIVC FLKVQIAEAI NLQDKNLMAQ LQETMRCVCR FDNRTCRKLL  1201 ASIAEDYRKR APYIAYLTRC RQGLQTTQAH LERLLQRVLR DKEVANRYFT TVCVRLLLES KEKKIREFIQ DFQKLTAADD  1281 KTAQVEDFLQ FLYGAMAQDV IWQNASEEQL QDAQLAIERS VMNRIFKLAF YPNQDGDILR DQVLHEHIQR LSKVVTANHR  1361 ALQIPEVYLR EAPWPSAQSE IRTISAYKTP RDKVQCILRM CSTIMNLLSL ANEDSVPGAD DFVPVLVFVL IKANPPCLLS  1441 TVQYISSFYA SCLSGEESYW WMQFTAAVEF IKTIDDRK |
| --- |

Peptides:

| No | Peptide | Peptide ID | Length | Mass | Charge | Intensity | Missing |
| --- | --- | --- | --- | --- | --- | --- | --- |
| 1 | LIASSLVAGEK | GAPVD1_109-119 | 11 | 1068.62 | 2 | 17.81 | 43% |
| 2 | FNLMQVGR | GAPVD1_339-346 | 8 | 945.49 | 2 | 19.22 | 6.3% |
| 3 | AVETPPLSSVNLLEGLSR | GAPVD1_387-404 | 18 | 1863.01 | 2 | 16.12 | 6.7% |
| 4 | DMMGLTDDR | GAPVD1_637-645 | 9 | 1034.42 | 2 | 11.17 | 52% |
| 5 | LQELESCSGLGSTSDDTDVR | GAPVD1_735-754 | 20 | 2092.92 | 2 | 3.82 | 81.7% |
| 6 | SECSSDFGGK | GAPVD1_787-796 | 10 | 997.38 | 2 | 5.72 | 71.8% |
| 7 | SSDIVSSVR | GAPVD1_902-910 | 9 | 930.48 | 2 | 18.53 | 14.9% |
| 8 | FSTLTDDPSPR | GAPVD1_1011-1021 | 11 | 1216.57 | 2 | 10.86 | 29.2% |
| 9 | LALCSADSVAFPVLTHSTR | GAPVD1_1126-1144 | 19 | 1969.01 | 3 | 15.55 | 64.4% |
| 10 | APYIAYLTR | GAPVD1_1211-1219 | 9 | 1048.57 | 2 | 8.45 | 68.4% |
| 11 | ALQIPEVYLR | GAPVD1_1361-1370 | 10 | 1182.68 | 2 | 33.20 | 1.6% |
| 12 | EAPWPSAQSEIR | GAPVD1_1371-1382 | 12 | 1351.65 | 2 | 19.70 | 50.7% |

## ALDOA, Fructose-bisphosphate aldolase A ([P04075, ALDOA_HUMAN](https://www.uniprot.org/uniprot/P04075))

Coverage: 69.5% (253/364)

| 1 MPYQYPALTP EQKKELSDIA HRIVAPGKGI LAADESTGSI AKRLQSIGTE NTEENRRFYR QLLLTADDRV NPCIGGVILF  81 HETLYQKADD GRPFPQVIKS KGGVVGIKVD KGVVPLAGTN GETTTQGLDG LSERCAQYKK DGADFAKWRC VLKIGEHTPS  161 ALAIMENANV LARYASICQQ NGIVPIVEPE ILPDGDHDLK RCQYVTEKVL AAVYKALSDH HIYLEGTLLK PNMVTPGHAC  241 TQKFSHEEIA MATVTALRRT VPPAVTGITF LSGGQSEEEA SINLNAINKC PLLKPWALTF SYGRALQASA LKAWGGKKEN  321 LKAAQEEYVK RALANSLACQ GKYTPSGQAG AAASESLFVS NHAY |
| --- |

Peptides:

| No | Peptide | Peptide ID | Length | Mass | Charge | Intensity | Missing |
| --- | --- | --- | --- | --- | --- | --- | --- |
| 1 | KELSDIAHR | ALDOA_14-22 | 9 | 1049.56 | 2 | 27.56 | 3.3% |
| 2 | ELSDIAHR | ALDOA_15-22 | 8 | 921.47 | 2 | 145.36 | 0% |
| 3 | IVAPGKGILAADESTGSIAK | ALDOA_23-42 | 20 | 1879.04 | 3 | 3.76 | 66.1% |
| 4 | GILAADESTGSIAK | ALDOA_29-42 | 14 | 1313.68 | 2,3 | 445.36 | 0% |
| 5 | GILAADESTGSIAKR | ALDOA_29-43 | 15 | 1469.78 | 2,3 | 9.37 | 11.1% |
| 6 | RLQSIGTENTEENR | ALDOA_43-56 | 14 | 1627.79 | 3 | 11.99 | 12.4% |
| 7 | LQSIGTENTEENR | ALDOA_44-56 | 13 | 1471.69 | 2 | 102.49 | 0% |
| 8 | LQSIGTENTEENRR | ALDOA_44-57 | 14 | 1627.79 | 2,3 | 263.25 | 0% |
| 9 | QLLLTADDR | ALDOA_61-69 | 9 | 1025.55 | 2 | 691.38 | 0% |
| 10 | QLLLTADDRVNPCIGGVILFHETLYQK | ALDOA_61-87 | 27 | 3037.61 | 4 | 27.29 | 86.4% |
| 11 | VNPCIGGVILFHETLYQK | ALDOA_70-87 | 18 | 2012.06 | 2,3 | 30.26 | 4.4% |
| 12 | ADDGRPFPQVIK | ALDOA_88-99 | 12 | 1323.69 | 2,3 | 138.71 | 0% |
| 13 | PFPQVIK | ALDOA_93-99 | 7 | 809.48 | 2 | 11.71 | 53.7% |
| 14 | SKGGVVGIK | ALDOA_100-108 | 9 | 825.51 | 2 | 9.74 | 91.9% |
| 15 | VDKGVVPLAGTNGETTTQGLDGLSER | ALDOA_109-134 | 26 | 2595.31 | 3 | 28.01 | 22.2% |
| 16 | GVVPLAGTNGETTTQGLDGLSER | ALDOA_112-134 | 23 | 2253.12 | 2,3 | 60.85 | 0.5% |
| 17 | CVLKIGEHTPSALAIMENANVLAR | ALDOA_150-173 | 24 | 2531.34 | 4 | 3.02 | 80.2% |
| 18 | IGEHTPSALAIMENANVLAR | ALDOA_154-173 | 20 | 2088.08 | 2,3,4 | 374.90 | 0% |
| 19 | FSHEEIAMATVTALR | ALDOA_244-258 | 15 | 1656.83 | 2,3,4 | 274.37 | 0% |
| 20 | FSHEEIAMATVTALRR | ALDOA_244-259 | 16 | 1812.93 | 3,4 | 60.54 | 0.5% |
| 21 | TVPPAVTGITFLSGGQSEEEASINLNAINK | ALDOA_260-289 | 30 | 3038.56 | 3,4 | 30.22 | 1.6% |
| 22 | CPLLKPWALTFSYGR | ALDOA_290-304 | 15 | 1732.91 | 2,3 | 93.25 | 1.2% |
| 23 | ALQASALK | ALDOA_305-312 | 8 | 782.47 | 2 | 706.29 | 38.9% |
| 24 | AAQEEYVK | ALDOA_323-330 | 8 | 918.44 | 2 | 80.68 | 0.7% |
| 25 | AAQEEYVKR | ALDOA_323-331 | 9 | 1074.55 | 2 | 12.28 | 8.2% |
| 26 | RALANSLACQGK | ALDOA_331-342 | 12 | 1212.64 | 2,3 | 64.39 | 1.8% |
| 27 | ALANSLACQGK | ALDOA_332-342 | 11 | 1056.54 | 2 | 343.04 | 0% |
| 28 | YTPSGQAGAAASESLFVSNHAY | ALDOA_343-364 | 22 | 2209.01 | 2,3 | 41.45 | 0% |

## DECR1, 2,4-dienoyl-CoA reductase [(3E)-enoyl-CoA-producing], mitochondrial ([Q16698, DECR_HUMAN](https://www.uniprot.org/uniprot/Q16698))

Coverage: 44.8% (150/335)

| 1 MKLPARVFFT LGSRLPCGLA PRRFFSYGTK ILYQNTEALQ SKFFSPLQKA MLPPNSFQGK VAFITGGGTG LGKGMTTLLS  81 SLGAQCVIAS RKMDVLKATA EQISSQTGNK VHAIQCDVRD PDMVQNTVSE LIKVAGHPNI VINNAAGNFI SPTERLSPNA  161 WKTITDIVLN GTAFVTLEIG KQLIKAQKGA AFLSITTIYA ETGSGFVVPS ASAKAGVEAM SKSLAAEWGK YGMRFNVIQP  241 GPIKTKGAFS RLDPTGTFEK EMIGRIPCGR LGTVEELANL AAFLCSDYAS WINGAVIKFD GGEEVLISGE FNDLRKVTKE  321 QWDTIEELIR KTKGS |
| --- |

Peptides:

| No | Peptide | Peptide ID | Length | Mass | Charge | Intensity | Missing |
| --- | --- | --- | --- | --- | --- | --- | --- |
| 1 | FFSPLQK | DECR1_43-49 | 7 | 847.46 | 2 | 27.04 | 7.4% |
| 2 | VAFITGGGTGLGK | DECR1_61-73 | 13 | 1158.64 | 2 | 40.07 | 2% |
| 3 | GMTTLLSSLGAQCVIASR | DECR1_74-91 | 18 | 1788.92 | 2,3 | 14.78 | 3.7% |
| 4 | ATAEQISSQTGNK | DECR1_98-110 | 13 | 1315.64 | 2 | 11.47 | 2.5% |
| 5 | DPDMVQNTVSELIK | DECR1_120-133 | 14 | 1569.77 | 2 | 9.19 | 25.8% |
| 6 | VAGHPNIVINNAAGNFISPTER | DECR1_134-155 | 22 | 2272.17 | 3 | 17.98 | 2.9% |
| 7 | LSPNAWK | DECR1_156-162 | 7 | 796.42 | 2 | 25.04 | 12.7% |
| 8 | SLAAEWGK | DECR1_223-230 | 8 | 842.43 | 2 | 23.51 | 44.6% |
| 9 | FNVIQPGPIK | DECR1_235-244 | 10 | 1093.63 | 2 | 26.02 | 0.7% |
| 10 | LDPTGTFEK | DECR1_252-260 | 9 | 988.49 | 2 | 33.76 | 0.4% |
| 11 | FDGGEEVLISGEFNDLRK | DECR1_299-316 | 18 | 2005.97 | 3 | 9.66 | 92.3% |
| 12 | EQWDTIEELIR | DECR1_320-330 | 11 | 1412.69 | 2 | 16.29 | 7.4% |

## MVP, Major vault protein ([Q14764, MVP_HUMAN](https://www.uniprot.org/uniprot/Q14764))

Coverage: 61.1% (546/893)

| 1 MATEEFIIRI PPYHYIHVLD QNSNVSRVEV GPKTYIRQDN ERVLFAPMRM VTVPPRHYCT VANPVSRDAQ GLVLFDVTGQ  81 VRLRHADLEI RLAQDPFPLY PGEVLEKDIT PLQVVLPNTA LHLKALLDFE DKDGDKVVAG DEWLFEGPGT YIPRKEVEVV  161 EIIQATIIRQ NQALRLRARK ECWDRDGKER VTGEEWLVTT VGAYLPAVFE EVLDLVDAVI LTEKTALHLR ARRNFRDFRG  241 VSRRTGEEWL VTVQDTEAHV PDVHEEVLGV VPITTLGPHN YCVILDPVGP DGKNQLGQKR VVKGEKSFFL QPGEQLEQGI  321 QDVYVLSEQQ GLLLRALQPL EEGEDEEKVS HQAGDHWLIR GPLEYVPSAK VEVVEERQAI PLDENEGIYV QDVKTGKVRA  401 VIGSTYMLTQ DEVLWEKELP PGVEELLNKG QDPLADRGEK DTAKSLQPLA PRNKTRVVSY RVPHNAAVQV YDYREKRARV  481 VFGPELVSLG PEEQFTVLSL SAGRPKRPHA RRALCLLLGP DFFTDVITIE TADHARLQLQ LAYNWHFEVN DRKDPQETAK  561 LFSVPDFVGD ACKAIASRVR GAVASVTFDD FHKNSARIIR TAVFGFETSE AKGPDGMALP RPRDQAVFPQ NGLVVSSVDV  641 QSVEPVDQRT RDALQRSVQL AIEITTNSQE AAAKHEAQRL EQEARGRLER QKILDQSEAE KARKELLELE ALSMAVESTG  721 TAKAEAESRA EAARIEGEGS VLQAKLKAQA LAIETEAELQ RVQKVRELEL VYARAQLELE VSKAQQLAEV EVKKFKQMTE  801 AIGPSTIRDL AVAGPEMQVK LLQSLGLKST LITDGSTPIN LFNTAFGLLG MGPEGQPLGR RVASGPSPGE GISPQSAQAP  881 QAPGDNHVVP VLR |
| --- |

Peptides:

| No | Peptide | Peptide ID | Length | Mass | Charge | Intensity | Missing |
| --- | --- | --- | --- | --- | --- | --- | --- |
| 1 | IPPYHYIHVLDQNSNVSR | MVP_10-27 | 18 | 2133.08 | 3,4 | 22.09 | 16.7% |
| 2 | VLFAPMR | MVP_43-49 | 7 | 814.45 | 2 | 320.02 | 15.9% |
| 3 | MVTVPPR | MVP_50-56 | 7 | 780.43 | 2 | 37.53 | 42% |
| 4 | HYCTVANPVSR | MVP_57-67 | 11 | 1227.58 | 2,3 | 38.67 | 16.2% |
| 5 | DAQGLVLFDVTGQVR | MVP_68-82 | 15 | 1598.84 | 2,3 | 44.00 | 15.9% |
| 6 | HADLEIR | MVP_85-91 | 7 | 834.43 | 2 | 119.67 | 16.1% |
| 7 | LAQDPFPLYPGEVLEK | MVP_92-107 | 16 | 1796.93 | 2,3 | 54.81 | 16.1% |
| 8 | DITPLQVVLPNTALHLK | MVP_108-124 | 17 | 1853.08 | 2,3 | 17.75 | 17% |
| 9 | ALLDFEDK | MVP_125-132 | 8 | 931.47 | 2 | 27.41 | 17.8% |
| 10 | VVAGDEWLFEGPGTYIPR | MVP_137-154 | 18 | 1986.98 | 2,3 | 23.26 | 18.9% |
| 11 | KEVEVVEIIQATIIR | MVP_155-169 | 15 | 1721.01 | 2,3 | 24.55 | 16.2% |
| 12 | EVEVVEIIQATIIR | MVP_156-169 | 14 | 1592.91 | 2,3 | 15.67 | 16.4% |
| 13 | SFFLQPGEQLEQGIQDVYVLSEQQGLLLR | MVP_307-335 | 29 | 3315.71 | 3 | 5.92 | 81.9% |
| 14 | ALQPLEEGEDEEK | MVP_336-348 | 13 | 1467.67 | 2 | 60.95 | 16.1% |
| 15 | VSHQAGDHWLIR | MVP_349-360 | 12 | 1399.71 | 2,3 | 9.62 | 59.7% |
| 16 | GPLEYVPSAK | MVP_361-370 | 10 | 1041.55 | 2 | 86.27 | 15.8% |
| 17 | VEVVEER | MVP_371-377 | 7 | 840.43 | 2 | 207.17 | 16.6% |
| 18 | QAIPLDENEGIYVQDVK | MVP_378-394 | 17 | 1911.96 | 2,3 | 19.02 | 15.3% |
| 19 | AVIGSTYMLTQDEVLWEK | MVP_400-417 | 18 | 2064.02 | 2 | 8.66 | 59.4% |
| 20 | ELPPGVEELLNK | MVP_418-429 | 12 | 1318.71 | 2 | 38.79 | 16.3% |
| 21 | GQDPLADR | MVP_430-437 | 8 | 852.41 | 2 | 27.43 | 43.6% |
| 22 | SLQPLAPR | MVP_445-452 | 8 | 862.50 | 2 | 83.86 | 15.9% |
| 23 | VPHNAAVQVYDYR | MVP_462-474 | 13 | 1512.75 | 2,3 | 41.02 | 16.4% |
| 24 | VVFGPELVSLGPEEQFTVLSLSAGR | MVP_480-504 | 25 | 2612.38 | 3 | 22.58 | 18.9% |
| 25 | VVFGPELVSLGPEEQFTVLSLSAGRPK | MVP_480-506 | 27 | 2837.53 | 3 | 11.27 | 36.8% |
| 26 | RALCLLLGPDFFTDVITIETADHAR | MVP_512-536 | 25 | 2768.43 | 4 | 9.00 | 76.1% |
| 27 | ALCLLLGPDFFTDVITIETADHAR | MVP_513-536 | 24 | 2612.33 | 3,4 | 9.95 | 61.5% |
| 28 | LFSVPDFVGDACK | MVP_561-573 | 13 | 1378.66 | 2,3 | 35.56 | 16.3% |
| 29 | VRGAVASVTFDDFHK | MVP_579-593 | 15 | 1629.83 | 4 | 2.08 | 90.7% |
| 30 | GAVASVTFDDFHK | MVP_581-593 | 13 | 1374.66 | 2,3 | 40.78 | 15.4% |
| 31 | TAVFGFETSEAK | MVP_601-612 | 12 | 1267.61 | 2 | 110.14 | 15.3% |
| 32 | GPDGMALPR | MVP_613-621 | 9 | 894.44 | 2 | 92.13 | 17.1% |
| 33 | DQAVFPQNGLVVSSVDVQSVEPVDQR | MVP_624-649 | 26 | 2793.39 | 3 | 42.52 | 15.9% |
| 34 | SVQLAIEITTNSQEAAAK | MVP_657-674 | 18 | 1854.97 | 2 | 4.78 | 77.3% |
| 35 | ILDQSEAEK | MVP_693-701 | 9 | 1013.50 | 2 | 22.75 | 18.5% |
| 36 | KELLELEALSMAVESTGTAK | MVP_704-723 | 20 | 2101.10 | 3 | 7.04 | 62.5% |
| 37 | ELLELEALSMAVESTGTAK | MVP_705-723 | 19 | 1973.00 | 2,3 | 12.79 | 19.1% |
| 38 | IEGEGSVLQAK | MVP_735-745 | 11 | 1111.59 | 2 | 27.17 | 15.8% |
| 39 | AQALAIETEAELQR | MVP_748-761 | 14 | 1523.79 | 2,3 | 9.84 | 26.6% |
| 40 | VRELELVYAR | MVP_765-774 | 10 | 1228.69 | 3 | 6.09 | 78.6% |
| 41 | ELELVYAR | MVP_767-774 | 8 | 973.52 | 2 | 106.93 | 16.1% |
| 42 | AQLELEVSK | MVP_775-783 | 9 | 997.54 | 2 | 33.12 | 18.9% |
| 43 | AQQLAEVEVK | MVP_784-793 | 10 | 1095.59 | 2 | 7.96 | 65.1% |
| 44 | QMTEAIGPSTIR | MVP_797-808 | 12 | 1284.65 | 2 | 13.16 | 21.3% |
| 45 | DLAVAGPEMQVK | MVP_809-820 | 12 | 1238.63 | 2 | 36.33 | 16.6% |
| 46 | LLQSLGLK | MVP_821-828 | 8 | 852.54 | 2 | 20.03 | 20.6% |

## CDS2, Phosphatidate cytidylyltransferase 2 ([O95674, CDS2_HUMAN](https://www.uniprot.org/uniprot/O95674))

Coverage: 5.4% (24/445)

| 1 MTELRQRVAH EPVAPPEDKE SESEAKVDGE TASDSESRAE SAPLPVSADD TPEVLNRALS NLSSRWKNWW VRGILTLAMI  81 AFFFIIIYLG PMVLMIIVMC VQIKCFHEII TIGYNVYHSY DLPWFRTLSW YFLLCVNYFF YGETVTDYFF TLVQREEPLR  161 ILSKYHRFIS FTLYLIGFCM FVLSLVKKHY RLQFYMFGWT HVTLLIVVTQ SHLVIHNLFE GMIWFIVPIS CVICNDIMAY  241 MFGFFFGRTP LIKLSPKKTW EGFIGGFFAT VVFGLLLSYV MSGYRCFVCP VEYNNDTNSF TVDCEPSDLF RLQEYNIPGV  321 IQSVIGWKTV RMYPFQIHSI ALSTFASLIG PFGGFFASGF KRAFKIKDFA NTIPGHGGIM DRFDCQYLMA TFVNVYIASF  401 IRGPNPSKLI QQFLTLRPDQ QLHIFNTLRS HLIDKGMLTS TTEDE |
| --- |

Peptides:

| No | Peptide | Peptide ID | Length | Mass | Charge | Intensity | Missing |
| --- | --- | --- | --- | --- | --- | --- | --- |
| 1 | VAHEPVAPPEDK | CDS2_8-19 | 12 | 1269.64 | 2,3 | 12.27 | 22.1% |
| 2 | VDGETASDSESR | CDS2_27-38 | 12 | 1233.51 | 2 | 7.72 | 4.6% |

## WDR81, WD repeat-containing protein 81 ([Q562E7, WDR81_HUMAN](https://www.uniprot.org/uniprot/Q562E7))

Coverage: 9.8% (190/1941)

| 1 MAQGSGGREG ALRTPAGGWH SPPSPDMQEL LRSVERDLSI DPRQLAPAPG GTHVVALVPA RWLASLRDRR LPLGPCPRAE  81 GLGEAEVRTL LQRSVQRLPA GWTRVEVHGL RKRRLSYPLG GGLPFEDGSC GPETLTRFMQ EVAAQNYRNL WRHAYHTYGQ  161 PYSHSPAPSA VPALDSVRQA LQRVYGCSFL PVGETTQCPS YAREGPCPPR GSPACPSLLR AEALLESPEM LYVVHPYVQF  241 SLHDVVTFSP AKLTNSQAKV LFILFRVLRA MDACHRQGLA CGALSLYHIA VDEKLCSELR LDLSAYERPE EDENEEAPVA  321 RDEAGIVSQE EQGGQPGQPT GQEELRSLVL DWVHGRISNF HYLMQLNRLA GRRQGDPNYH PVLPWVVDFT TPHGRFRDLR  401 KSKFRLNKGD KQLDFTYEMT RQAFVAGGAG GGEPPHVPHH ISDVLSDITY YVYKARRTPR SVLCGHVRAQ WEPHEYPASM  481 ERMQNWTPDE CIPEFYTDPS IFRSIHPDMP DLDVPAWCSS SQEFVAAHRA LLESREVSRD LHHWIDLTFG YKLQGKEAVK  561 EKNVCLHLVD AHTHLASYGV VQLFDQPHPQ RLAGAPALAP EPPLIPKLLV QTIQETTGRE DFTENPGQLP NGVGRPVLEA  641 TPCEASWTRD RPVAGEDDLE QATEALDSIS LAGKAGDQLG SSSQASPGLL SFSVASASRP GRRNKAAGAD PGEGEEGRIL  721 LPEGFNPMQA LEELEKTGNF LAKGLGGLLE VPEQPRVQPA VPLQCLLHRD MQALGVLLAE MVFATRVRTL QPDAPLWVRF  801 QAVRGLCTRH PKEVPVSLQP VLDTLLQMSG PEVPMGAERG KLDQLFEYRP VSQGLPPPCP SQLLSPFSSV VPFPPYFPAL  881 HRFILLYQAR RVEDEAQGRE LVFALWQQLG AVLKDITPEG LEILLPFVLS LMSEEHTAVY TAWYLFEPVA KALGPKNANK  961 YLLKPLIGAY ESPCQLHGRF YLYTDCFVAQ LMVRLGLQAF LTHLLPHVLQ VLAGAEASQE ESKDLAGAAE EEESGLPGAG  1041 PGSCAFGEEI PMDGEPPASS GLGLPDYTSG VSFHDQADLP ETEDFQAGLY VTESPQPQEA EAVSLGRLSD KSSTSETSLG  1121 EERAPDEGGA PVDKSSLRSG DSSQDLKQSE GSEEEEEEED SCVVLEEEEG EQEEVTGASE LTLSDTVLSM ETVVAGGSGG  1201 DGEEEEEALP EQSEGKEQKI LLDTACKMVR WLSAKLGPTV ASRHVARNLL RLLTSCYVGP TRQQFTVSSG ESPPLSAGNI  1281 YQKRPVLGDI VSGPVLSCLL HIARLYGEPV LTYQYLPYIS YLVAPGSASG PSRLNSRKEA GLLAAVTLTQ KIIVYLSDTT  1361 LMDILPRISH EVLLPVLSFL TSLVTGFPSG AQARTILCVK TISLIALICL RIGQEMVQQH LSEPVATFFQ VFSQLHELRQ  1441 QDLKLDPAGR GEGQLPQVVF SDGQQRPVDP ALLDELQKVF TLEMAYTIYV PFSCLLGDII RKIIPNHELV GELAALYLES  1521 ISPSSRNPAS VEPTMPGTGP EWDPHGGGCP QDDGHSGTFG SVLVGNRIQI PNDSRPENPG PLGPISGVGG GGLGSGSDDN  1601 ALKQELPRSV HGLSGNWLAY WQYEIGVSQQ DAHFHFHQIR LQSFPGHSGA VKCVAPLSSE DFFLSGSKDR TVRLWPLYNY  1681 GDGTSETAPR LVYTQHRKSV FFVGQLEAPQ HVVSCDGAVH VWDPFTGKTL RTVEPLDSRV PLTAVAVMPA PHTSITMASS  1761 DSTLRFVDCR KPGLQHEFRL GGGLNPGLVR ALAISPSGRS VVAGFSSGFM VLLDTRTGLV LRGWPAHEGD ILQIKAVEGS  1841 VLVSSSSDHS LTVWKELEQK PTHHYKSASD PIHTFDLYGS EVVTGTVSNK IGVCSLLEPP SQATTKLSSE NFRGTLTSLA  1921 LLPTKRHLLL GSDNGVIRLL A |
| --- |

Peptides:

| No | Peptide | Peptide ID | Length | Mass | Charge | Intensity | Missing |
| --- | --- | --- | --- | --- | --- | --- | --- |
| 1 | QLAPAPGGTHVVALVPAR | WDR81_44-61 | 18 | 1734.99 | 3 | 7.94 | 34.7% |
| 2 | AEGLGEAEVR | WDR81_79-88 | 10 | 1011.50 | 2 | 19.49 | 33.8% |
| 3 | LPAGWTR | WDR81_98-104 | 7 | 781.42 | 2 | 10.31 | 41% |
| 4 | FMQEVAAQNYR | WDR81_138-148 | 11 | 1337.62 | 2 | 4.57 | 75.6% |
| 5 | GSPACPSLLR | WDR81_211-220 | 10 | 981.51 | 2 | 9.53 | 44.4% |
| 6 | DEAGIVSQEEQGGQPGQPTGQEELR | WDR81_322-346 | 25 | 2620.20 | 3 | 2.70 | 96.2% |
| 7 | LAGAPALAPEPPLIPK | WDR81_592-607 | 16 | 1535.91 | 2 | 1.96 | 93.3% |
| 8 | AAGADPGEGEEGR | WDR81_706-718 | 13 | 1196.51 | 2 | 4.24 | 73.9% |
| 9 | GLGGLLEVPEQPR | WDR81_744-756 | 13 | 1345.74 | 2 | 7.10 | 47.9% |
| 10 | VQPAVPLQCLLHR | WDR81_757-769 | 13 | 1454.82 | 3 | 4.34 | 77.5% |
| 11 | TLQPDAPLWVR | WDR81_789-799 | 11 | 1276.69 | 2 | 9.91 | 43% |
| 12 | LLTSCYVGPTR | WDR81_1252-1262 | 11 | 1190.61 | 2 | 4.79 | 63.6% |
| 13 | LGGGLNPGLVR | WDR81_1780-1790 | 11 | 1033.60 | 2 | 7.05 | 25.1% |
| 14 | ALAISPSGR | WDR81_1791-1799 | 9 | 852.48 | 2 | 16.37 | 45.4% |
| 15 | HLLLGSDNGVIR | WDR81_1927-1938 | 12 | 1274.71 | 3 | 1.82 | 85.6% |

## KRT17, Keratin, type I cytoskeletal 17 ([Q04695, K1C17_HUMAN](https://www.uniprot.org/uniprot/Q04695))

Coverage: 49.3% (213/432)

| 1 MTTSIRQFTS SSSIKGSSGL GGGSSRTSCR LSGGLGAGSC RLGSAGGLGS TLGGSSYSSC YSFGSGGGYG SSFGGVDGLL  81 AGGEKATMQN LNDRLASYLD KVRALEEANT ELEVKIRDWY QRQAPGPARD YSQYYRTIEE LQNKILTATV DNANILLQID  161 NARLAADDFR TKFETEQALR LSVEADINGL RRVLDELTLA RADLEMQIEN LKEELAYLKK NHEEEMNALR GQVGGEINVE  241 MDAAPGVDLS RILNEMRDQY EKMAEKNRKD AEDWFFSKTE ELNREVATNS ELVQSGKSEI SELRRTMQAL EIELQSQLSM  321 KASLEGNLAE TENRYCVQLS QIQGLIGSVE EQLAQLRCEM EQQNQEYKIL LDVKTRLEQE IATYRRLLEG EDAHLTQYKK  401 EPVTTRQVRT IVEEVQDGKV ISSREQVHQT TR |
| --- |

Peptides:

| No | Peptide | Peptide ID | Length | Mass | Charge | Intensity | Missing |
| --- | --- | --- | --- | --- | --- | --- | --- |
| 1 | QFTSSSSIK | KRT17_7-15 | 9 | 965.48 | 2 | 164.52 | 20.8% |
| 2 | QFTSSSSIKGSSGLGGGSSR | KRT17_7-26 | 20 | 1867.90 | 3 | 9.50 | 89.8% |
| 3 | GSSGLGGGSSR | KRT17_16-26 | 11 | 902.42 | 2 | 10.09 | 98.8% |
| 4 | LSGGLGAGSCR | KRT17_31-41 | 11 | 958.47 | 2 | 442.22 | 20% |
| 5 | ALEEANTELEVK | KRT17_104-115 | 12 | 1326.67 | 2 | 43.41 | 34.3% |
| 6 | DYSQYYR | KRT17_130-136 | 7 | 975.41 | 2 | 103.70 | 44.5% |
| 7 | TIEELQNK | KRT17_137-144 | 8 | 955.50 | 2 | 82.38 | 44.4% |
| 8 | TIEELQNKILTATVDNANILLQIDNAR | KRT17_137-163 | 27 | 3004.62 | 4 | 12.29 | 84.5% |
| 9 | ILTATVDNANILLQIDNAR | KRT17_145-163 | 19 | 2049.12 | 2,3 | 24.85 | 42.7% |
| 10 | LSVEADINGLR | KRT17_181-191 | 11 | 1167.62 | 2 | 74.37 | 37.3% |
| 11 | LSVEADINGLRR | KRT17_181-192 | 12 | 1323.73 | 2,3 | 45.52 | 40.7% |
| 12 | ADLEMQIENLK | KRT17_202-212 | 11 | 1284.64 | 2 | 15.13 | 94.4% |
| 13 | GQVGGEINVEMDAAPGVDLSR | KRT17_231-251 | 21 | 2095.00 | 2,3 | 44.45 | 52.7% |
| 14 | KDAEDWFFSK | KRT17_269-278 | 10 | 1253.57 | 2,3 | 10.19 | 81.3% |
| 15 | DAEDWFFSK | KRT17_270-278 | 9 | 1125.48 | 2 | 43.66 | 34.2% |
| 16 | TMQALEIELQSQLSMK | KRT17_306-321 | 16 | 1830.92 | 2,3 | 19.16 | 57.3% |
| 17 | ASLEGNLAETENR | KRT17_322-334 | 13 | 1384.66 | 2,3 | 31.16 | 40.1% |
| 18 | YCVQLSQIQGLIGSVEEQLAQLR | KRT17_335-357 | 23 | 2556.34 | 3 | 7.73 | 96.3% |
| 19 | RLLEGEDAHLTQYK | KRT17_386-399 | 14 | 1653.85 | 3 | 11.66 | 74.2% |
| 20 | LLEGEDAHLTQYK | KRT17_387-399 | 13 | 1497.75 | 2,3 | 23.77 | 40.3% |
| 21 | LLEGEDAHLTQYKK | KRT17_387-400 | 14 | 1625.84 | 3 | 3.82 | 97.1% |
| 22 | TIVEEVQDGK | KRT17_410-419 | 10 | 1098.56 | 2 | 381.20 | 21.7% |
| 23 | TIVEEVQDGKVISSR | KRT17_410-424 | 15 | 1640.87 | 2,3 | 13.23 | 82.5% |

## MMP2, 72 kDa type IV collagenase ([P08253, MMP2_HUMAN](https://www.uniprot.org/uniprot/P08253))

Coverage: 6.8% (45/660)

| 1 MEALMARGAL TGPLRALCLL GCLLSHAAAA PSPIIKFPGD VAPKTDKELA VQYLNTFYGC PKESCNLFVL KDTLKKMQKF  81 FGLPQTGDLD QNTIETMRKP RCGNPDVANY NFFPRKPKWD KNQITYRIIG YTPDLDPETV DDAFARAFQV WSDVTPLRFS  161 RIHDGEADIM INFGRWEHGD GYPFDGKDGL LAHAFAPGTG VGGDSHFDDD ELWTLGEGQV VRVKYGNADG EYCKFPFLFN  241 GKEYNSCTDT GRSDGFLWCS TTYNFEKDGK YGFCPHEALF TMGGNAEGQP CKFPFRFQGT SYDSCTTEGR TDGYRWCGTT  321 EDYDRDKKYG FCPETAMSTV GGNSEGAPCV FPFTFLGNKY ESCTSAGRSD GKMWCATTAN YDDDRKWGFC PDQGYSLFLV  401 AAHEFGHAMG LEHSQDPGAL MAPIYTYTKN FRLSQDDIKG IQELYGASPD IDLGTGPTPT LGPVTPEICK QDIVFDGIAQ  481 IRGEIFFFKD RFIWRTVTPR DKPMGPLLVA TFWPELPEKI DAVYEAPQEE KAVFFAGNEY WIYSASTLER GYPKPLTSLG  561 LPPDVQRVDA AFNWSKNKKT YIFAGDKFWR YNEVKKKMDP GFPKLIADAW NAIPDNLDAV VDLQGGGHSY FFKGAYYLKL  641 ENQSLKSVKF GSIKSDWLGC |
| --- |

Peptides:

| No | Peptide | Peptide ID | Length | Mass | Charge | Intensity | Missing |
| --- | --- | --- | --- | --- | --- | --- | --- |
| 1 | IIGYTPDLDPETVDDAFAR | MMP2_128-146 | 19 | 2089.00 | 2,3 | 13.10 | 51.3% |
| 2 | AFQVWSDVTPLR | MMP2_147-158 | 12 | 1399.72 | 2 | 13.74 | 43.3% |
| 3 | IHDGEADIMINFGR | MMP2_162-175 | 14 | 1568.74 | 3 | 8.10 | 76.6% |

## NOP56, Nucleolar protein 56 ([O00567, NOP56_HUMAN](https://www.uniprot.org/uniprot/O00567))

Coverage: 30.0% (178/594)

| 1 MVLLHVLFEH AVGYALLALK EVEEISLLQP QVEESVLNLG KFHSIVRLVA FCPFASSQVA LENANAVSEG VVHEDLRLLL  81 ETHLPSKKKK VLLGVGDPKI GAAIQEELGY NCQTGGVIAE ILRGVRLHFH NLVKGLTDLS ACKAQLGLGH SYSRAKVKFN  161 VNRVDNMIIQ SISLLDQLDK DINTFSMRVR EWYGYHFPEL VKIINDNATY CRLAQFIGNR RELNEDKLEK LEELTMDGAK  241 AKAILDASRS SMGMDISAID LINIESFSSR VVSLSEYRQS LHTYLRSKMS QVAPSLSALI GEAVGARLIA HAGSLTNLAK  321 YPASTVQILG AEKALFRALK TRGNTPKYGL IFHSTFIGRA AAKNKGRISR YLANKCSIAS RIDCFSEVPT SVFGEKLREQ  401 VEERLSFYET GEIPRKNLDV MKEAMVQAEE AAAEITRKLE KQEKKRLKKE KKRLAALALA SSENSSSTPE ECEEMSEKPK  481 KKKKQKPQEV PQENGMEDPS ISFSKPKKKK SFSKEELMSS DLEETAGSTS IPKRKKSTPK EETVNDPEEA GHRSGSKKKR  561 KFSKEEPVSS GPEEAVGKSS SKKKKKFHKA SQED |
| --- |

Peptides:

| No | Peptide | Peptide ID | Length | Mass | Charge | Intensity | Missing |
| --- | --- | --- | --- | --- | --- | --- | --- |
| 1 | VLLGVGDPK | NOP56_91-99 | 9 | 878.52 | 2 | 50.05 | 49.1% |
| 2 | GLTDLSACK | NOP56_135-143 | 9 | 888.44 | 2 | 19.83 | 34.1% |
| 3 | IINDNATYCR | NOP56_203-212 | 10 | 1163.54 | 2 | 18.16 | 44.1% |
| 4 | LAQFIGNR | NOP56_213-220 | 8 | 899.50 | 2 | 21.88 | 15.9% |
| 5 | VVSLSEYR | NOP56_271-278 | 8 | 933.49 | 2 | 77.48 | 2.1% |
| 6 | QSLHTYLR | NOP56_279-286 | 8 | 998.53 | 2 | 7.71 | 65.5% |
| 7 | MSQVAPSLSALIGEAVGAR | NOP56_289-307 | 19 | 1837.97 | 2,3 | 21.34 | 3.1% |
| 8 | LIAHAGSLTNLAK | NOP56_308-320 | 13 | 1289.75 | 2,3 | 18.47 | 1.3% |
| 9 | YPASTVQILGAEK | NOP56_321-333 | 13 | 1357.72 | 2 | 12.16 | 12.8% |
| 10 | YGLIFHSTFIGR | NOP56_348-359 | 12 | 1391.73 | 2,3 | 11.83 | 29% |
| 11 | IDCFSEVPTSVFGEK | NOP56_382-396 | 15 | 1638.76 | 2,3 | 22.57 | 1.8% |
| 12 | LSFYETGEIPR | NOP56_405-415 | 11 | 1292.64 | 2 | 42.74 | 4.7% |
| 13 | LSFYETGEIPRK | NOP56_405-416 | 12 | 1420.74 | 3 | 11.44 | 32.9% |
| 14 | EAMVQAEEAAAEITR | NOP56_423-437 | 15 | 1599.76 | 2 | 20.58 | 71.3% |
| 15 | EETVNDPEEAGHR | NOP56_541-553 | 13 | 1463.63 | 2,3 | 16.32 | 58% |
| 16 | EEPVSSGPEEAVGK | NOP56_565-578 | 14 | 1395.65 | 2 | 16.68 | 54.4% |
