# Supplementary material for: A Proteomic Signature for Human Papillomavirus–Associated Oropharyngeal Squamous Cell Carcinoma Predicts Patients at High Risk of Recurrence
Source: Cancer Res Commun. 2025 Apr 9;5(4):580–93. doi: 10.1158/2767-9764.CRC-23-0460 (PMC11979894; doi:10.1158/2767-9764.CRC-23-0460)
Supplement: Figure S2 — Top 15 cellular functions and pathways for DAPeps in tumor vs to NAT samples [file crc-23-0460_figure_s2_suppsf2.pptx]

## Slide 1
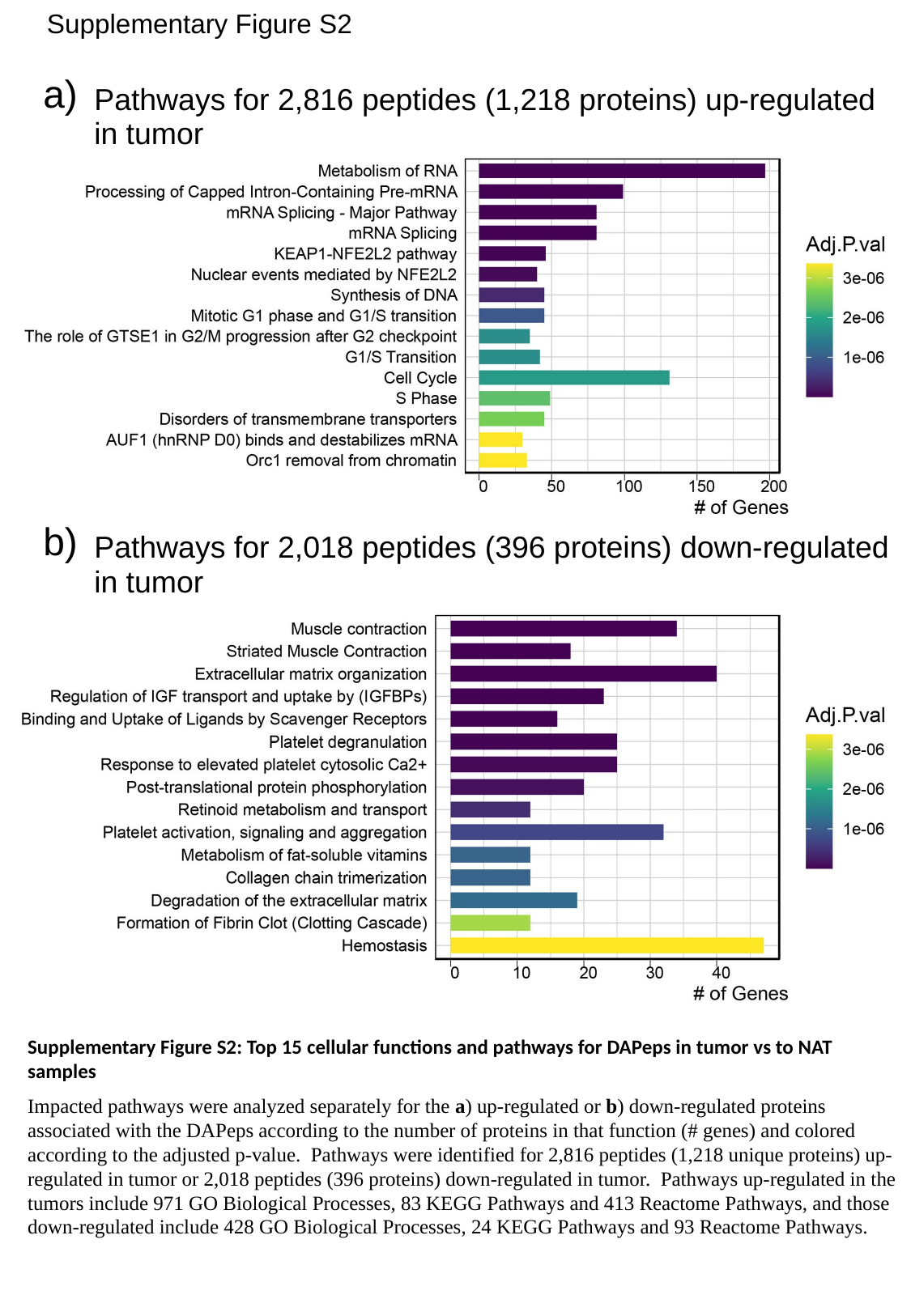

Supplementary Figure S2
a)
Pathways for 2,816 peptides (1,218 proteins) up-regulated in tumor
b)
Pathways for 2,018 peptides (396 proteins) down-regulated in tumor
Supplementary Figure S2: Top 15 cellular functions and pathways for DAPeps in tumor vs to NAT samples
Impacted pathways were analyzed separately for the a) up-regulated or b) down-regulated proteins associated with the DAPeps according to the number of proteins in that function (# genes) and colored according to the adjusted p-value. Pathways were identified for 2,816 peptides (1,218 unique proteins) up-regulated in tumor or 2,018 peptides (396 proteins) down-regulated in tumor. Pathways up-regulated in the tumors include 971 GO Biological Processes, 83 KEGG Pathways and 413 Reactome Pathways, and those down-regulated include 428 GO Biological Processes, 24 KEGG Pathways and 93 Reactome Pathways.
