# Supplementary material for: A Proteomic Signature for Human Papillomavirus–Associated Oropharyngeal Squamous Cell Carcinoma Predicts Patients at High Risk of Recurrence
Source: Cancer Res Commun. 2025 Apr 9;5(4):580–93. doi: 10.1158/2767-9764.CRC-23-0460 (PMC11979894; doi:10.1158/2767-9764.CRC-23-0460)
Supplement: Figure S3 — Distribution of the quantified peptides among the number of patient tumor samples. [file crc-23-0460_figure_s3_suppsf3.pptx]

## Slide 1
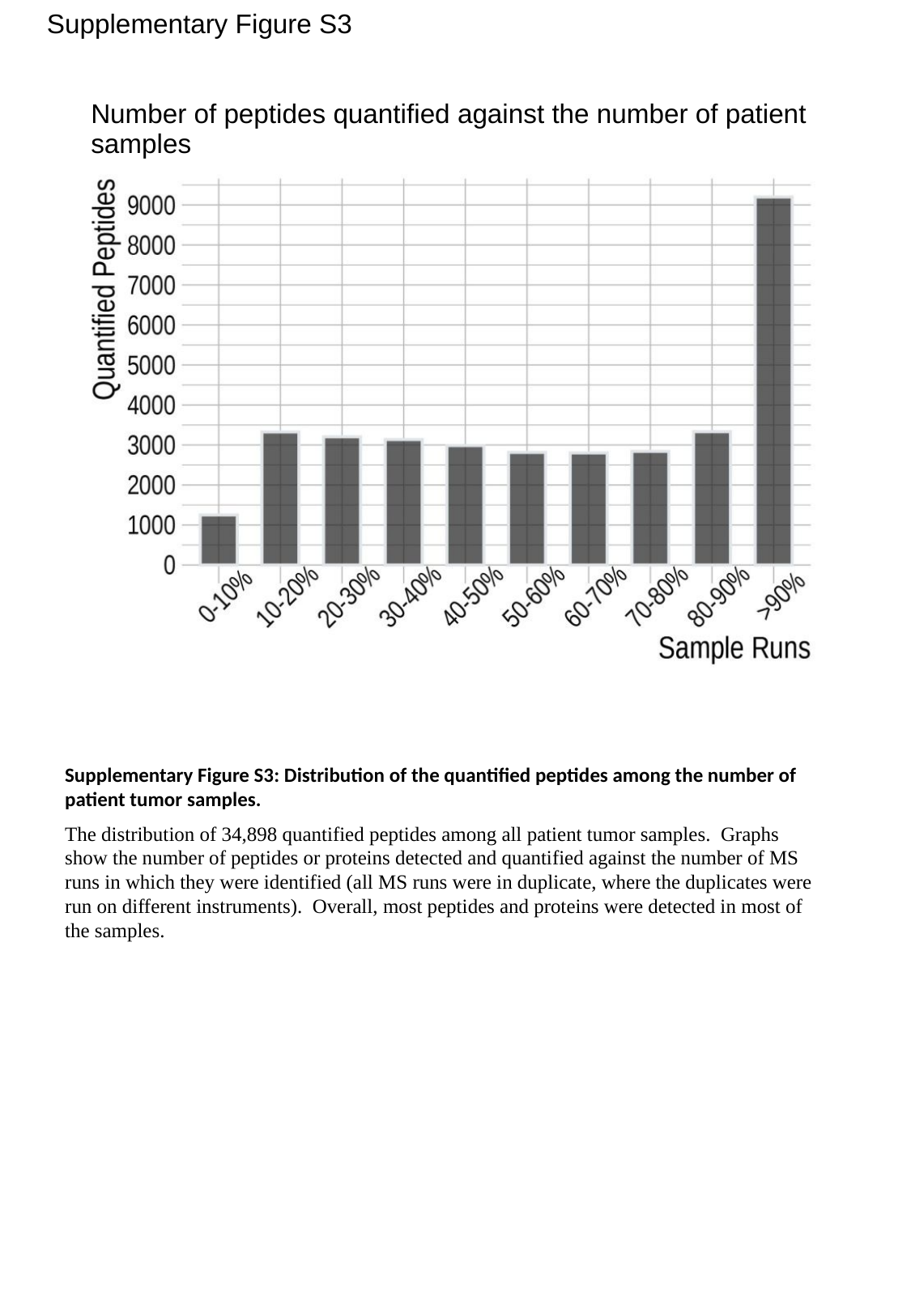

Supplementary Figure S3
Number of peptides quantified against the number of patient samples
Supplementary Figure S3: Distribution of the quantified peptides among the number of patient tumor samples.
The distribution of 34,898 quantified peptides among all patient tumor samples. Graphs show the number of peptides or proteins detected and quantified against the number of MS runs in which they were identified (all MS runs were in duplicate, where the duplicates were run on different instruments). Overall, most peptides and proteins were detected in most of the samples.
