# Supplementary material for: A Proteomic Signature for Human Papillomavirus–Associated Oropharyngeal Squamous Cell Carcinoma Predicts Patients at High Risk of Recurrence
Source: Cancer Res Commun. 2025 Apr 9;5(4):580–93. doi: 10.1158/2767-9764.CRC-23-0460 (PMC11979894; doi:10.1158/2767-9764.CRC-23-0460)
Supplement: Figure S4 — Top 15 cellular functions and pathways for DAPep tumor samples from recurrence vs non-recurrence [file crc-23-0460_figure_s4_suppsf4.pptx]

## Slide 1
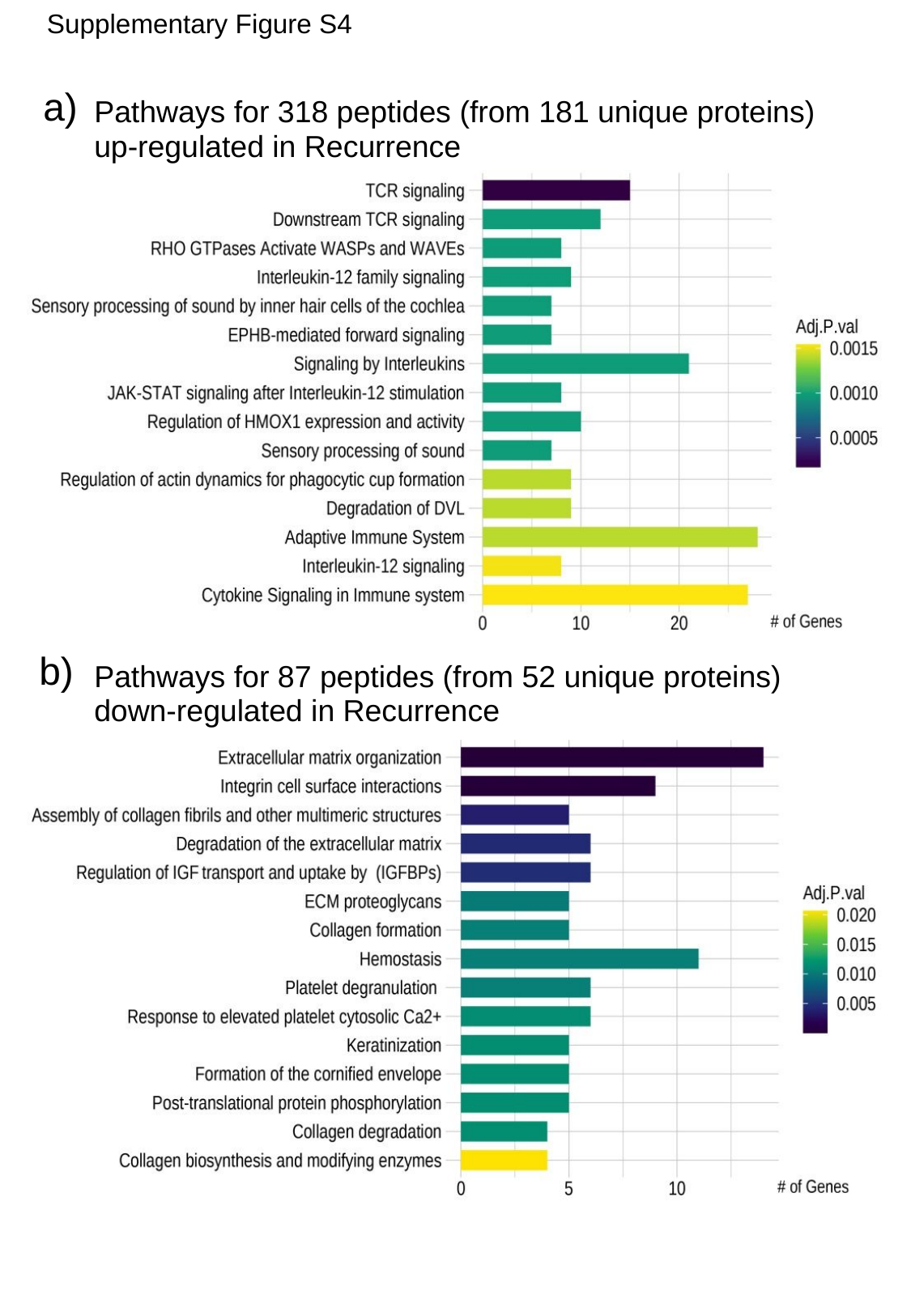

Supplementary Figure S4
a)
Pathways for 318 peptides (from 181 unique proteins) up-regulated in Recurrence
b)
Pathways for 87 peptides (from 52 unique proteins) down-regulated in Recurrence

## Slide 2
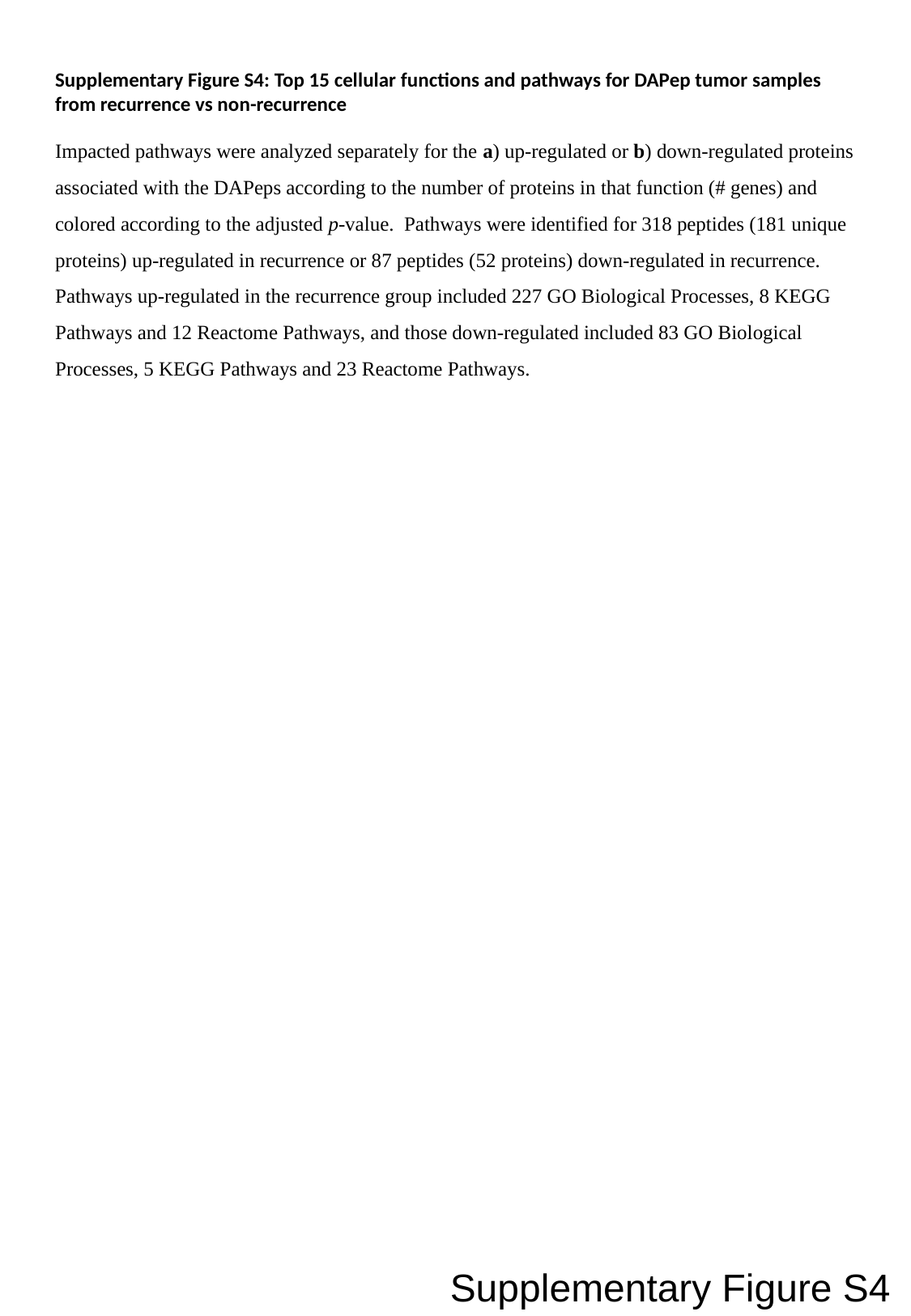

Supplementary Figure S4: Top 15 cellular functions and pathways for DAPep tumor samples from recurrence vs non-recurrence
Impacted pathways were analyzed separately for the a) up-regulated or b) down-regulated proteins associated with the DAPeps according to the number of proteins in that function (# genes) and colored according to the adjusted p-value. Pathways were identified for 318 peptides (181 unique proteins) up-regulated in recurrence or 87 peptides (52 proteins) down-regulated in recurrence. Pathways up-regulated in the recurrence group included 227 GO Biological Processes, 8 KEGG Pathways and 12 Reactome Pathways, and those down-regulated included 83 GO Biological Processes, 5 KEGG Pathways and 23 Reactome Pathways.
 Supplementary Figure S4
