# Supplementary material for: A Proteomic Signature for Human Papillomavirus–Associated Oropharyngeal Squamous Cell Carcinoma Predicts Patients at High Risk of Recurrence
Source: Cancer Res Commun. 2025 Apr 9;5(4):580–93. doi: 10.1158/2767-9764.CRC-23-0460 (PMC11979894; doi:10.1158/2767-9764.CRC-23-0460)
Supplement: Figure S5 — Multivariate Cox regression models incorporating the 26-peptide signature [file crc-23-0460_figure_s5_suppsf5.pptx]

## Slide 1
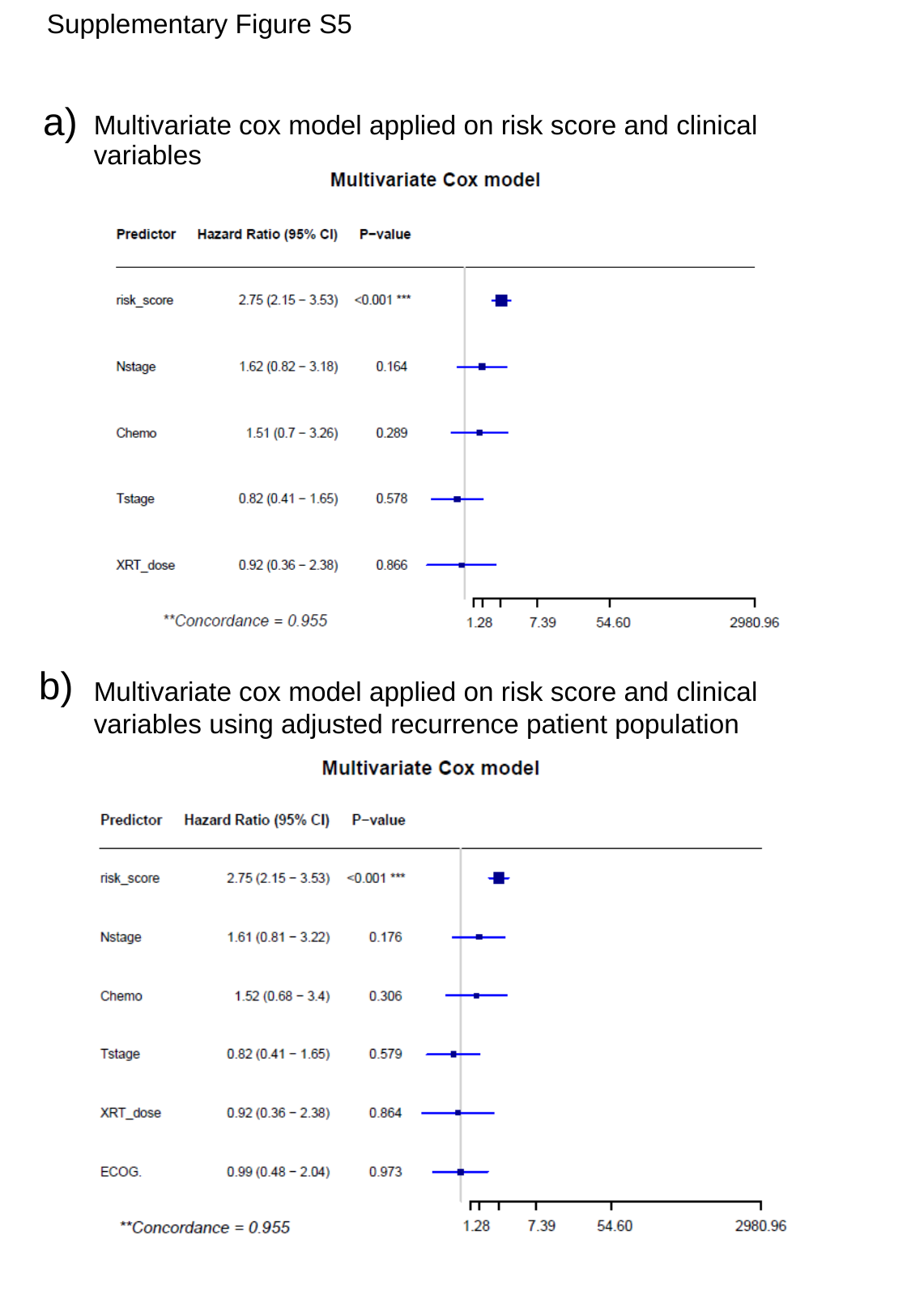

Supplementary Figure S5
a)
Multivariate cox model applied on risk score and clinical variables
b)
Multivariate cox model applied on risk score and clinical variables using adjusted recurrence patient population

## Slide 2
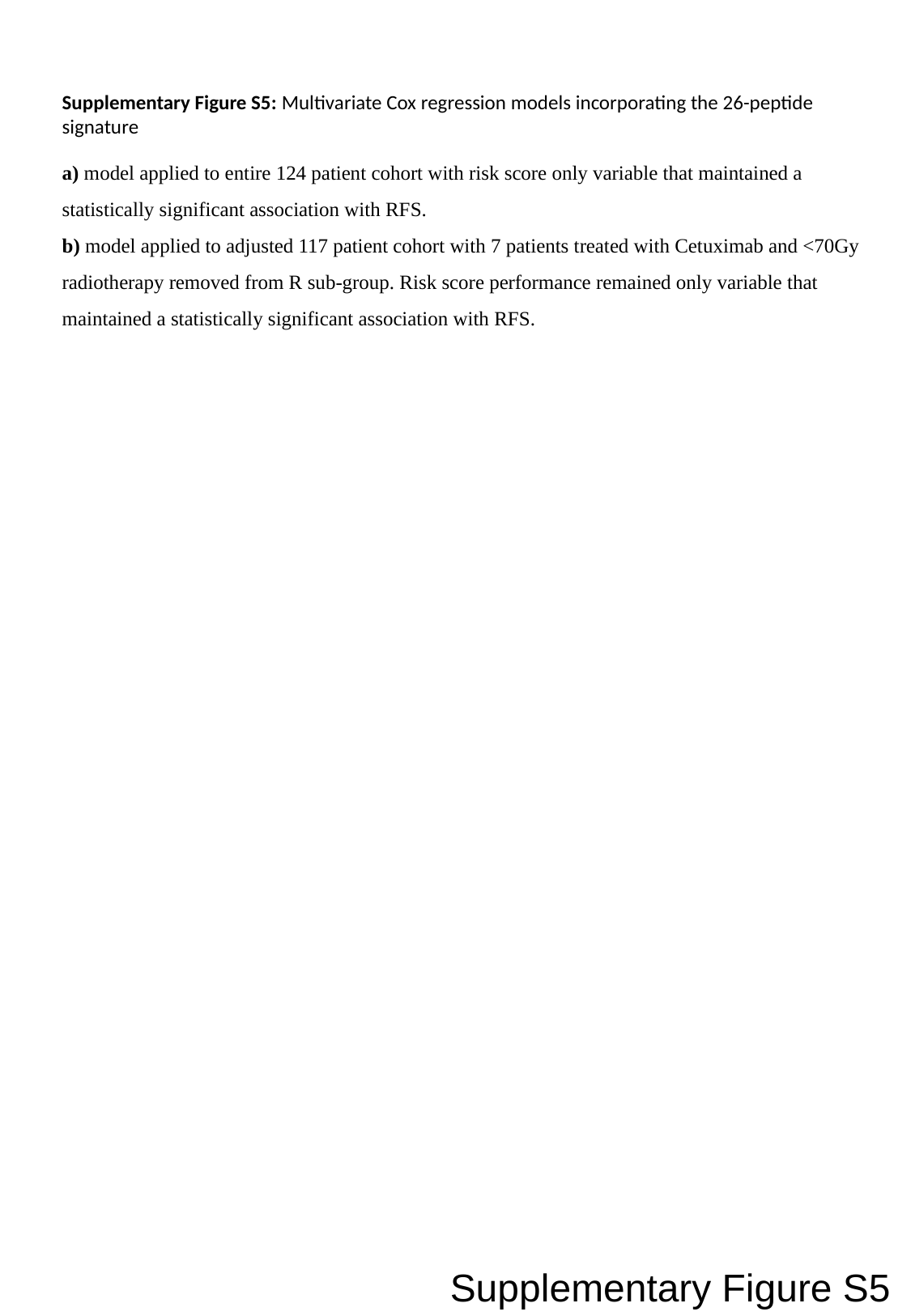

Supplementary Figure S5: Multivariate Cox regression models incorporating the 26-peptide signature
a) model applied to entire 124 patient cohort with risk score only variable that maintained a statistically significant association with RFS.
b) model applied to adjusted 117 patient cohort with 7 patients treated with Cetuximab and <70Gy radiotherapy removed from R sub-group. Risk score performance remained only variable that maintained a statistically significant association with RFS.
 Supplementary Figure S5
