# Supplementary material for: A Proteomic Signature for Human Papillomavirus–Associated Oropharyngeal Squamous Cell Carcinoma Predicts Patients at High Risk of Recurrence
Source: Cancer Res Commun. 2025 Apr 9;5(4):580–93. doi: 10.1158/2767-9764.CRC-23-0460 (PMC11979894; doi:10.1158/2767-9764.CRC-23-0460)
Supplement: Figure S6 — 26-peptide signature stratifies HPV+ OPSCC patients into three risk groups for recurrence free survival (RFS) [file crc-23-0460_figure_s6_suppsf6.pptx]

## Slide 1
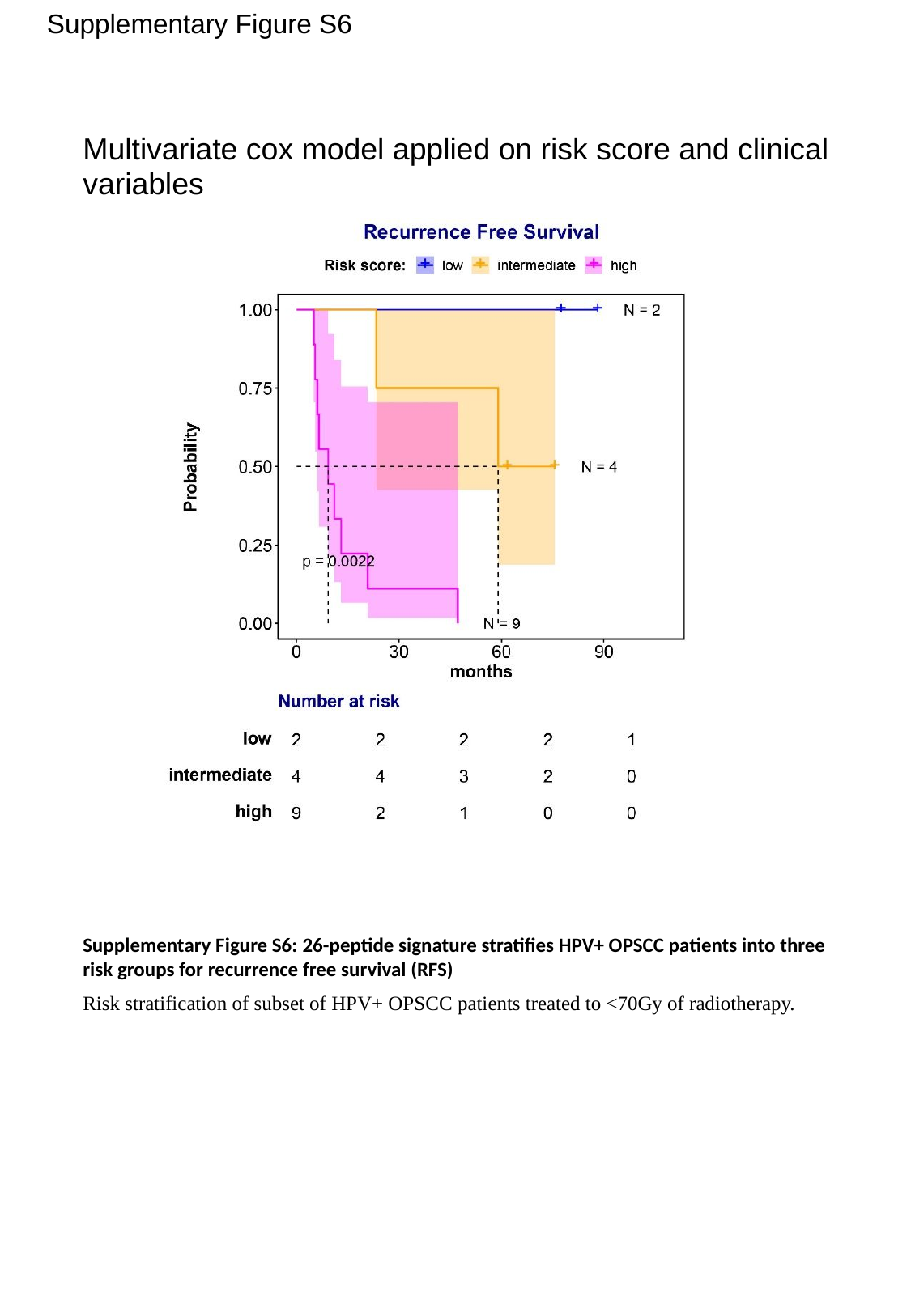

Supplementary Figure S6
Multivariate cox model applied on risk score and clinical variables
Supplementary Figure S6: 26-peptide signature stratifies HPV+ OPSCC patients into three risk groups for recurrence free survival (RFS)
Risk stratification of subset of HPV+ OPSCC patients treated to <70Gy of radiotherapy.
